# Progress on the Effects of Microplastics on Aquatic Crustaceans: A Review

**DOI:** 10.3390/ijms24065523

**Published:** 2023-03-14

**Authors:** Siyi Zhang, Haodi Wu, Jing Hou

**Affiliations:** MOE Key Laboratory of Resources and Environmental Systems Optimization, College of Environmental Science and Engineering, North China Electric Power University, Beijing 102206, China

**Keywords:** microplastic, aquatic crustacean, life history, behavior, physiological function, response mechanism

## Abstract

It is impossible to overlook the effects of microplastics on aquatic life as they continuously accumulate in aquatic environments. Aquatic crustaceans, as both predator and prey, play an important role in the food web and energy transmission. It is of great practical significance to pay attention to the toxic effects of microplastics on aquatic crustaceans. This review finds that most studies have shown that microplastics negatively affect the life history, behaviors and physiological functions of aquatic crustaceans under experimental conditions. The effects of microplastics of different sizes, shapes or types on aquatic crustaceans are different. Generally, smaller microplastics have more negative effects on aquatic crustaceans. Irregular microplastics have more negative effects on aquatic crustaceans than regular microplastics. When microplastics co-exist with other contaminants, they have a greater negative impact on aquatic crustaceans than single contaminants. This review contributes to rapidly understanding the effects of microplastics on aquatic crustaceans, providing a basic framework for the ecological threat of microplastics to aquatic crustaceans.

## 1. Introduction

With the increase in plastic production, use and emissions, 275 million metric tons of plastic waste were generated in 192 coastal countries, of which 4.8–12.7 million metric tons entered the sea in 2010 [1]. As of 2015, approximately 6300 million metric tons of plastic waste had been generated, and by 2050, nearly 12,000 million metric tons of plastic waste is expected to enter landfills or the natural environment [2]. Plastics could form microplastics through mechanical [3], photochemical [4] and biological [5] processes. Microplastics were first proposed in 2004 [6] and were considered as plastic debris <5 mm, whilst plastic debris <100 nm were considered nanoplastics [7]. Currently, microplastics have been detected in the oceans [8,9,10], freshwater [11], underground water [12], rivers [13], polar regions [14] and even in drinking water [15]. Microplastics have also been detected in aquatic organisms such as seabirds [16], turtles [17], zooplankton [18], fishes [19], mammals [20], crustaceans [21] and bivalves [22]. Studies have found that microplastics have negative effects on aquatic organisms, such as changes in feeding ability [23] and swimming behavior [24], malformation [25], oxidative stress response [26], growth and development toxicity [27], reproductive toxicity [28], neurotoxicity [29], immunotoxicity [30], etc.

As a ubiquitous aquatic organism in freshwater and marine environments, aquatic crustaceans are widely used in experimental studies due to their wide distribution, rapid reproduction and toxicity sensitivity. Aquatic crustaceans play an important role in the food web and energy transmission. They can be predators to plants, algae and other smaller creatures, as well as food sources for other prey. Aquatic crustaceans such as *Neomysis integer* [31] and *Gammarus duebeni* [32] could act as predators, while other aquatic crustaceans such as *Tigriopus fulvus* [33], *Daphnia magna* [34,35] and *Neomysis* spp. [36] could serve as food for other creatures. Aquatic crustaceans could also act as carriers of microplastics in the food chain. For example, in the food chain of *Raphidocelis subcapitata–D. magna*–*Pimephales promelas*, *D. magna* act as both predator and prey, and microplastics were found to be transferred to the highest nutritional level of the food chain [34]. The aquatic crustaceans commonly used in experimental studies are Cladocera, Decapoda, Copepoda, Anostraca, Amphipoda, Cirripedia, Isopoda, etc. The Cladocera are key zooplankton species in aquatic ecosystems that could be eaten by any freshwater predator [37], among which the most typical, *D. magna,* has the advantages of a large size, short life cycle, high fecundity and parthenogenesis [38]. Decapoda represent a diverse taxon within the aquatic crustaceans, which are rich in species and play an important role in ecology and the economy [39], and some Decapoda are globally important seafood products with high value in commercial fisheries and aquaculture [40]. Copepoda, the second largest crustacean taxa, are widely distributed and play an important role in the transport of contaminants across the food chain [41]. Anostraca are unquestionably the most taxonomically diverse group at all levels [42]. Amphipoda are widely distributed in marine and freshwater environments and represent the dominant macroinvertebrates in biomass in many river communities [43]. Cirripedia are one of the most common marine crustaceans [44]. Additionally, Isopoda could survive in a variety of water environments and are one of the most abundant groups in marine ecosystems [45].

**Figure 1:**
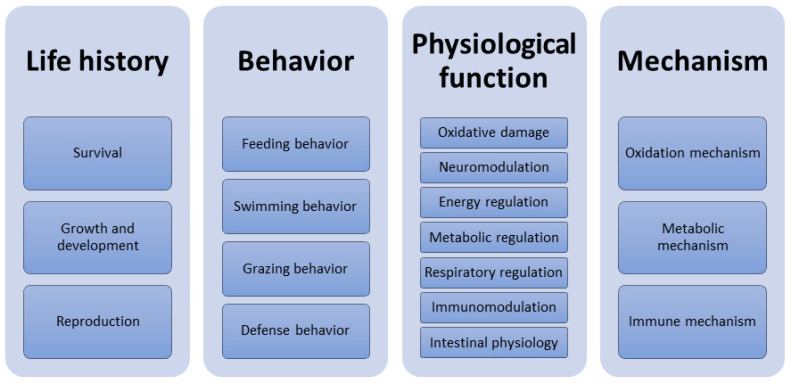
Effects of microplastics on aquatic crustaceans.

To date, experimental studies on the effects of microplastics on aquatic organisms have mainly focused on shellfish and fish, whilst aquatic crustaceans have not been comprehensively reviewed. Therefore, we specifically reviewed the effects of microplastics on aquatic crustaceans in terms of life history, behaviors, physiological functions and molecular response mechanisms (Figure 1). The keywords “microplastics”, “crustacean”, “Cladocera (cladoceran)”, “Decapoda (decapod)”, “Copepoda (copepod)”, “Anostraca”, “Amphipoda (amphipod)”, “Cirripedia (barnacle)” and “Isopoda (isopod)” were retrieved jointly in the database. The aquatic crustaceans were classified by referring to the classification of the National Center for Biotechnology Information (NCBI). This review only summarized experimental studies with independent experiments, and did not review other external factors such as pH, temperature, etc. A total of 239 papers meeting the above criteria were retrieved. Among them, there were 200 papers (83.68%) on life history (Table S1), 61 papers (25.52%) on behaviors (Table S2), 79 papers (33.05%) on physiological functions (Table S3) and 57 papers (23.85%) on molecular response mechanisms (Table S4). This review contributes to rapidly understanding the effects of microplastics on aquatic crustaceans, providing a basic framework for the ecological threat of microplastics to aquatic crustaceans.

## 2. Effects of Microplastics on Life History of Aquatic Crustaceans

**Figure 2:**
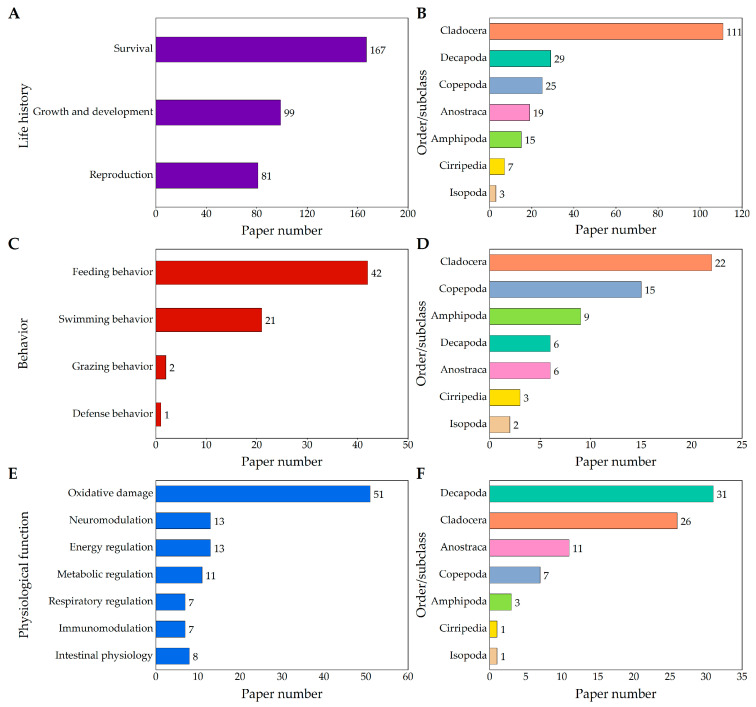
The number of papers on the effects of microplastics on the life history, behavior and physiological function of aquatic crustacean. The number of papers examining the life history, behavior and physiological function of aquatic crustacean impacted by microplastics are shown in (**A**,**C**,**E**), respectively. The number of papers on aquatic species involved in the life history, behavior and physiological function of aquatic crustacean are shown in (**B**,**D**,**F**), respectively.

Two hundred papers dealing with the study of the effects of microplastics on the life history of aquatic crustaceans were found, including survival (167 papers, 83.50%), growth and development (99 papers, 49.50%) and reproduction (81 papers, 40.50%) (Figure 2A). Among these, 111 papers focused on Cladocera (8 species), 29 papers on Decapoda (17 species), 25 papers on Copepoda (16 species), 19 papers on Anostraca (3 species), 15 papers on Amphipoda (9 species), 7 papers on Cirripedia (10 species) and 3 papers on Isopoda (2 species) (Figure 2B). The most studied Cladocera were *D. magna* and *Daphnia pulex*. The most studied Decapoda were *Litopenaeus vannamei* and *Macrobrachium nipponense*. The most studied Copepoda were *Tigriopus japonicus* and *Acartia tonsa*. The *Artemia franciscana* and *Amphibalanus amphitrite* were the most studied Anostraca and Cirripedia, respectively. The most extensively utilized types of microplastics were polystyrene (PS) (118 papers, 59.00%), polyethylene (PE) (44 papers, 22.00%), polypropylene (PP) (13 papers, 6.50%), polyethylene terephthalate (PET) (13 papers, 6.50%) and polyvinyl chloride (PVC) (8 papers, 4.00%), whilst the most extensively utilized shapes were microspheres (76 papers, 38.00%), microbeads (41 papers, 20.50%), microfibers (21 papers, 10.50%) and fragments (14 papers, 7.00%).

### 2.1. Survival

For Cladocera, *D. magna* was the most concerned species. It has been found that 52 nm [35] and 100–120 nm amino-PS (PS-${\mathrm{NH}}_{2}$) [46,47] reduced *D. magna* survival in a dose-dependent manner within the range of 1–400 mg/L. However, 0–1.5 μm PS significantly increased *D. magna* immobilization followed by 10–60 and 60–230 μm PS [48]. The 48-h median effect concentrations (${\mathrm{EC}}_{50}$) of amidine PS and carboxyl PS were 36.2 ± 4.1 mg/L and 111.4 ± 15.2 mg/L [49], the 24 h ${\mathrm{EC}}_{50}$ of PS-${\mathrm{NH}}_{2}$ and carboxylated-PS (PS-COOH) was 25.8 mg/L and 36.3 mg/L [23], whilst the 48 h median lethal concentrations (${\mathrm{LC}}_{50}$) of plain PS, negative PS-${\mathrm{NH}}_{2}$ and PS-COOH was 5.24, 8.56 and 20.2 mg/L to *D. magna* [50], respectively. Pristine PS could significantly decrease *D. magna* survival, while aged PS caused no significant effects [51]. PS and deltamethrin (DM) produced synergistic effects on *D. magna* survival [52]. The same went for PS and glyphosate (Gly) [53]. On the contrary, humic substances reduced the PS-${\mathrm{NH}}_{2}$ toxicity to *D. magna* survival based on their composition and corona formation [46]. Similarly, humic acid could alleviate the toxicity of PS-${\mathrm{NH}}_{2}$ [47]. Additionally, the combined effects of PS combined with the heavy metals lead (Pb), copper (Cu), cadmium (Cd) and nickel (Ni) on *D. magna* survival changed from antagonistic to additive as the concentration of PS increased [54]. The 48 h ${\mathrm{EC}}_{50}$ of 37.24 ± 11.76 μm PE fragments and 37.05 ± 3.96 μm PE beads to *D. magna* was 3.9 mg/L and 323 mg/L, respectively [55]. The survival rate of *D. magna* caused by 17.23 ± 3.43 µm PE fragments, 34.43 ± 13.09 µm fragments and 40–48 µm beads was 20%, 60% and 90%, respectively [56]. Pristine PE showed more negative effects on *D. magna* survival than biofouled PE, and found that the ${\mathrm{LC}}_{50}$ of pristine PE to *D. magna* dropped from 485 to 1.26 mg/L during a period of 72 h to 7 days [57]. It was found that 37.24 ± 11.76 μm PE fragments and benzophenone-3 (BP-3) had synergistic effects on reducing the *D. magna* survival, the 48 h ${\mathrm{EC}}_{50}$ of PE and mixture of PE and BP-3 was 3.9 and 0.99 mg/L [55]. For other Cladocera species, 48 h ${\mathrm{LC}}_{50}$ of 75 nm PS to *D. pulex* was 76.69 mg/L [58]. The forty-eight-hour LC_50_ of PE beads and polyester fibers to *Ceriodaphnia dubia* was 2.2 mg/L and 1.5 mg/L, respectively [24]. Primary microplastics reduced the *C. dubia* survival more than secondary microplastics [59].

For Decapoda, 75 nm PS significantly reduced *M. nipponense* survival in a dose-dependent manner within the range of 5–40 mg/L [60]. It was also found that 1 and 10 μm PS reduced *Neomysis awatschensis* survival in a dose-dependent manner within the range of 1 × $10^{3}$–5 × $10^{5}$ particles/mL [61]. The 96-h ${\mathrm{LC}}_{50}$ of 75 nm PS to *M. nipponense* was 396.391 mg/L [62]. PS–COOH reduced *Neomysis japonic* survival more than PS within the range of 10–1250 μg/L [63]. PE reduced the survival of shrimps (*Penaeus monodon*, *Marsupenaeus japonicas*, *L. vannamei*) in dose-dependent manner within the range of 25–300 mg/L [64]. PE significantly reduced *L. vannamei* survival at 5000 μg/L [65] or at 0.5 and 1 μg/g shrimp [66]. The 93 μm fibers reduced *Palaemonetes pugio* survival more than fragment and spheres [67]. High-density polyethylene (HDPE) and malathion (MLT) had synergistic effects on decreasing *Minuca ecuadoriensis* survival [68].

For Copepoda, 0.05 μm PS caused the highest negative effects on *T. japonicus* survival followed by 0.5 μm PS and 6 μm PS had no effects within the range of 0.125–25 mg/L [69]. Chlorpyrifos (CPF) increased the effects caused by HDPE on reducing *A. tonsa* survival [70]. PE and triclosan (TCS) produced synergistic effects on reducing *A. tonsa* survival [71]. For Anostraca, the 48 h ${\mathrm{LC}}_{50}$ of 50–70 nm PS and 100–120 nm PS to *Artemia salina* was 4.82 mg/L and 8.79 mg/L, respectively [72]. Plain PS, PS-COOH and PS-${\mathrm{NH}}_{2}$ co-existed with nano-${\mathrm{TiO}}_{2}$ altered *A. salina* survival [73]. For Amphipoda, PE significantly reduced *Hyalella azteca* survival at 1000 and 10,000 plastics/mL [74]. Tire wear particles reduced *H. azteca* survival in a dose-dependent within the range of 0–15,000 particles/mL [75]. For Anostraca, the 24-h ${\mathrm{EC}}_{50}$ of amidine PS and carboxyl PS to *Thamnocephalus platyurus* was 194.8 ± 17.6 mg/L and 318.2 ± 66.9 mg/L, respectively [49]. Additionally, for Cirripedia, PVC significantly reduced *A. amphitrite* survival compared to PS and polymethyl methacrylate (PMMA) at 15 and 25 mg/L [76].

### 2.2. Growth and Development

For Cladocera, *D. magna* was the most concerned species. PS significantly reduced the *D. magna* body size at 2000 particles/mL [77,78] and within the range of 5–100 mg/L [79]. Six-micrometer PS spheres significantly reduced the *D. magna* body length at 500 particles/mL [80]. Ten-micrometer PS affected *D. magna* body length at 0.125, 1.25 and 12.5 mg/L, while one-micrometer PS had no significant effect [81]. PS significantly reduced *D. magna* growth at 1 and 2 mg/L, and PS co-existed with TCS or methyl-triclosan (MTCS) caused more growth reduction than that which co-existed with triclocarban (TCC) [82]. PE reduced the *D. magna* growth-like body size, lifespan, etc., in a dose-dependent manner at 5, 20 and 40 mg/L [83]. PE fragments of 17.23 ± 3.43 µm significantly decreased the *D. magna* body length, while 34.43 ± 13.09 µm fragments and 40–48 µm beads caused no effects [56]. PE co-existed with DM resulted higher negative effects on *D. magna* growth than single PE [52]. For other Cladocera species, 75 nm PS affected *D. pulex* growth within the range of 0.1–2 mg/L [58]. Moreover, 0.7 µm PS caused more negative effects on growth of *D. pulex* and *Moina macrocopa* than 1 µm PS [84]. PE beads and polyester fibers significantly reduced the *C. dubia* body size at 2000 μg/L, and 500 and 1000 μg/L, respectively [24]. PE co-existed with Cd reduced *Moina monogolica* growth more significantly than single PE around 16% and 10% when PE was 300 μg/L [85]. On the contrary, PE and Cu had an antagonistic interaction on *Daphnia carinata* molting frequency [86].

For Decapoda, 75 nm PS affected the *M. nipponense* growth within the range of 5–40 mg/L [60]. PS and PS-COOH induced *Neomysis japonica* growth inhibition within the range of 250–6250 μg/L, and PS-COOH had significantly greater growth inhibition [63]. Both PS and PE reduced the length and weight of *Macrobrachium rosenbergii* in a dose-dependent manner at 1, 5 and 10 mg/100 g in food [87]. PP fibers significantly affected the growth of *Carcinus maenas* at 0.3%, 0.6% and 1% in food [88]. For Copepoda, 0.05 μm PS significantly reduced the *Paracyclopina nana* growth rate at 10 and 20 mg/L, while 0.5 and 6 μm PS had no significant effect [89]. Whilst the 0.05 μm PS significantly delayed the development time of *T. japonicus* in F0 and F1 generations at 1.25 mg/L, the 0.5 μm PS significantly delayed development in F1 generation at 25 mg/L and the 6 μm ones caused no effects [69]. Additionally, 6.58 µm PS significantly delayed the development time of *Nitokra lacustris pacifca* at 700 beads/mL [90]. For Anostraca, PS-${\mathrm{NH}}_{2}$ significantly reduced body length of *A. franciscana* at 1 and 10 mg/L [91]. PS and PE significantly reduced the *Artemia parthenogenetica* growth rate and body length at 100 mg/L, and PE had a higher inhibition effect on the growth rate [92]. Additionally, for Amphipoda, PS significantly inhibited the *Gammarus pulex* growth in a dose-dependent manner within the range of 10–40% PS weight in sediment, but had no effect on *H. azteca* [93]. However, PE particles (5000 and 10,000 particles/mL) and PP fibers (45 and 90 fibers/mL) significantly reduced the dry weight of *H. azteca* [74]. Tire wear particles significantly decreased the growth of *H. azteca* at 1000 and 2000 particles/mL [75].

### 2.3. Reproduction

For Cladocera *D. magna*, 200 nm PS decreased the number of broods produced by *D. magna* compared to 600 nm PS [94]. Pristine PS and aged PS significantly inhibited the *D. magna* reproduction at the $10^{5}$ particles/mL concentration level [51]. Moreover, 10,000 particles/mL pristine PS led to the extinction of *D. magna* in F2 generation [78], and 2000 and 10,000 particles/mL secondary PS led to extinction in F3 and F0 generations [77], respectively. Interestingly, 1 and 10 μm PS unexpectedly significantly increased *D. magna* reproduction at 12.5 mg/L [81]. The co-existence of PS and other contaminants could cause synergistic toxicity on *D. magna* reproduction, such as PS and DM [52], PS and Gly [53], PS and benzo(a)pyrene (BaP) [94] and PS-COOH and zinc (Zn) [95]. PE reduced the *D. magna* reproduction in a dose-dependent manner at 5, 20 and 40 mg/L [83]. Smaller PE (17.23 ± 3.43 µm fragments) significantly reduced *D. magna* reproduction compared to larger PE (34.43 ± 13.09 µm fragments and 40–48 µm beads) [56]. Secondary PE had stronger effects on *D. magna* reproduction than primary microplastics [96]. PE decreased the number of broods produced by *D. magna* compared to PVC and PP [94]. PE and BaP had synergistic effects on reducing the *D. magna* reproduction [94]. PVC, PLA and PUR reduced the reproduction of *D. magna* within the range of 10–500 mg/L, and PVC had the greatest impacts followed by PLA and polyurethane (PUR). The 21-day ${\mathrm{EC}}_{50}$ of PVC, PLA and polyurethane (PUR) for *D. magna* reproduction was 45.5, 122 and 236 mg/L [97], respectively. For other Cladocera species, 75 nm PS reduced the *D. pulex* reproduction within the range of 0.1–2 mg/L [58], whilst the 0.7 µm PS caused stronger effects on *D. pulex* and *M. macrocopa* reproduction than 1 µm PS, and *M. macrocopa* was more sensitive [84]. PE or mixtures of PE and Cd significantly reduced *M. monogolica* reproduction [85]. PE beads and polyester fibers reduced the reproduction of *C. dubia* in a dose-dependent manner within the range of 62.5–2000 μg/L, and polyester fibers caused greater impacts [24]. Primary microplastics and secondary PE reduced the reproduction of *D. magna*, *D. pulex* and *C. dubia* within the range of 0–$10^{5}$ particles/mL, in which *D. magna* was the least sensitive species and *C. dubia* was the most sensitive species [98].

For Copepoda, 6 μm PS significantly reduced *T. japonicus* reproduction and fecundity at 0.23 mg/L [99]. Both 0.5 and 6 μm PS significantly reduced the fecundity of *T. japonicus* within the range of 0.125–25 mg/L, while 0.05 μm PS had no significant effect [69]. PS co-existed with dibutyl phthalate (DBP) and had antagonistic effects on *T. japonicu* reproduction [100]. Moreover, 0.05 μm PS reduced *P. nana* fecundity, followed by 0.5 μm PS, while 6 μm PS had no significant effect [89]. PS caused significant reproductive toxicity in *Nitokra lacustris pacifca* at 700 beads/mL [90]. PE led to the stress-induced spawning of Arctic Copepoda (*Calanus finmarchicus* and *Calanus glacialis*) at 20 particles/mL [101]. HDPE co-existed with CPF and produced significantly negative effects on *A. tonsa* reproduction [70]. PET significantly reduced the egg production of *Parvocalanus crassirostris* at 80,000 particles/mL [102]. For Amphipoda, PE significantly reduced the *H. azteca* neonate number at 5000 and 10,000 plastics/mL [74]. Tire wear particles significantly reduced the *H. azteca* reproductive output of 1000 particles/mL [75]. Additionally, for Anostraca, the polymer sphere reduced *A. franciscana* reproduction in a dose-dependent manner at 0.4, 0.8 and 1.6 mg/L [103].

Most papers showed that microplastics smaller than 500 microns had adverse effects on the life history of aquatic crustaceans. Smaller microplastics tend to have a greater effect on the survival of aquatic crustaceans than larger microplastics. The negative impacts of irregular microplastics (fibers, fragments) on the survival of aquatic crustaceans are greater than those of regular microplastics (spheres, beads). Primary or pristine microplastics have a greater negative impact on crustaceans’ survival than secondary or aged microplastics do. When microplastics co-exist with insecticides, herbicides, pesticides, anti-microbial agents, UV stabilizers and other contaminants, they have synergistic inhibiting effects on the survival of aquatic crustaceans. Generally, smaller microplastics have more significant negative effects on the growth and development of aquatic crustaceans than larger microplastics. Microfibers cause more negative effects on the growth and development of aquatic crustaceans than microspheres. When microplastics co-exist with pesticides and other contaminants, the negative effects on the growth and development of aquatic crustaceans are even greater. Smaller microplastics tend to have a higher reproductive toxicity than larger microplastics. The reproductive toxicity of fibers to aquatic crustaceans is higher than that of microspheres. When microplastics co-exist with herbicides, heavy metals and other contaminants, they have synergistic toxicity effects on the reproduction of aquatic crustaceans.

## 3. Effects of Microplastics on Behaviors of Aquatic Crustaceans

A total of 61 papers related to the effects of microplastics on aquatic crustacean behavior, including feeding behaviors (42 papers, 68.85%), swimming behaviors (21 papers, 34.43%), grazing behaviors (2 papers, 3.28%) and defense behaviors (1 paper, 1.64%) (Figure 2C). Of these, 22 papers focused on Cladocera (2 species), 15 papers on Copepoda (10 species), 9 papers on Amphipoda (6 species), 6 papers on Decapoda (5 species), 6 papers on Anostraca (2 species), 3 papers on Cirripedia (1 species) and 2 papers on Isopoda (2 species) (Figure 2D). The most studied aquatic crustaceans were *D. magna*, *Calanus helgolandicus* and *A. amphitrite*. The most extensively utilized types of microplastics were PS (34 papers, 55.74%), PE (14 papers, 22.95%), PET (3 papers, 4.92%) and PP (3 papers, 4.92%), whilst the most extensively utilized shapes were microspheres (21 papers, 34.43%), microbeads (16 papers, 26.23%), microfibers (8 papers, 13.11%) and fragments (5 papers, 8.20%).

### 3.1. Feeding Behaviors

For Copepoda, 0.5 and 10 μm PS could significantly reduce the *Pseudodiaptomus annandalei* feeding rate [104]. *C. helgolandicus* could avoid ingesting algae similar in size and/or shape to microplastics [105]. Nylon fibers led to a significant reduction in the algae intake of *C. finmarchicus* rather than nylon granules, which might work for *C. finmarchicus* avoiding microalgae that are similar in shape (i.e., chain-like *Dunaliella tertiolecta*) and size (i.e., *Scripsiella trochoidea*) to nylon fibers (10 × 30 μm) [106]. PE and oil had synergistic effects on inhibiting *Calanus hyperboreus* feeding [107]. Similarly, HDPE and CPF had more effects on reducing the *A. tonsa* feeding than each contaminant [70]. For Cladocera, PS could reduce the *D. magna* feeding capacity [108,109]. Primary PS decreased *D. magna* feeding efficiency more significantly than secondary PS [51]. PS at 40% of algal cells might have increased the algae intake of *D. magna* because *D. magna* expanded their filtration gapes when food availability declined [110]. Additionally, it has been found that *D. magna* might not distinguish similarly sized colorful PS and algae [110]. PET fibers affected algae consumption by *D. magna* more significantly than PP fibers [111]. Primary and secondary microplastics led to a 29% and 28% reduction in the food intake of *D. magna* [96]. For Decapoda, both 1 and 10 μm PS significantly reduced the feeding rate of *N. awatschensis* juveniles and adults at 5 × $10^{5}$ particles/mL [61]. For Anostraca, PS significantly reduced the microalgal feeding by *A. parthenogenetica* at 100, 1000 and 10,000 particles/mL [112]. PS-${\mathrm{NH}}_{2}$ slightly increased the filtration rate in *A. franciscana* after 7 days while decreased the filtration rate after 14 days [91].

### 3.2. Swimming Behaviors

For Cladocera, 72.84 ± 6.81 nm PS reduced the swimming distances of *D. magna* in a dose-dependent manner within the range of 0–500 mg/L [53]. Both 20 and 200 nm PS-COOH significantly enhanced the *D. magna* swimming distance at 100 mg/L [113], and 1 and 10 μm PS increased *D. magna* swimming distance and speed at both 1.25 and 12.5 mg/L, which was considered to be avoidance behavior adopted by organisms to escape the polluted environment [81]. Plain PS and negative PS-${\mathrm{NH}}_{2}$ significantly reduced the swimming velocity and distance of *D. magna,* while PS-COOH and positive PS-${\mathrm{NH}}_{2}$ caused no significant effects [50]. PS co-existed with Gly induced greater effects on reducing *D. magna* swimming distance than single PS [53]. PS-COOH co-existed with Zn caused more effects on the frequency of movement of *D. magna* second antennae, thus affecting the swimming behavior [95]. PE reduced the *D. magna* hopping frequency in a dose-dependent manner within the range of 5–40 mg/L, which was related to the heartbeat rate reduction [83]. PE and BP-3 caused obvious damage to *D. magna* in terms of vertical swimming than single contaminants [114]. Polyamide (PA) could alleviate the decrease in velocity of *Gammarus roeseli* caused by phenanthrene (Phe), which might be related to the strong interaction between them [115]. For Decapoda, PS and PS-COOH affected the swimming speed, swimming time and swimming distance of *N. japonica* [63]. For Anostraca and Cirripedia, 0.1 µm PS beads significantly inhibited the *A. franciscana* and *A. amphitrite* swimming speeds at 1 and 10 mg/L [116], respectively.

### 3.3. Grazing and Defense Behaviors

For grazing behaviors, the PE spheres reduced the *D. magna* grazing rate on *Chlorella vulgaris* cells in a dose-dependent manner at 5, 20 and 40 mg/L, which was related to the hopping frequency reduction [83]. Dimethyl sulfide (DMS)-infused nylon fibers significantly reduced the *C. helgolandicus* grazing rates compared to virgin nylon fibers [117]. Additionally, for defense behaviors, the 4 mm PE at 25 particles/L significantly affected the attack and defense capabilities of *Pagurus bernhardus,* including effects on the attackers’ rapping intensity and strength, and weakening the defender’s ability to identify resources [118].

Microplastics exposure affects the feeding behaviors of aquatic crustaceans as these may be unable to distinguish similarly sized microplastics from food in most cases. When microplastics co-exist with contaminants such as oils and pesticides, they have a synergistic negative effect on the food intake of aquatic crustaceans. Primary microplastics reduce the feeding efficiency of aquatic crustaceans more than secondary microplastics. Many studies have shown that microplastics have negative impacts on the swimming behaviors of aquatic crustaceans, especially in terms of swimming speed and distance. The negative impact of the co-existence of microplastics and herbicides on the swimming behavior of aquatic crustaceans is higher than that of single contaminants. Microplastics reduced the grazing and defense behaviors of aquatic crustaceans.

## 4. Effects of Microplastics on Physiological Functions of Aquatic Crustaceans

There were 79 papers related to the physiological functions of microplastics on aquatic crustaceans, including oxidative damage (51 papers, 64.56%), neuromodulation (13 papers, 16.46%), energy regulation (13 papers, 16.46%), metabolic regulation (11 papers, 13.92%), respiratory regulation (7 papers, 8.86%), immunomodulation (7 papers, 8.86%) and intestinal physiology (8 papers, 10.13%) (Figure 2E). Among these, 31 papers focused on Decapoda (16 species), 26 papers on Cladocera (6 species), 11 papers on Anostraca (4 species), 7 papers on Copepoda (6 species), 3 papers on Amphipoda (4 species), 1 paper on Cirripedia (1 species) and 1 paper on Isopoda (1 species) (Figure 2F). The most concerned species among these aquatic crustaceans were *D. magna*, *A. franciscana*, *D. pulex* and *L. vannamei*. The most extensively utilized types of microplastics were PS (52 papers, 65.82%), PE (17 papers, 21.52%), PET (4 papers, 5.06%), PP (4 papers, 5.06%) and PVC (3 papers, 3.80%), whilst the most extensively utilized shapes were microspheres (33 papers, 41.77%), microbeads (19 papers, 24.05%), microfibers (5 papers, 6.33%) and fragments (4 papers, 5.06%).

### 4.1. Oxidative Damage

Most studies examined the oxidative stress caused by microplastics in aquatic crustaceans by measuring the lipid peroxidation (LPO), reactive oxygen species (ROS), total antioxidant capacity (TAC), $H_{2}O_{2}$ and antioxidant enzyme activities including catalase (CAT), glutathione S-transferase (GST), glutathione peroxidase (GPx), glutathione reductase (GR), glutathione (GSH), superoxide dismutase (SOD), malondialdehyde (MDA), etc. For Decapoda, 5 µm PS caused the activities of SOD, GSH and GPx in *Eriocheir sinensi*, first increasing and then decreasing with the PS concentration, which increased within the range of 0.04–40 mg/L and might be related to the activation of an antioxidant system at low concentrations and the weakening of antioxidant activity at high concentrations [119]. Moreover, 75 nm PS significantly decreased SOD, CAT, se-glutathione peroxidase (GSH-Px) and glutathione S-transferase (GSH-ST) activities as well as GSH contents, while the increased LPO content all fell within the range of 5–40 mg/L. Additionally, this significantly increased $H_{2}O_{2}$ and MDA contents in *M. nipponense* at 20 and 40 mg/L [62]. Between 10 and 22 µm PE clearly decreased the CAT activity in *L. vannamei* within the range of 0.1–1 μg/g in shrimp, increased the MDA content within the range of 0.2–1 μg/g in shrimp and decreased SOD activity at 0.2 and 1 μg/g in shrimp [66]. PS in the range of 0.5–1 µm and PE in the range of 30–150 µm significantly increased the antioxidant enzymes SOD, CAT, GST, GPx, GSH and LPO activities in *M. rosenbergii* in a dose-dependent manner at 1, 5 and 10 mg/100 g in food [87].

For Cladocera, PS raised the contents of total glutathione (T-GSH), GSH and oxidized glutathione (GSSG), as well as of ROS in *D. magna* within the ranges of 0–2 mg/L [120] and 0–500 mg/L [53] in a dose-dependent manner, respectively. Three-hundred nanometers of PS induced higher CAT and GPx activities in *D. magna* than the six-hundred-nanometer ones [121]. Additionally, 0.05 and 0.5 μm PS significantly increased the MDA concentration in *Diaphanosoma celebensis* while 6 μm PS caused moderate alterations [122]. Plain PS generated greater oxidative stress in *D. magna* than PS-COOH and PS-${\mathrm{NH}}_{2}$ [50], while amine-modified PS and carboxyl-modified PS both caused more severe oxidative damage to *D. magna* than PS [123]. PE significantly enhanced the ROS, TAC and LPO in *D. magna* in a dose-dependent manner within the range of 0.1–1 mg/L [55]. PE and BP-3 exhibited significantly synergistic interactions on the increasing ROS, TAC and LPO of *D. magna* [55]. Comparing PS and PE, 0.3–9 µm PE induced higher enzymes activities than 300 nm PS. Both PS (300 nm and 600 nm) and PE (0.3–9 µm) enhanced the oxidative stress of silver (Ag) by increasing the enzyme activity in *D. magna* [121].

For Anostraca, PS significantly increased the CAT activity in *A. franciscana* within the range of 0.001–1 mg/L [116]. PS-${\mathrm{NH}}_{2}$ significantly decreased the antioxidant enzyme GST and CAT activities in *A. franciscana* at 1 mg/L [91]. PS increased the ROS level in *A. salina* [124]. Additionally, PS-COOH could alleviate the oxidative damage in *A. salina* caused by nano-${\mathrm{TiO}}_{2}$ [73]. For Copepoda, 50 nm PS induced higher ROS in *T. japonicus* than 10 μm PS [26]. However, 0.05 μm PS significantly increased the ROS levels as well as GPx, GR, GST and SOD activities in *P. nana*, followed by 0.5 and 6 μm PS [89]. For Cirripedia, 0.1 µm PS significantly decreased the CAT activities in *A. amphitrite* at 0.001 mg/L and significantly increased it at 0.1 and 1 mg/L [116].

### 4.2. Neuromodulation

Neuromodulation could be measured by enzymes activities such as acetylcholineesterase (AChE), pseudocholinesterase (PChE), cholinesterase (ChE) and carboxylesterase (CbE). For Decapoda, PS could affect the neural activity by AChE activity reduction in *Charybdis japonica* [29]. Five-nanometer PS significantly reduced the AChE activity in *E. sinensis* in a dose-dependent manner within the range of 0–40 mg/L [119]. Additionally, PS could affect the changes in AChE activity caused by heavy metals (arsenic (As), Cd, Cu, Pb and Zn) in both *N. awatschensis* juveniles and adults [125]. For Anostraca, PS significantly decreased the AChE activity in *A. franciscana* at 0.001 and 0.01 mg/L and increased the PChE activity at 0.01 and 0.1 mg/L [116]. PS-${\mathrm{NH}}_{2}$ significantly reduced the ChE and CbE activities in *A. franciscana* at 1 mg/L [91]. Polymer spheres significantly increased the CbE and ChE activities in *A. franciscana* nauplii at 1.6 mg/L and significantly increased the CbE and ChE activities in *A. franciscana* juveniles at 0.4 and 1.6 mg/L [126]. For Cladocera, plain PS suspension increased the AChE activity, while the functionalized PS significantly decreased the AChE activity in *D. magna* [50]. One-micrometer PS affected AChE activity in *M. macrocopa* within the range of 0–500 μg/L after 7 days [127]. For Cirripedia, PS significantly increased the AChE and PChE activities in *A. amphitrite* within the range of 0.001–0.1 mg/L, especially at 0.001 mg/L [116].

### 4.3. Energy Regulation

The carbohydrate storage [128], fatty acid content and composition [129], essential amino acid content [130], carbon budget [131], energy balance [88], lipid stores [132] and protein content [133] could be used to evaluate the energy regulation. PS beads increased the energetic losses in *C. helgolandicus* [131], PS spheres caused changes in fatty acids and essential amino acids in *L. vannamei* [130] and PS fragments had more significant effects on the reduction in carbohydrate and protein energy storage on *D. magna* than beads [56]. Moreover, 300 μg/L PE more significantly reduced the ratios of protein, carbohydrates, lipids and caloric contents in *M. monogolica* than 100 μg/L PE [85], and mixtures of PE and Cd caused a more obvious energy drop than PE [85]. The PP fibers affected the energy balance in *C. maenas* [88] and reduced the stored lipids in *Nephrops norvegicus* [132]. PET significantly increased the energy consumption in *D. magna* when co-existed with Ag or Ag${\mathrm{NO}}_{3}$. Nylon fibers induced the higher lipid accumulation than nylon granules in *C. finmarchicus* [106].

### 4.4. Metabolic Regulation

The metabolic regulation of microplastics on aquatic crustaceans could be detected by metabolic enzymes activity (such as glutamic-oxaloacetic transaminase (GOT) and glutamic-pyruvic transaminase (GPT) [87]), excretion rates, metabolic rate [132], lipid metabolism [134], carbohydrate metabolism [128], digestive enzyme activity [135], etc. For Decapoda, 75 nm PS significantly affected the energy metabolism-related substance content and energy metabolism-related enzyme activity (glucose metabolism and lipid metabolism) in *M. nipponense* in dose-dependent manners within the range of 5–40 mg/L [60]. Two-hundred nanometer PS reduced the lipase (LPS) and acetyl-CoA carboxylase (ACC) activities in *Cherax quadricarinatus* at 0.5 and 5 mg/L [136], respectively. The PS co-existed with bisphenol A (BPA) more significantlyaffected the metabolism in *L. vannamei* than single contaminant [137]. PE and PS significantly reduced the metabolic enzymes of GOT and GPT activities in *M. rosenbergii* in a dose-dependent manner at 1, 5 and 10 mg/100 g in food [87].

### 4.5. Respiratory Regulation

The effects of microplastics on the respiration regulation in aquatic crustaceans could be detected by changes in respiration rate. For Copepoda, polystyrene reduced the respiration rates in *Centropages typicus* and *Acartia clausi* by 3.4 and 2.2 times, respectively [138]. Moreover, 6, 12 and 26 µm polystyrene could significantly reduce the *C. helgolandicus* respiration rate [139]. For Decapoda, PET fibers could decrease the respiration rates on later larval *Homarus americanus* [140]. PS could make *C. maenas* have a higher oxygen consumption [141]. PS co-existed with BPA caused more effects on *L. vannamei* respiration than single contaminant [137]. For Amphipoda, PMMA reduced the *Gammarus pulex* respiration rate at 0.52, 26.12 and 104.48 particles/${\mathrm{cm}}^{2}$ [142].

### 4.6. Immunomodulation

Immunomodulation could be tested by immune-related activities [62], immune-related genes expression [65] and the blood cell number [143]. For Decapoda, 75 nm PS induced reductions in the immune-related enzymes acid phosphatase (ACP), alkaline phosphatase (AKP), lysozyme (LZM) and phenoloxidase (PO) activities of *M. nipponense* within the range of 5–40 mg/L [62]. The 5 µm PS affected ACP, AKP, LZM and PO activities in the hemolymph and hepatopancreas of *E. sinensis* within the range of 0–40 mg/L, indicating that low-concentrations of PS might affect the immune response, and high concentrations of PS negatively influenced all aspects of innate immunity [144]. The 5 μm PE significantly increased the immune-related genes’ relative expression in *L. vannamei* at 500 and 5000 μg/L compared to 50 μg/L PE [65].

### 4.7. Intestinal Physiology

The intestinal physiology could be observed by detecting the intestinal microbiota of aquatic crustaceans. For Decapoda, PS induced the intestinal microbial imbalance in *E. sinensis* within the range of 0.04–40 mg/L [144]. PE induced the intestinal microbial imbalance in *L. vannamei* in a dose-dependent manner within the range of 50–5000 μg/L [65]. PE, PS, PP, PVC and polytetrafluoroethylene (PTFE) differently affected the stability of intestinal microbiota in *L. vannamei* and changed the gut microbiome in juveniles [134]. For Anostraca, PE has more serious disruption to the intestinal microbiota of *A. parthenogenetica* and a larger suppression of the growth rate compared with PS [92].

Most papers showed that microplastics could induce oxidative stress in aquatic crustaceans. In general, the oxidative stress of aquatic crustaceans caused by smaller microplastics is higher than that caused by larger microplastics. Microplastics co-existing with UV absorbers and other contaminants could cause synergistic effects on oxidative stress in aquatic crustaceans. Microplastics caused neurotoxicity in aquatic crustaceans. Microplastics affect the energy status of aquatic crustaceans, and the co-existence of microplastics and heavy metals has a greater impact on the energy consumption of aquatic crustaceans. Microplastics and other contaminants could cause synergistic effects on the metabolism and respiration of aquatic crustaceans. Microplastics exposure affected the stability of the intestinal microflora of aquatic crustaceans to varying degrees.

## 5. Response Mechanisms of Aquatic Crustaceans to Microplastics Stress

### 5.1. Oxidation Mechanism

Oxidative stress is one of the response mechanisms of aquatic crustaceans to microplastics stress. Oxidative stress frequently affects the cell membrane first, and transmits the stress signal to functional proteins via some signal transduction pathways such as mitogen-active protein kinases (MAPK), Jak-STAT, mTOR and Foxo, and then affects biological processes such as cell growth and proliferation. For example, PS permeated into the cell membrane with high bioavailability, which induced the cell damage, leading to the oxidative stress response of crustaceans. This is the main reason for the cell growth rate and proliferation inhibition in crustaceans exposed to PS [132]. PS induced oxidative stress in *E. sinensis* by affecting the MAPK signaling pathway [119]. The PS-caused ROS activates signaling in MAPK pathways (like p38 and p-JNK), resulting in lethal and adverse behavioral effects on *D. magna*. PS-COOH and negative PS-${\mathrm{NH}}_{2}$ might interact with cell surface receptor to activate MAPK by inducing p-p38 and p-JNK and stimulate the antioxidant system in the absence of ROS induction [50]. Additionally, for nanoplastics, nano-sized PS induced significant oxidative stress to aquatic crustaceans by penetrating the cellular membrane, causing more cellular damage than larger microplastics. These effects could be shown by the transcriptional modulation of antioxidant-related genes and enzyme activities [122]. Nano-sized PS induced *D. pulex* oxidative stress, affecting the arachidonic acid metabolism, glutathione metabolism and porphyrin and chlorophyll metabolism [145]. Nano-sized PS could induce a pronounced overproduction of ROS, leading to the inhibitions of *D. pulex* growth and reproduction through cell damage [146].

### 5.2. Metabolic Mechanism

The metabolic mechanism was another mechanism of aquatic crustaceans’ response to microplastics stress. Microplastics could affect the metabolism of aquatic crustaceans by affecting the adenosine monophosphate-activated protein kinase (AMPK) activation and then adenosine triphosphate (ATP) decomposition, affecting the metabolic mechanism and thus affecting various metabolic processes, energy status and other biological processes. In brief, the AMPK could be detected as activated, when the balance between ATP, adenosine diphosphate (ADP) and adenosine monophosphate (AMP) was destroyed. AMPK activation provided energy and helped organisms cope with stress through the synthesis of antioxidants and cellular stress responses [147]. Once activated by energy stressors, AMPK triggered metabolic changes by activating the catabolic pathways that generate ATP while shutting down anabolic pathways and other ATP-consuming processes that were not important for survival [148]. Microplastics could affect the various metabolic processes of aquatic crustaceans, such as lipid metabolism, glucose metabolism, etc. PS significantly reduced the expression of fatty acid metabolism-related genes (*FAD6* and *FABP*) in *C. quadricarinatus*, significantly increased the activity of lipase related to lipolysis in hepatopancreas, and increased that of fatty acid synthase related to fat synthesis and decreased that of acetyl-CoA carboxylase. The significant increased in lipid transport-related low-density lipoprotein suggests that lipolysis was superior to lipid synthesis [136]. PS could reduce the lipid content of juvenile shrimp by reducing the ability of *M. nipponense* to digest, transport and synthesize lipids. By down-regulating the expression of genes related to lipid metabolism, the PS reduced the activity of related enzymes, weakened the ability to digest, transport and synthesize lipids, resulting in the reduction in the lipid content in *M. nipponense* [60]. PS could cause glycolytic process inhibition, increase the anaerobic glucose metabolism, cause a shortage of energy supplies and lead to a shift from aerobic to anaerobic metabolism in *M. nipponense* [60]. Additionally, PS regulated the ion transport and energy metabolism-related enzymes and gene expression in *M. nipponense*, impacting the ion transport and damage on cell osmotic regulation [149]. PS led to an increase in several cellular biosynthetic processes, which in turn, due to trade-offs, reduced the energy storage, thus affecting the survival and reproduction of *T. japonicus* [99]. PS induced the disruption of the defense mechanism, as indicated by the abundance in ATP-binding cassette (ABC) family proteins, which can transfer harmful compounds out of cells [150].

### 5.3. Immune Mechanism

Microplastics also activate the immune mechanism of aquatic crustaceans. PS could activate the immune system of juvenile *M. nipponense*, stimulate the release of LZM from lysosomes and promote the release of hydrolytic enzymes AKP and ACP to resist immune stress, and high concentrations of PS or long-term exposure impaired lysosomal degradation and impeded phagocytosis by pathogens, inhibited the release of ACP and AKP in lysosomes and affected immune defense [62]. PS could stimulate cell apoptosis, which is an important regulatory feature of innate immune response and the most common form of death in immune cells, and might be through endocytosis across the cell membrane to activate the apoptosis pathway in *M. nipponense* [149]. PS impacted the contents and activities of most immune-related factors in hemolymph, blood cells, pancreas and liver of *E. sinensis* [144]. PS, PE, PP, PVC and PTFE differently affected the pathways and expression of immune-related proteins in *L. vannamei*. PS might influence shrimp’s antioxidant status and damage shrimp’s immunity in recognizing pathogenic molecules PE might also damage shrimp’s immunity by recognizing pathogenic molecules and affect the stability of shrimp’s extracellular matrix. PVC might influence shrimp’s immune homeostasis, antioxidant status and immunity in recognizing pathogenic molecules. PTFE induced an immune response [134].

## 6. Conclusions and Outlook

Most papers indicated that smaller microplastics tended to have greater effects on the life histories of aquatic crustaceans than those of larger microplastics in general. Irregular microplastics often had more negative impacts on the life histories of aquatic crustaceans than regular ones. Generally, microplastics co-existed with other contaminants had synergistic effects on the life history of aquatic crustaceans. Additionally, primary or pristine microplastics were found to have a greater negative impact on crustaceans’ survival than secondary or aged microplastics did. Microplastics caused negative effects on the feeding, swimming, grazing and defense behaviors of aquatic crustaceans. Microplastics co-existing with other contaminants led to more negative effects on feeding and swimming behaviors. Additionally, primary microplastics reduced the feeding efficiency of aquatic crustaceans more than secondary ones. Microplastics affected several physiological functions of aquatic crustaceans. Microplastics co-existed with other contaminants caused more negative effects on oxidative stress, metabolic regulation, metabolism and respiration in aquatic crustaceans. Smaller microplastics had more effects on the oxidative stress of aquatic crustaceans than larger microplastics. Oxidative stress frequently affected the cell membrane first, and transmitted the stress signal to functional proteins via some signal transduction pathways and then affected the biological processes such as cell growth and proliferation. Microplastics affected the metabolism of aquatic crustaceans, thus affecting organisms. Microplastics could also activate the immune mechanism of aquatic crustaceans differently and cause an immune response.

The review discovers that the research performed by studies on the effects of microplastics of aquatic crustaceans is incomplete and some problems need to be solved. Insufficient types of microplastics were used in the study. There were more studies on PS and PE, while there were less studies on other types of microplastics. There have been many studies on native microplastics, but few on aged or weathered microplastics. In this regard, we should expand the research on the types of microplastics, which should not only be limited to the available microplastics, but also pay more attention to microplastics affected by aging and weathering processes. It was found that the size ranges of microplastics are relatively limited and their shapes are relatively regular, in addition to being quite different from the shapes of the microplastics existing in the environment. In view of this problem, it is necessary to study the sizes and shapes of microplastics that are similar to those in the environment, and pay further attention to the impacts of irregularly shaped microplastics on aquatic crustaceans. It is also found that the test concentrations are generally higher than those in the environment. Therefore, we should strengthen the long-term impacts of microplastics with an environmental concentration on aquatic crustaceans. This review finds that the research on the impacts of microplastics co-existed with other contaminants in aquatic crustaceans is insufficient, and should be further strengthened. This review finds that the species of aquatic crustaceans used in studies are relatively limited, with more research focusing on Cladocera, especially *D. magna*. Therefore, we should pay more attention to the effects on more types of aquatic crustaceans, including different species and different life stages, and the exposure time should be extended to further observe the intergenerational effects and biohazards of microplastics. In addition, researchers should further research the mechanisms of biotoxicity in microplastics.

## Data Availability

Not applicable.

## Supplementary Materials

The following supporting information can be downloaded at: https://www.mdpi.com/article/10.3390/ijms24065523/s1.
