# Supplementary material for: Progress on the Effects of Microplastics on Aquatic Crustaceans: A Review
Source: Int J Mol Sci. 2023 Mar 14;24(6):5523. doi: 10.3390/ijms24065523 (PMC10052122; doi:10.3390/ijms24065523)
Supplement: Supplementary file 1 [file ijms-24-05523-s001.zip › ijms-2241391-supplementary.pdf]

Progress on the effects of microplastics on aquatic crustaceans: a review

Siyi Zhang, Haodi Wu and Jing Hou*

MOE Key Laboratory of Resources and Environmental Systems Optimization, College of Environmental Science and Engineering, North China Electric Power University, Beijing 102206, China

Correspondence: houjing@ncepu.edu.cn; Tel.: +86-10-6177-2864

Table S1. Effects of microplastics on life history of aquatic crustaceans. (✓ indicates affected, ○ indicates not involved, × indicates not affected significantly. "Not affected significantly" meant that the change of the experimental results was less than 10% or results of the referenced papers didn't show significance.).

Order/Subclass	Biological Species	Exposure Phase	MPs Type	Color	Shape	Size	Concentration	Type #	Concentration#	Exposure Time	Survival*	Growth and Development*	Reproduction*	Reference
Cladocera	*Moina monogolica*	neonate (12-24 h)	PE	-	particle	2-4 μm	100, 300 μg/L	Cd	0, 5, 10 μg/L	21 d	○	✓	✓	[1]
Cladocera	*Moina cf. micrura*	-	PET	-	irregular particle	<25 μm	940 ± 100 particles/mL	-	-	24, 48 h	✓	○	○	[2]
Cladocera	*Daphnia pulex, Moina macrocopa*	neonate (<24 h)	PS	nonfluorescent	sphere	0.7, 1 μm	2, 4, 8 mg/L	-	-	14 d	○	✓	✓	[3]
Cladocera	*Moina macrocopa*	neonate (<24 h)	PS	-	bead	1 μm	0.001-500 μg/L	-	-	14 d	✓	✓	✓	[4]
Cladocera	*Daphnia magna, Daphnia pulex, Ceriodaphnia dubia*	neonate (<24 h)	primary MPs<br>secondary PE	green fluorescent<br>-	sphere<br>irregular particle	1-5 μm<br>1-10 μm	$0\text{-}10^7$ particles/mL	-	-	96 h	✓	○	○	[5]
Cladocera	*Daphnia magna,*		primary MPs,	fluorescent	sphere MPs,			-	-	21 d	○	○	✓	[6]

Order/Subclass	Biological Species	Exposure Phase	MPs Type	Color	Shape	Size	Concentration	Type #	Concentration#	Exposure Time	Survival*	Growth and Development*	Reproduction*	Reference
	Daphnia pulex Ceriodaphnia dubia	neonate (<24 h)	secondary PE		irregular particle PE	1-5 μm, MPs, 1-10 μm PE	0-10^5 particles/mL			7 d				
Cladocera	*Ceriodaphnia dubia*	neonate (<24 h)	PE	white	bead	1-4 μm	0.5-16 mg/L	-	-	48 h	✓	○	○	[7]
			polyester	orange fluorescent	fiber	100-400 μm	0.125-4 mg/L							
							1/16-2 LC_{50}	PE beads	1/16-2 LC_{50}					
			PE	white	bead	1-4 μm	62.5-2000 μg/L	-	-	8 d	✓	✓	✓	
			polyester	orange fluorescent	fiber	100-400 μm	31.25-1000 μg/L							
Cladocera	*Ceriodaphnia dubia*	neonate (<24 h)	PS	-	bead	1 μm	15.6-500 mg/L	-	-	24 h	✓	○	○	[8]
							0.03-90 μg/L			7 d	○	○	✓	
Cladocera	*Ceriodaphnia dubia*	neonate (<24 h)	PS	-	bead	1 μm	0-8.5 mg/L	IMD<br>AC	0-7 mg/L<br>0-200 mg/L	24 h, 7 d	○	○	✓	[9]
Cladocera	*Diaphanosoma celebensis*	4 d	PS	unlabeled	sphere	0.05, 0.5, 6 μm	10-50 mg/L	-	-	48 h	✓	○	○	[10]

Order/Subclass	Biological Species	Exposure Phase	MPs Type	Color	Shape	Size	Concentration	Type #	Concentration#	Exposure Time	Survival*	Growth and Development*	Reproduction*	Reference
Cladocera	*Diaphanosoma celebensis*	-	PS	-	bead	0.05, 0.5, 6 µm	0.1, 1, 10 mg/L	-	-	14 d	○	○	✓	[11]
Cladocera	*Diaphanosoma celebensis*	4 d	PS	-	bead	0.05, 0.5, 6 µm	1 mg/L	Hg	0-60 µg/L	48 h	✓	○	○	[12]
Cladocera	*Daphnia carinata*	neonate (<24 h)	PE	clear	sphere	1–10 µm	0-300 mg/L	Cu	-	96 h	✓	○	○	[13]
							0.25, 1 mg/L		0, 1, 3 µg/L	21 d	✓	×	×	
Cladocera	*Daphnia pulex*	neonate (<24 h)	PS	-	sphere	71.18 nm	0.1-2 mg/L	-	-	21 d	○	○	✓	[14]
Cladocera	*Daphnia pulex*	neonate (<24 h)	PS	-	sphere	75 nm	1 µg/L	-	-	21 d	×	✓	✓	[15]
Cladocera	*Daphnia pulex*	neonate (<24 h)	PS	unlabeled	sphere	75 nm	1 mg/L	-	-	21 d	○	○	✓	[16]
Cladocera	*Daphnia pulex*	1, 4, 7, 14, 21 d	PS	unlabeled	sphere	75 nm	10-400 mg/L	-	-	48 h	✓	○	○	[17]
Cladocera	*Daphnia pulex*	neonate (<24 h)	PS	-	sphere	75 nm	10-400 mg/L	-	-	48 h	✓	○	○	[18]
							0.1-2 mg/L			21 d	○	✓	✓	

Order/Subclass	Biological Species	Exposure Phase	MPs Type	Color	Shape	Size	Concentration	Type#	Concentration#	Exposure Time	Survival*	Growth and Development*	Reproduction*	Reference
Cladocera	*Daphnia pulex*	neonate (<24 h)	PS	-	sphere	500 nm	0.5-4 mg/L	-	-	21 d	✓	✓	×	[19]
Cladocera	*Daphnia magna*	neonate (< 24 h)	PE<br>PS<br>PP<br>PVC	-	particle	50 nm<br>200, 600 nm<br>50 nm<br>200 nm	3×10^7 particles/mL	BaP	0, 10 μg/L	21 d	○	○	✓	[20]
Cladocera	*Daphnia magna*	-	HDPE	-	particle	<300 nm	-	-	-	98, 134 d	✓	○	✓	[21]
Cladocera	*Daphnia magna*	neonate	HDPE	clear	sphere	1 μm	12.5-400 mg/L	-	-	96 h	✓	○	○	[22]
Cladocera	*Daphnia magna*	-	PE	clear	sphere	1–4 μm	1, 10 mg/L	DM	0, 40 ng/L	21 d	✓	✓	✓	[23]
Cladocera	*Daphnia magna*	neonate	PE<br>PET/PA	clear<br>-	bead<br>fiber	1-10 μm<br>10×2 μm	2.2×10^4 mg/mL<br>0.1-0.3 mg/mL	Gly	2.5 mg/L	7 d	✓	○	○	[24]
Cladocera	*Daphnia magna*	neonate (<24 h)	secondary PE MPs	fluorescent	particle<br>bead	2.6 ± 1.8 μm<br>1–5 μm	10^2-10^5 particles/mL	-	-	21 d	✓	✓	✓	[25]
Cladocera	*Daphnia magna*	neonate (<24 h)	PE<br>PE/BP-3	-	fragment	16.68 ± 7.04 μm<br>17.35 ± 5.5 μm	4.35 mg/L<br>5 mg/L	-	-	21 d	×	✓	✓	[26]

Order/Subclass	Biological Species	Exposure Phase	MPs Type	Color	Shape	Size	Concentration	Type#	Concentration#	Exposure Time	Survival*	Growth and Development*	Reproduction*	Reference
Cladocera	*Daphnia magna*	4-5 d	PE	yellow	sphere	20, 30 μm	20, 60 mg/L	-	-	24 h	✓	○	○	[27]
Cladocera	*Daphnia magna*	neonate (2 d)	PE	clear	sphere	32–38 μm	5, 20, 40 mg/L	-	-	39 d	✓	✓	✓	[28]
Cladocera	*Daphnia magna*	juvenile (4 d)	PE	-	bead<br>fragment	37.05 ± 3.96 μm<br>37.24 ± 11.76 μm	0-20 mg/L	BP-3	0, 10 % w/w	48 h	✓	○	○	[29]
Cladocera	*Daphnia magna*	neonate (<24 h)	PE	-	particle	40-48 μm	20-320 mg/L	-	-	96 h	×	×	○	[30]
Cladocera	*Daphnia magna*	neonate (<24 h)	PE	white	particle	40-48 μm	20-320 mg/L	-	-	21 d	✓	✓	○	[31]
Cladocera	*Daphnia magna*	neonate (<24 h), juvenile (4 d)	PE	-	fragment	44.39 ± 11.16 μm	0-250 mg/L	BP-3	-	48 h	✓	○	○	[32]
		juvenile (4 d)				-<br>48.37 ± 6.26 μm	5 mg/L<br>4.5 mg/L		-<br>0.5 mg/L	17 d	✓	○	✓	
Cladocera	*Daphnia magna*	juvenile (4 d)	PE	-	fragment	17.23 ± 3.43 μm, 34.43 ± 13.09 μm	5 mg/L	-	-	21 d	✓	✓	✓	[33]

Order/Subclass	Biological Species	Exposure Phase	MPs Type	Color	Shape	Size	Concentration	Type #	Concentration#	Exposure Time	Survival*	Growth and Development*	Reproduction*	Reference
Cladocera	*Daphnia magna*	-	pristine PE, biofouled PE	-	bead<br>irregular fragment	40-48 μm<br>≤50 μm	10-200 mg/L	-	-	14 d	✓	○	○	[34]
Cladocera	*Daphnia magna*	7 d	PE	green fluorescent	bead	63-75 μm	25, 50, 100 mg/L	-	-	21 d	×	○	×	[35]
Cladocera	*Daphnia magna*	-	PE	pristine white<br>black	sphere<br>irregular fragment	10–106 μm<br>10-75 μm	0.0001-10 g/L	Phe	0-5 mg/L	24 h	✓	○	○	[36]
Cladocera	*Daphnia magna*	-	PE, PP, PVC	-	particle	10–100 μm	50 mg/L	-	-	96 h	✓	○	○	[37]
Cladocera	*Daphnia magna*	neonate	PS	yellow-green fluorescent	sphere	20 nm	1, 50 mg/L	-	-	F0 generation	×	×	✓	[38]
Cladocera	*Daphnia magna*	neonate (<24 h)	PS-COOH	fluorescent	particle	20, 200 nm	0.1-100 mg/L	-	-	48 h	✓	○	○	[39]

Order/Subclass	Biological Species	Exposure Phase	MPs Type	Color	Shape	Size	Concentration	Type #	Concentration#	Exposure Time	Survival*	Growth and Development*	Reproduction*	Reference
Cladocera	*Daphnia magna*	-	PS-COOH	-	sphere	26, 100 nm	0-100 mg/L	-	-	48 h	✓	○	○	[40]
Cladocera	*Daphnia magna*	neonate (<24 h)	PS	green fluorescent	sphere	50 nm	0.1-10 mg/L<br>0.05, 0.5 mg/L	-	-	48 h<br>21 d	✓	○	○	[41]
Cladocera	*Daphnia magna*	neonate (<24 h)	PS, PS-COOH, PS-NH_2	-	sphere	50-100 nm	-	-	-	24, 48 h	✓	○	○	[42]
Cladocera	*Daphnia magna*	adult	PS-NH_2	-	particle	52-330 nm	0.05-0.150 g/L	-	-	24 h	✓	○	○	[43]
Cladocera	*Daphnia magna*	2-5 d	PS-COOH	-	particle	26 nm	0.32, 3.2 mg/L	-	-	103 d	✓	○	○	[44]
						62 nm	0.32-7.6 mg/L							
			PS-NH_2			53 nm	0.0032, 0.032, 0.32 mg/L							
Cladocera	*Daphnia magna*	neonate (<24 h)	PS	-	bead	50, 500 nm	0-14.5 mg/L	-	-	48 h	✓	○	○	[45]
						5, 10, 15 µm	0-50 mg/L							
Cladocera	*Daphnia magna*	-	PS-NH_2	-	particle	50, 200, 500 nm	1.4, 2.7 mg/L	-	-	24, 48 h	✓	○	○	[46]

Order/Subclass	Biological Species	Exposure Phase	MPs Type	Color	Shape	Size	Concentration	Type #	Concentration#	Exposure Time	Survival*	Growth and Development*	Reproduction*	Reference
Cladocera	*Daphnia magna*	-	pristine PS, aged PS	Nile Red	sphere	~70 nm	0.22-150 mg/L.	-	-	21 d	○	✓	✓	[47]
Cladocera	*Daphnia magna*	neonate (<24 h)	PS	unlabeled	sphere	0.07, 1, 10 µm	1, 2 mg L	-	-	27 d	✓	○	✓	[48]
Cladocera	*Daphnia magna*	neonate (<24 h)	PS	-	sphere	72.84 ± 6.81 nm	15.6-500 mg/L	Gly	0-200 mg/L	48 h	✓	○	○	[49]
							1.65-13.24 mg/L		1.25-9 mg/L	21 d F0 generation	○	○	✓	
Cladocera	*Daphnia magna*	neonate (<24 h)	PS	-	sphere	100 nm	0-75 mg/L	-	-	24, 48 h	✓	○	○	[50]
			PS-COOH			300 nm	0-70 mg/L							
			negative PS-NH_2			50–100 nm	0-40 mg/L							
			positive PS-NH_2			110 nm	0-100 mg/L							
Cladocera	*Daphnia magna*	-	PS	fluorescent	sphere	100 nm, 2 µm	0.1, 0.5, 1 mg/L	-	-	21 d	×	×	✓	[51]
Cladocera	*Daphnia magna*	neonate	PS-COOH, PS-NH_2	fluorescent	particle	nanometer size	0.1 mg/L-1 g/L	-	-	24 h	✓	○	○	[52]

Order/Subclass	Biological Species	Exposure Phase	MPs Type	Color	Shape	Size	Concentration	Type#	Concentration#	Exposure Time	Survival*	Growth and Development*	Reproduction*	Reference
Cladocera	*Daphnia magna*	-	PS	-	particle	0.1 μm	1 mg/L	ZnO	5 mg/L	8 h	✓	○	○	[53]
Cladocera	*Daphnia magna*	adult	PS	-	sphere	0.1, 1, 10 μm	1 mg/L	Ag	50 μg/L	8 h	✓	○	○	[54]
Cladocera	*Daphnia magna*	neonate	PS	fluorescent	sphere	100 ± 10 nm	10–100 mg/L	-	-	24 h	✓	○	○	[55]
Cladocera	*Daphnia magna*	neonate (<24 h)	pristine PS, aged PS	-	particle	0.1~50 μm	0.1-50 mg/L	APFO	-	48 h	✓	○	○	[56]
							0-46.5 mg/L		0-200 mg/L	24, 48 h	✓	○	○	
Cladocera	*Daphnia magna*	neonate (<24 h)	$PS-NH_2$	unlabelled	sphere	0.1-0.12 μm, 1-1.3 μm	1-400 mg/L	HA	-	96 h	✓	○	○	[57]
						0.1-0.12 μm	200 mg/L		0, 5 mg/L					
Cladocera	*Daphnia magna*	neonate	$PS-NH_2$	-	sphere	0.10-0.12 μm	1-400 mg/L	NOM FA HA	10 mg/L	96 h	✓	○	○	[58]
Cladocera	*Daphnia magna*	-	PS	-	particle	154.1 ± 2.9 nm	5 μg/L	-	-	31 d	○	✓	✓	[59]
Cladocera	*Daphnia magna*	neonate (<24 h)	PS PS-COOH	-	sphere	201.5 nm 191.3 nm	1-30 mg/L	Ni	1-5 mg/L.	48 h	✓	○	○	[60]
Cladocera	*Daphnia magna*	neonate	amidine PS,	-	sphere	200 nm	10-400 mg/L	HSs, polysaccha	1-5 mg/L	48 h	✓	○	○	[61]

Order/Subclass	Biological Species	Exposure Phase	MPs Type	Color	Shape	Size	Concentration	Type #	Concentration#	Exposure Time	Survival*	Growth and Development*	Reproduction*	Reference
			carboxyl PS					ride chains						
Cladocera	*Daphnia magna*	neonate (<24 h)	PS, PS-COOH, PS-NH_2	-	particle	220 nm	20, 30 mg/L	HA	0-50 mg/L	48 h	✓	○	○	[62]
Cladocera	*Daphnia magna*	adult	PS<br>PE	-	sphere	300, 600 nm<br>0.3-9 μm	6.74×10^7 particles/mL	Ag, DOM	0-10 μg/L Ag, 0-50 mg/L DOM	72 h	✓	○	○	[63]
Cladocera	*Daphnia magna*	neonate	PS-COOH	fluorescent	sphere	500 nm	1 mg/L	-	-	till produce second clutch	○	✓	✓	[64]
Cladocera	*Daphnia magna*	-	PS-COOH	yellow-green fluorescent	particle	0.5 μm	0.01-0.1 mg/L	-	-	7 d	×	✓	✓	[65]
Cladocera	*Daphnia magna*	-	PS	yellow-green fluorescent	sphere	1 μm	10^{-3} mg/L	Cr (VI)	-<br>2, 5 mg/L	72 h	×<br>✓	○<br>○	○<br>○	[66]

Order/Subclass	Biological Species	Exposure Phase	MPs Type	Color	Shape	Size	Concentration	Type#	Concentration#	Exposure Time	Survival*	Growth and Development*	Reproduction*	Reference
Cladocera	*Daphnia magna*	neonate (<24 h)	PS	-	bead	1 μm	1, 10, 100 particles/L	Cr (VI)	0–200 μg/L<br>0-10 μg/L	48 h<br>24 d	✓<br>○	○<br>○	○<br>✓	[67]
Cladocera	*Daphnia magna*	neonate (<24 h)	PS	fluorescent	sphere	1 μm	0.29 mg/L	dimethoate<br>DM	0-5 mg/L<br>0-10 μg/L	72 h	×	○	○	[68]
Cladocera	*Daphnia magna*	neonate (<24 h)	PS	pristine, fluorescent	sphere	1 μm	0.5-7.5 mg/L	MB	0, 0.25 mg/L	10 h	✓	○	○	[69]
Cladocera	*Daphnia magna*	-	PS	green fluorescent	bead	1-5 μm	0-10^5 particles/mL	-	-	21 d	○	○	✓	[70]
Cladocera	*Daphnia magna*	neonate (<24 h), adult	PS	red	bead	1, 10 μm	0.125, 1.25, 12.5 mg/L	-	-	21 d	○	✓	✓	[71]
Cladocera	*Daphnia magna*	neonate (<24 h)	PS	-	bead	1 μm<br>10 μm	0.1-600 mg/L<br>0.005-40 mg/L	-	-	48 h	✓	○	○	[72]
Cladocera	*Daphnia magna*	-	PS	unlabeled	sphere	1.25 μm	0.5, 1, 2 mg/L	-	-	21 d	✓	○	✓	[73]

Order/Subclass	Biological Species	Exposure Phase	MPs Type	Color	Shape	Size	Concentration	Type #	Concentration#	Exposure Time	Survival*	Growth and Development*	Reproduction*	Reference
Cladocera	*Daphnia magna*	-	aged PS	-	particle	0–1.5, 10–60, 60–230 μm	1-30 mg/L	-	-	48 h	✓	○	○	[74]
Cladocera	*Daphnia magna*	neonate (<24 h), 18 d	PS-COOH	nonfluorescent	particle	2 μm	1.39, 11.1 μg/L	-	-	21 d	✓	✓	✓	[75]
Cladocera	*Daphnia magna*	neonate, adults	PS-COOH	red fluorescent	bead	2 μm	1, 10 mg/L<br>1 mg/L	Zn	0-10 mg/L<br>0-2.5 mg/L	48, 96 h<br>21 d	✓<br>✓	✓<br>✓	○<br>✓	[76]
Cladocera	*Daphnia magna*	-	secondary MPs (PS, PET, ABS)	-	particle	3.2-3.7 μm	1 % of diet	-	-	14 d	✓	✓	✓	[77]
Cladocera	*Daphnia magna*	neonate (<24 h), juvenile (<96 h)	PS<br>aged PS	-	particle	<5 μm	~2×10^{5} particles/mL<br>~3.3×10^{5} particles/mL	-	-	24 h, 21 d	✓	✓	✓	[78]
Cladocera	*Daphnia magna*	neonate (<24 h)	aged PS	green fluorescent	sphere	5 μm	2.5 μg/L	ROX	0, 0.1, 10 μg/L	21 d	✓	○	✓	[79]

Order/Subclass	Biological Species	Exposure Phase	MPs Type	Color	Shape	Size	Concentration	Type#	Concentration#	Exposure Time	Survival*	Growth and Development*	Reproduction*	Reference
Cladocera	*Daphnia magna*	neonate (6-24 h)	PS	-	particle	5.8 μm	5-200 mg/L	TCS, TCC, MTCS	-	48 h	✓	×	○	[80]
							1 mg/L		0-272μg/L		✓	×	○	
							0.5, 1, 2 mg/L		-	21 d	○	✓	✓	
									0-4 μg/L		○	✓	✓	
Cladocera	*Daphnia magna*	neonate (<24 h)	PS	fluorescent	sphere	6 μm	5-300 mg/L	-	-	120 h	✓	○	○	[81]
		adult (9 d)									✓	○	○	
		neonate (<24 h)					5-100 mg/L			77 d	✓	✓	✓	
		adult (9 d)								80 d	✓	✓	✓	
Cladocera	*Daphnia magna*	neonate (<24 h)	PS	-	sphere	6, 20 μm	500, 5000 particles/mL	-	-	21 d	○	✓	✓	[82]
					irregular fragment	5.7, 18 μm								
					fiber	3×75 μm								
Cladocera	*Daphnia magna*	juvenile (1 w)	PS	-	sphere	10, 50 μm	0-1000 mg/L	Pb, Cu, Cd, Ni	-	48 h	✓	○	○	[83]
									0-1.846 mg/L		✓	○	○	

Order/Subclass	Biological Species	Exposure Phase	MPs Type	Color	Shape	Size	Concentration	Type#	Concentration#	Exposure Time	Survival*	Growth and Development*	Reproduction*	Reference
Cladocera	*Daphnia magna*	neonate (<24 h)	PS	-	particle	13.03 ± 7.75 μm	101.6 mg/L	-	-	19 d	○	✓	✓	[84]
Cladocera	*Daphnia magna*	neonate<br>adult	PS-COOH	green fluorescent	particle	15 μm	100, 800 particles/mL	-	-	21 d	×<br>×	✓<br>○	×<br>×	[85]
Cladocera	*Daphnia magna*	10 d	PS	-	sphere	~21.6 μm	25%, 75% in ratio of PS to food	-	-	7 d	✓	✓	○	[86]
Cladocera	*Daphnia magna*	-	PS	-	sphere	<30 μm	16 μl/L	-	-	96 h	✓	✓	○	[87]
Cladocera	*Daphnia magna*	juvenile, adult	MPs mixtures	fluorescent	irregular particle	-40 μm	1 % MPs in food	-	-	20-22 d	×	×	×	[88]
Cladocera	*Daphnia magna*	neonate (<24 h)	pristine PS, aged PS	-	irregular particle	≤63 μm	80-10000 particles/mL	-	-	21 d	✓	✓	✓	[89]
Cladocera	*Daphnia magna*	neonate (<24 h)	secondary PS	-	irregular particle	<63 μm	400-10000 particles/mL	-	-	21 d	✓	✓	✓	[90]
Cladocera	*Daphnia magna*	-	PET, PC, PBT	-	irregular particle	<50 μm	5-100 mg/L	-	-	72 h<br>21 d	✓<br>✓	✓<br>✓	○<br>○	[91]
Cladocera	*Daphnia magna*	neonate (<24 h)	PVC, PUR, PLA	-	particle	≤59 μm	10-500 mg/L	-	-	21 d	✓	✓	✓	[92]

Order/Subclass	Biological Species	Exposure Phase	MPs Type	Color	Shape	Size	Concentration	Type#	Concentration#	Exposure Time	Survival*	Growth and Development*	Reproduction*	Reference
Cladocera	*Daphnia magna*	neonate	degradative PP	-	particle	< 52 μm	19 particles/L	-	-	24, 48 h	×	○	○	[93]
			degradative PVC	-		< 28 μm					✓	○	○	
Cladocera	*Daphnia magna*	72-h	PP	red fluorescent	fiber	134.83 ± 88.70 μm	1000, 2000 mg/L	-	-	48 h	✓	○	○	[94]
			PET			120.49 ± 82.46 μm								
Cladocera	*Daphnia magna*	neonate (<24 h)	PVC	-	-	50 ± 10 μm	10-160 mg/L	-	-	48 h	✓	○	○	[95]
						2 ± 1 μm	2.05 mg/L			21 d	○	✓	✓	
						50 ± 10 μm	4.97 mg/L							
Cladocera	*Daphnia magna*	neonate (<24 h)	PVC	-	particle	<150 μm	0-0.15 mg/L	-	-	31 d	×	×	✓	[96]
Cladocera	*Daphnia magna*	neonate	PET	-	fiber	360×14 μm	20-500 mg/L	Ag, $AgNO_3$	-	96 h	×	○	○	[97]
		5 d					100 mg/L		0.1-10 μg Ag/L	48 h	×	○	○	
Cladocera	*Daphnia magna*	-	PET	-	fiber	60-1400×30-530×2-21.5 μm	12.5, 25, 50, 100 mg/L	-	-	48 h	✓	×	○	[98]

Order/Subclass	Biological Species	Exposure Phase	MPs Type	Color	Shape	Size	Concentration	Type#	Concentration#	Exposure Time	Survival*	Growth and Development*	Reproduction*	Reference
Cladocera	*Daphnia magna*	-	EAA	-	particle	103 nm	1.2 mg/L	-	-	21 d	×	×	×	[99]
Cladocera	*Daphnia magna*	-	polymer	red fluorescent	sphere	1–5 μm	0.1 mg/L	-	-	21 d	✓	○	✓	[100]
Cladocera	*Daphnia magna*	juvenile (6-24 h)	polymer	red fluorescent	sphere	1–5 μm	0.04, 0.09, 0.19 mg/L	-	-	21 d	○	✓	✓	[101]
Cladocera	*Daphnia magna*	juvenile (6-24 h)	polymer	fluorescent	sphere	1–5 μm	0.05, 0.1, 0.2 mg/L	Li	0.02, 0.04, 0.08 mg/L	21 d	✓	✓	✓	[102]
Cladocera	*Daphnia magna*	-	polymer	green fluorescent	sphere	1.31–5 μm	7 ×10^5 particles/mL	-	-	96 h	×	×	×	[103]
Cladocera	*Daphnia magna*	neonates (<24 h)	PMMA, PMMA-PSMA	fluorescent	-	86-125 nm	0.01-1000 mg/L	-	-	48 h	✓	○	○	[104]
Cladocera	*Daphnia magna*	juvenile (6-24 h)	MPs	fluorescent	sphere	1–5 μm	0.02, 0.2 mg/L	Au	0, 0.2, 2 mg/L	21 d	✓	✓	✓	[105]
Cladocera	*Daphnia magna*	neonates	PMA	-	particle	1.5-10 μm.	25-150 mg/L<br>1-30 mg/L	-	-	96 h<br>21 d	✓<br>✓	✓<br>✓	○<br>✓	[106]

Order/Subclass	Biological Species	Exposure Phase	MPs Type	Color	Shape	Size	Concentration	Type#	Concentration#	Exposure Time	Survival*	Growth and Development*	Reproduction*	Reference
		(<24 h)	PVA	-	film	-	25-125 mg/L			96 h	✓	✓	○	
							2.5-50 mg/L			21 d	✓	✓	✓	
Cladocera	*Daphnia magna*	neonates (<24 h)	PA, UV-weathered PA	-	particle	< 180 μm	100, 300 mg/L	-	-	21 d	×	○	×	[107]
Cladocera	*Daphnia magna*	-	polyester	-	fiber	26–5761 μm	0.15-150 mg/L	-	-	48 h, 21 d	✓	○	✓	[108]
Cladocera	*Daphnia magna*	neonates (<24 h)	tire particles	-	particle	<1 μm, 1-20 μm	8.18–54.05 mg/L	-	-	48 h	✓	○	○	[109]
Cladocera	*Daphnia magna*	neonate	MPs	-	particle	20-250 μm	100 mg/L	-	-	48 h	×	✓	○	[110]
			PET		fiber	20×100-500 μm								
Cladocera	*Daphnia magna*	24 h	MPs	-	particle	180.5 ± 118.7 μm	10, 100 mg/L	Ag	-	48 h	×	○	○	[111]
			aged MPs			303.5 ± 134.1 μm								
Decapoda	decapod	-	aged PS	fluorescent	sphere	15 μm	50-200 particles/mL	-	-	3, 7, 11 d	×	○	○	[112]
			PS			15, 30 μm								
					fragment	<30 μm								

Order/Subclass	Biological Species	Exposure Phase	MPs Type	Color	Shape	Size	Concentration	Type#	Concentration#	Exposure Time	Survival*	Growth and Development*	Reproduction*	Reference
Decapoda	American lobster (*Homarus americanus*)	larva	PET	neon pink	fiber	459 ± 2.25 μm	1, 10, 25 fibers/mL	-	-	10 d	✓	✓	○	[113]
Decapoda	Antarctic krill (*Euphausia superba*)	juvenile	PS-COOH<br>PS-NH_2	unlabeled<br>-	particle	62 nm<br>50 nm	2.5 mg/L	-	-	48 h	×	✓	○	[114]
Decapoda	Antarctic krill (*Euphausia superba*)	embryo	PS-NH_2	-	sphere	160 nm	2.5 mg/L	-	-	6 d	○	✓	○	[115]
Decapoda	Antarctic krill (*Euphausia superba*)	adult	PE	green fluorescent	sphere	27-32 μm	201-1603 μg/L	-	-	10 d	×	×	○	[116]
Decapoda	shrimp (*Penaeus monodon, Marsupenaeus japonicas, Litopenaeus vannamei*)	juvenile	PE	-	bead	5 μm	25-300 mg/L	-	-	48 h	✓	○	○	[117]
Decapoda	white-leg shrimp	juvenile	PE	red fluorescent	particle	5 μm	50, 500, 5000 μg/L	-	-	48 h	✓	○	○	[118]

Order/Subclass	Biological Species	Exposure Phase	MPs Type	Color	Shape	Size	Concentration	Type#	Concentration#	Exposure Time	Survival*	Growth and Development*	Reproduction*	Reference
	(*Litopenaeus vannamei*)													
Decapoda	*Litopenaeus vannamei*	juvenile	PE	red fluorescent	sphere	10-22 μm	0.1-1 μg/g shrimp	-	-	7 d	✓	○	○	[119]
Decapoda	whiteleg shrimp (*Litopenaeus vannamei*)	-	PS	blue	sphere	44 nm	50 mg/L	-	-	21 d	○	×	○	[120]
Decapoda	Pacific white shrimp (*Litopenaeus vannamei*)	-	PS	-	sphere	100 nm	200, 2000 mg/kg	TiO_2	200, 2000 mg/kg	28 d	○	✓	○	[121]
Decapoda	whiteleg shrimp (*Penaeus vannamei*)	-	weathered PE	-	particle	32-43 μm	1-5 μg/L	-	-	25 d	○	✓	○	[122]
Decapoda	daggerblade grass shrimp (*Palaemonetes pugio*)	adult	PE<br>PS<br>PP	green fluorescent<br>opaque<br>white<br>black	sphere<br>fragment<br>fiber	35-165 μm<br>30, 75 μm<br>34, 93 μm	50 particles/mL	-	-	3 h	✓	○	○	[123]

Order/Subclass	Biological Species	Exposure Phase	MPs Type	Color	Shape	Size	Concentration	Type #	Concentration#	Exposure Time	Survival*	Growth and Development*	Reproduction*	Reference
Decapoda	grass shrimp (*Palaemonetes pugio*)	-	PE	green fluorescent	sphere	53-180 µm	50 particles/mL	-	-	96 h	×	○	○	[124]
			PP	white	fragment	40 µm								
			polyester	-	fiber	63-150 µm								
Decapoda	grass shrimp (*Palaemon pugio*)	-	PE	green fluorescent	bead	32–38, 53–63 µm	62.5, 625, 6250 particles/L	-	-	23 d	✓	✓	○	[125]
Decapoda	mysid (*Neomysis awatschensis*)	juvenile, adult	PS	-	bead	1 µm	10 particles/mL	As, Cd, Cu, Pb, Zn	20–100 mg/L	96 h	✓	○	○	[126]
Decapoda	marine mysid (*Neomysis awatschensis*)	juvenile, adult	PS	fluorescent	bead	1, 10 µm	1×10^3–5×10^5 particles/mL	-	-	40 d	✓	✓	○	[127]
Decapoda	mysid shrimp (*Neomysis japonica*)	larva	PS, PS–COOH	fluorescent	bead	5 µm	10-6250 µg/L	-	-	96 h	✓	✓	○	[128]

Order/Subclass	Biological Species	Exposure Phase	MPs Type	Color	Shape	Size	Concentration	Type#	Concentration#	Exposure Time	Survival*	Growth and Development*	Reproduction*	Reference
Decapoda	shrimp (*Macrobrachium nipponense*)	juvenile	PS	-	sphere	75 nm	0-606 mg/L	-	-	96 h	✓	✓	○	[129]
Decapoda	oriental river prawn (*Macrobrachium nipponense*)	juvenile	PS	-	sphere	75 nm	5-40 mg/L	-	-	28 d	✓	✓	○	[130]
Decapoda	prawn (*Macrobrachium nipponense*)	adult	PS	-	sphere	0.5 μm	0.04-40 mg/L	-	-	21 d	×	✓	○	[131]
Decapoda	oriental river prawn (*Macrobrachium nipponense*)	larvae	PS	red fluorescent	sphere	5 μm	40-1280 mg/L	-	-	96 h	✓	○	○	[132]
							2, 20 mg/L			28 d	✓	✓	✓	
Decapoda	giant river prawn (*Macrobrachium rosenbergii*)	juvenile	PS	-	sphere	0.5–1 μm	1, 5, 10 mg/100 g in food	-	-	60 d	✓	✓	○	[133]
			PE		irregular flake	30-150 μm								
Decapoda	fiddler crab (*Minuca ecuadoriensis*)	adult	HDPE	-	particle	<250 μm	200 mg/L	MLT	0, 50 mg/L	5 d	✓	○	○	[134]

Order/Subclass	Biological Species	Exposure Phase	MPs Type	Color	Shape	Size	Concentration	Type#	Concentration#	Exposure Time	Survival*	Growth and Development*	Reproduction*	Reference
Decapoda	Chinese mitten crab (*Eriocheir sinensis*)	juvenile	PS	virgin	sphere	5 μm	0.04-40 mg/L	-	-	21 d	×	✓	○	[135]
Decapoda	crab (*Carcinus maenas*)	-	PP	blue	fiber	500 μm	0.3%, 0.6%, 1% in food	-	-	28 d	○	✓	○	[136]
Decapoda	mud crab (*Rhithropanopeus harrisii*)		PP	red	fiber	0.03×0.2-0.5 mm	-	-	-	28 d	○	×	○	[137]
			PET	-	fragment	<0.25 mm								
			MPs		bead	0.2-1 mm								
Decapoda	Norway lobster (*Nephrops norvegicus*)	-	PS	colorless	sphere	6 μm, 500-600 μm	-	PCBs	loaded on MPs	21 d	×	×	○	[138]
			PE			500-600 μm								
Decapoda	langoustine (*Nephrops norvegicus*)	-	PP	-	fiber	3-5×0.2 mm	-	-	-	8 months	○	✓	○	[139]
Decapoda	redclaw crayfish (*Cherax quadricarinatus*)	juvenile	PS	fluorescent	sphere	200 nm	0.5, 5 mg/L	-	-	21 d	×	✓	○	[140]

Order/Subclass	Biological Species	Exposure Phase	MPs Type	Color	Shape	Size	Concentration	Type#	Concentration#	Exposure Time	Survival*	Growth and Development*	Reproduction*	Reference
Copepoda	*Pseudodiaptomus annandalei*	-	PS	-	particle	0.1 µm	-	-	-	15 d	✓	○	✓	[141]
Copepoda	*Acartia clausi*	adult female	polystrene	red fluorescent	bead	6 µm	5000 beads/mL	-	-	5-8 d	✓	○	○	[142]
Copepoda	*Tigriopus japonicus*	nauplius (<24 h)	PS	fluorescent	bead	0.05, 0.5, 6 µm	0-313 mg/L	-	-	96 h	✓	○	○	[143]
(cont.)							0.125-25 mg/L			14 d	✓	✓	✓	
Copepoda	*Tigriopus japonicus*	-	PS	fluorescent	bead	50 nm, 2 µm	0.5 µg/L, 0.1-100 mg/L	-	-	30 d	✓	✓	○	[144]
(cont.)										40 d	○	○	✓	
Copepoda	*Tigriopus japonicus*	adult	PS	-	bead	50 nm, 10 µm	20 mg/L	-	-	24, 48 h	×	×	○	[145]
Copepoda	*Tigriopus japonicus*	adult	PS		sphere	0.1, 0.55, 5 µm	20-500 mg/L		-	96 h	×	○	○	[146]
(cont.)						0.1 µm	1, 20 mg/L	DBP	0-8 mg/L					
(cont.)							1, 10 mg/L		0, 0.1, 0.5 mg/L	20 d	×	○	✓	
Copepoda	*Tigriopus japonicus*	nauplii (<24 h)	PS	fluorescent	bead	6 µm	0.023, 0.23 mg/L	-	-	2 generations	✓	○	✓	[147]
Copepoda	*Tigriopus japonicus*	-	PS	fluorescent	sphere	10 µm	2, 20, 200 µg/L	Cd	15.2 µg/L	24 d	○	✓	✓	[148]
		adult -	PE	-	particle	10-30 µm	12.5 mg/L	-	-	14 d	✓	✓	✓	

Order/Subclass	Biological Species	Exposure Phase	MPs Type	Color	Shape	Size	Concentration	Type#	Concentration#	Exposure Time	Survival*	Growth and Development*	Reproduction*	Reference
Copepoda	*Tigriopus japonicus*		PA 6			5-20 μm								[149]
Copepoda	*Tigriopus japonicus*	-	tire wear particles	-	particle	6-130 μm	320-2560 mg/L	-	-	96 h	✓	○	○	[150]
Copepoda	*Tigriopus japonicus*	nauplius (<24 h)	MPs	-	particle	-	1, 10, 100 particles/mL	-	-	until fertilization	○	✓	✓	[151]
Copepoda	*Tigriopus japonicus*	-	secondary MPs	Nile Red	particle	10-40 μm	20, 500 mg/L	TCS	-	48 h	×	○	○	[152]
							20 mg/L		90.5-732.08 μg/L		✓	○	○	
										48 h	✓	○	○	
Copepoda	*Tigriopus fulvus*	nauplius (<24 h)	PP	blue, green, pink, white, yellow	particle	<1.2μm	0-150 mg/L	-	-	5-9 d	○	✓	○	[153]
Copepoda	*Nitokra lacustris pacifca*	-	PS, biodegradable PHA	Nile red	bead	6.58 μm PS, 7-140 μm PHA	350, 700 beads/mL	-	-	48 h	✓	✓	✓	[154]
										till spawning or	○	○	✓	

Order/Subclass	Biological Species	Exposure Phase	MPs Type	Color	Shape	Size	Concentration	Type#	Concentration#	Exposure Time	Survival*	Growth and Development*	Reproduction*	Reference
										adulthood				
Copepoda	*Parvocalanus crassirostris*	nauplius	PET	-	particle	5–10 μm	20000 particles/mL	-	-	6, 24 d	✓	○	✓	[155]
		adult					10000-80000 particles/mL			5 d	○	○	✓	
Copepoda	*Paracyclopina nana*	nauplius	PS	-	bead	0.05, 0.5, 6 μm	0.1-20 mg/L	-	-	until mature	○	✓	✓	[156]
Copepoda	*Acartia tonsa*	-	HDPE	-	particle	2-10 μm	0.1-100 μg/L	CPF	-	48 h	×	○	×	[157]
									0.1-100 μg/L		✓	○	✓	
Copepoda	*Acartia tonsa*	-	PE	white	bead	10–90 μm	0–25000 beads/mL	TCS	-	48 h	×	○	○	[158]
							500 beads/mL		0–300 μg/L		✓	○	○	
Copepoda	*Acartia tonsa*	nauplius	PS	red	particle	6-8 μm	1197 particles/mL	-	-	5 d	✓	✓	○	[159]
		juvenile								7 d	✓	○	✓	
Copepoda	*Acartia tonsa,*	-	tire wear MPs	-	particle	8-20 μm	0.01–10 particles/mL	-	-	24 h	○	○	×	[160]

Order/Subclass	Biological Species	Exposure Phase	MPs Type	Color	Shape	Size	Concentration	Type#	Concentration#	Exposure Time	Survival*	Growth and Development*	Reproduction*	Reference
Copepoda	*Temora longicornis*<br>*Acartia tonsa*<br>*Calanus finmarchicus*	-	HDPE, PS	-	sphere	3-16, 90-106 μm HDPE, 10 μm PS	-	PAH (Phe, Fla)	20 μg/L	96 h<br>48 h	✓	○	○	[161]
Copepoda	*Calanus finmarchicus*	juvenile	nylon	-	fiber<br>granule	10×30 μm<br>10-30 μm	50 plastics/mL	-	-	6 d	○	✓	○	[162]
Copepoda	*Calanus finmarchicus*, *Calanus glacialis*, *Calanus hyperboreus*	-	PE	clear	sphere	20.7 μm	0.2, 20 particles/mL	-	-	6 d	○	○	✓	[163]
Copepoda	*Calanus finmarchicus*, *Acartia longiremis*, *Pseudocalanus* spp.	-	aged PS<br>PS	fluorescent	sphere<br>fragment	15 μm<br>15, 30 μm<br><30 μm	50-200 particles/mL	-	-	3, 7, 11 d	×	○	○	[112]
Copepoda	*Calanus helgolandicus*	-	PS	-	bead	20 μm	65 beads/mL	-	-	9 d	×	○	✓	[164]
Anostraca	brine shrimp	-	PS	-	particle	40-50 nm	1, 10, 100 mg/L	HA	0, 5 mg/L	48 h	✓	○	○	[165]

Order/Subclass	Biological Species	Exposure Phase	MPs Type	Color	Shape	Size	Concentration	Type #	Concentration#	Exposure Time	Survival*	Growth and Development*	Reproduction*	Reference
	(*Artemia franciscana*)													
Anostraca	brine shrimp (*Artemia franciscana*)	nauplius	PS-NH_2	-	sphere	50 nm	0.1-10 mg/L	-	-	48 h<br>14 d	○<br>✓	✓<br>✓	○<br>○	[166]
Anostraca	brine shrimp (*Artemia franciscana*)	Instar I larva	PS	blue	bead	0.1 μm	0.001-10 mg/L	-	-	24, 48 h	×	○	○	[167]
Anostraca	brine shrimp (*Artemia franciscana*)	nauplius	PS-NH_2	-	partcle	190 nm	0-200 mg/L	-	-	48 h	×	○	○	[168]
Anostraca	brine shrimp (*Artemia franciscana*)	juvenile	PS	nonfunctionalized	bead	1, 3, 6, 10 μm	1-1000 particles/mL	-	-	30 d	✓	○	○	[169]
Anostraca	brine shrimp (*Artemia franciscana*)	juvenile	polymer	red opaque	sphere	1–5 μm	0.4, 0.8, 1.6 mg/L	-	-	44 d	×	×	✓	[170]
Anostraca	brine shrimp (*Artemia franciscana*)	nauplius juvenile	polymer	red fluorescent	sphere	1–5 μm	0.4, 1.6 mg/L	-	-	2 d<br>5 d	×<br>✓	×<br>×	○<br>○	[171]

Order/Subclass	Biological Species	Exposure Phase	MPs Type	Color	Shape	Size	Concentration	Type #	Concentration#	Exposure Time	Survival*	Growth and Development*	Reproduction*	Reference
Anostraca	brine shrimp (*Artemia franciscana*)	-	PS	-	bead	4–6 μm	0.2, 2 mg/L	-	-	14 d	✓	✓	○	[172]
Anostraca	brine shrimp (*Artemia franciscana*)	neonate	MPs<br>PET	-	particle<br>fiber	20-250 μm<br>20×100-500 μm	100 mg/L	-	-	48 h	×	✓	○	[110]
Anostraca	brine shrimp (*Artemia franciscana*)	larvae	PLA<br>PVDF	-<br>-	particle<br>fiber	25–350 μm<br>50–500×2.5-30 μm	1, 10, 100 mg/L	-	-	24 h	✓	○	○	[173]
Anostraca	brine shrimp (*Artemia franciscana*)	nauplius (48 h)	PP, PET	-	fiber	<500 μm	75-1000 mg/L	-	-	48 h	✓	○	○	[174]
Anostraca	brine shrimp (*Artemia parthenogenetica*)	nauplius (<24 h)	PS	-	sphere	10 μm	1-10000 particles/mL	-	-	24 h<br>14 d	×<br>×	○<br>×	○<br>○	[175]
Anostraca	brine shrimp (*Artemia	-	PE<br>PS	-	particle	40-220 μm<br>30-300 μm	100 mg/L	-	-	45 d	○	✓	○	[176]

Order/Subclass	Biological Species	Exposure Phase	MPs Type	Color	Shape	Size	Concentration	Type #	Concentration#	Exposure Time	Survival*	Growth and Development*	Reproduction*	Reference
	parthenogenetica)													
Anostraca	brine shrimp (*Artemia salina*)	1, 2, 7, 14 d,	PS	-	particle	5 μm	1-100 mg/L	-	-	48 h	×	×	○	[177]
							1 mg/L			14 d	✓	✓	○	
Anostraca	*Artemia salina*	adult	PS	-	sphere	50-70, 100-120 nm	0.5-200 μg/mL	-	-	24 h	✓	○	○	[178]
Anostraca	*Artemia salina*	nauplius	PS, PS-COOH, $PS\text{-}NH_2$	-	particle	6 μm	1 mg/L	nano-TiO_2	0.1, 1, 10 mg/L	48 h	✓	○	○	[179]
Anostraca	*Artemia salina*	2, 7, 14 d	PP	-	particle	11–44 μm	1-100 mg/L	-	-	48 h	✓	○	○	[180]
Anostraca	*Artemia salina*	4 w	PVC	-	particle	< 5 mm	0.26 mg/L	simvastatin<br>carbamazepine<br>TCS<br>CPF	5.8-12.03 mg/L<br>25.16-52.08 mg/L<br>0.0312-12 mg/L<br>0.00195-0.0312 mg/L	48 h	✓	○	○	[181]

Order/Subclass	Biological Species	Exposure Phase	MPs Type	Color	Shape	Size	Concentration	Type#	Concentration#	Exposure Time	Survival*	Growth and Development*	Reproduction*	Reference
Anostraca	*Thamnocephalus platyurus*	neonate	amidine PS, carboxyl PS	-	sphere	200 nm	10-400 mg/L	HSs, polysaccharide chains	1-5 mg/L	24 h	✓	○	○	[61]
Amphipoda	*Corophium volutator*	neonates (<24 h) -	PMMA, PMMA-PSMA	fluorescent	-	86-125 nm	0.01-500 mg/L	-	-	10 d	×	○	○	[104]
Amphipoda	*Echinogammarus marinus*	-	PS	red fluorescent	sphere	8 μm	~0.9, 9, 99 plastics/g food	-	-	35 d	✓	×	○	[182]
Amphipoda	*Hyalella azteca*	-	PS		bead	1 μm	1, 10, 100 particles/L	Cr (VI)	0–50 μg/L	28, 42 d	✓	✓	○	[67]
			PP	black	fiber	20-75×20 μm	0–90 fibers/mL							
Amphipoda	*Hyalella azteca*	juvenile (<7 d)	PE	blue fluorescent	particle	10-27 μm	0–100000 particles/mL	-	-	10 d	✓	✓	○	[183]
							0–20000 particles/mL			42 d	○	✓	✓	

Order/Subclass	Biological Species	Exposure Phase	MPs Type	Color	Shape	Size	Concentration	Type #	Concentration#	Exposure Time	Survival*	Growth and Development*	Reproduction*	Reference
Amphipoda	*Hyalella azteca*	-	polyester	-	fiber	26–5761 μm	0.15-150 mg/L; 0.002-2 g/kg dry wt	-	-	4, 14, 28 d	×	○	○	[108]
Amphipoda	*Hyalella azteca*	juvenile	tire wear particles	-	particle	<500 μm	0–15000 particles/mL	-	-	48 h	✓	○	○	[184]
		21-23 d					0-2000 particles/mL			21 d	✓	✓	✓	
Amphipoda	*Gmelinoides fasciatus*	-	PS	pink	irregular particle	<50 μm	54114 particles/mg	-	-	5 d	✓	○	○	[185]
						50–100 μm	7636 particles/mg							
						100–250 μm	122 particles/mg							
						>250 μm	66 particles/mg							
Amphipoda	*Gmelinoides fasciatus,*	-	LDPE	Nile Red	particle	53–100 μm	2 μg/L, 2 mg/L	-	-	14 d	✓	○	○	[186]

Order/Subclass	Biological Species	Exposure Phase	MPs Type	Color	Shape	Size	Concentration	Type#	Concentration#	Exposure Time	Survival*	Growth and Development*	Reproduction*	Reference
Amphipoda	*Gammarus lacustris*<br>*Gammarus duebeni*	adult	polyester	-	fiber	60×17 μm	600 fibers/mL	-	-	96 h	×	○	○	[187]
Amphipoda	*Gammarus fossarum*	-	PS<br>PA	fluorescent	bead<br>fiber	1.6 μm<br>500×20 μm	12500 beads/mL<br>2680 fibers/cm^2	-	-	28 d	○	✓	○	[188]
Amphipoda	*Gammarus fossarum*	-	PHB<br>PMMA	-	particle	32–63 μm	100000 particles/individual	-	-	28 d	○	✓	○	[189]
Amphipoda	*Gammarus roeseli*	-	PS<br>PLA	-	particle	30, 100, 500, 1000 nm<br>500, 2000 nm	4.31 ng/L<br>4.19 ng/L	-	-	14 d	✓	○	○	[190]
Amphipoda	*Gammarus roeseli*	adult	PA	-	particle	40-63 μm	500 μg/L	Phe	0-500 μg/L	24, 48 h	✓	○	○	[191]
Amphipoda	*Gammarus pulex*	juvenile, adult	PET	fluorescent	irregular fragment	10-150 μm	0.8-4000 particles/mL	-	-	24 h<br>48 d	○<br>×	✓<br>×	○<br>○	[192]

Order/Subclass	Biological Species	Exposure Phase	MPs Type	Color	Shape	Size	Concentration	Type#	Concentration#	Exposure Time	Survival*	Growth and Development*	Reproduction*	Reference
Amphipoda	*Gammarus pulex, Hyalella Azteca*	-	PS	-	irregular fragment	20-500 μm	0-40% PS weight in sediment	-	-	28 d	×	✓	○	[193]
Cirripedia	*Amphibalanus amphitrite*	stage II nauplii	PS<br>PVC<br>PMMA	fluorescent	sphere	113.8 nm<br>98.6 nm<br>101.7 nm	1-25 mg/L.	-	-	24 h	✓	○	○	[194]
Cirripedia	*Amphibalanus amphitrite*	II stage nauplius	PS	blue	bead	0.1 μm	0.001-10 mg/L	-	-	24, 48 h	×	○	○	[167]
Cirripedia	*Amphibalanus amphitrite*	nauplius larva, adult	PS	-	bead	1.7, 6.8, 10.4, 19 μm	1-1000 beads/mL	-	-	77 d	×	×	×	[195]
Cirripedia	*Amphibalanus amphitrite*	naupliar	PS	-	bead	1.7, 6.8, 10.4, 19 μm	1-1000 beads/mL	-	-	till cypris stage	×	×	○	[196]
Cirripedia	*Amphibalanus amphitrite*	stage II larvae	PS	-	sphere	3, 10 μm	500-10000 particles/mL	-	-	24 h	✓	✓	○	[197]
Cirripedia	nine barnacles	stage II naupli	PS	nonfluorescent	bead	1.7, 6.8, 10.4, 19 μm	1-1000 beads/mL	-	-	stage II	×	○	○	[198]

Order/Subclass	Biological Species	Exposure Phase	MPs Type	Color	Shape	Size	Concentration	Type#	Concentration#	Exposure Time	Survival*	Growth and Development*	Reproduction*	Reference
Cirripedia	*Amphibalanus amphitrite*	ar larva stage II nauplius	PMMA	fluorescent	particle	185 ± 3 nm	5, 10, 25 mg/L	-	-	24 h	×	○	○	[199]
Isopoda	*Asellus aquaticus*	-	PS	-	irregular fragment	20-500 µm	0-40% PS weight in sediment	-	-	28 d	×	✓	○	[193]
Isopoda	*Asellus aquaticus*	-	polyester	-	fiber	26–5761 µm	0.15-150 mg/L; 0.002-2 g/kg dry wt	-	-	4, 14, 28 d	×	○	○	[108]
			PS	green fluorescent	bead	10 µm	~12 beads/mg food							
Isopoda	*Idotea emarginata*	juvenile	PS	blue fluorescent	fragment	0-100 µm	20 fragments/mg food	-	-	42-49 d	×	×	○	[200]
			polyacrylic	orange fluorescent	fiber	-	0.3 mg fibers/mg food							

MPs, microplastics; PE, polyethylene; HDPE, high-density polyethylene; LDPE, low-density polyethylene; PS, polystyrene; PS-COOH, carboxylated-PS;

PS-NH_2, amino-PS; PP, polypropylene; PET, polyethylene terephthalate; PVC, polyvinyl chloride; PMMA, polymethyl methacrylate; PA, polyamide; PBT, polybutylene terephthalate; ABS, acrylonitrile-butadiene-styrene; PSMA, polystearyl methacrylate; PHA, polyhydroxyalkanoates; PHB, polyhydroxybutyrate; PUR, polyurethane; EAA, ethylene acrylic acid; PVDF, polyvinylidene difluoride; PLA, polylactic acid; PMA,polymethacrylic acid; PVA, polyvinyl alcohol; Cd, cadmium; Hg, mercury; Cu, copper; Zn, zinc; ZnO, zinc oxide; Ag, silver; $AgNO_3$, silver nitrate; Ni, nickel; Cr, chromium; Pb, lead; Li, lithium; Au, gold; TiO_2, titanium dioxide; As, arsenic; IMD, imidacloprid; AC, acyclovir; BaP, benzo(a)pyrene; DM, deltamethrin; Gly, glyphosate; BP-3, benzophenone-3; APFO, ammonium perfluorooctanoate; HA, humic acid; NOM, natural organic matter; DOM, dissolved organic matter; FA, fulvic acid; HSs, humic substances; MB, methylene blue; ROX, roxithromycin; TCS, triclosan; TCC, triclocarban; MTCS, methyl-triclosan; MLT, malathion; DBP, dibutyl phthalate; CPF, chlorpyrifos; PAH, polyvinyl alcohol; Fla, fluoranthene; LC_{50}, median lethal concentrations; w/w, mg/mg; #, other contaminants; *, life history.

Table S2. Effects of microplastics on behaviors of aquatic crustaceans. (✓ indicates involved, ○ indicates not involved).

Order/subclass	Biological species	Exposure phase	MPs type	Color	Shape	Size	Concentration	Type#	Concentration#	Exposure time	Feeding*	Swimming*	Grazing*	Defense*	Reference
Cladocera	*Ceriodaphnia dubia*	neonate (<24 h)	PE	white	bead	1-4 μm	0.5-16 mg/L	-	-	48 h	○	✓	○	○	[7]
			polyester	orange fluorescent	fiber	100-400 μm	0.125-4 mg/L								
							1/16-2 LC_{50}	PE beads	1/16-2 LC_{50}						
Cladocera	*Daphnia magna*	neonate (<24 h)	secondary PE MPs	fluorescent	particle bead	2.6 ± 1.8 μm 1–5 μm	2.25×10^5 particles/mL	-	-	72 h	✓	○	○	○	[25]
Cladocera	*Daphnia magna*	neonate (2 d)	PE	clear	sphere	32–38 μm	5, 40, 160 mg/L	-	-	1, 3, 5 h	○	✓	✓	○	[28]

Order/subclass	Biological species	Exposure phase	MPs type	Color	Shape	Size	Concentration	Type#	Concentration#	Exposure time	Feeding*	Swimming*	Grazing*	Defense*	Reference
Cladocera	*Daphnia magna*	juvenile (4 d)	PE	-	fragment	44.39 ± 11.16 μm<br>48.37 ± 6.26 μm	5 mg/L<br>4.5 mg/L	BP-3	-<br>0.5 mg/L	17 d	○	✓	○	○	[32]
Cladocera	*Daphnia magna*	juvenile (4 d)	PE	-	fragment	17.23 ± 3.43 μm, 34.43 ± 13.09 μm	5 mg/L	-	-	48 h	✓	○	○	○	[33]
Cladocera	*Daphnia magna*	neonate (<24 h)	PS-COOH	fluorescent	bead<br>particle	40-48 μm<br>20, 200 nm	100 mg/L	-	-	48 h	○	✓	○	○	[39]
Cladocera	*Daphnia magna*	neonate (<24 h)	PS	fluorescent	sphere	50 nm	0.05, 0.5 mg/L	-	-	21 d	○	✓	○	○	[41]
Cladocera	*Daphnia magna*	neonate (<24 h)	PS	-	sphere	72.84 ± 6.81 nm	15.6-500 mg/L	Gly	0-200 mg/L	48 h	○	✓	○	○	[49]
Cladocera	*Daphnia magna*	adult (21 d)	PS<br>PS-COOH<br>negative $PS\text{-}NH_2$<br>positive $PS\text{-}NH_2$	-	sphere	100 nm<br>300 nm<br>50–100 nm<br>110 nm	1 mg/L	-	-	48 h	○	✓	○	○	[50]

Order/subclass	Biological species	Exposure phase	MPs type	Color	Shape	Size	Concentration	Type#	Concentration#	Exposure time	Feeding*	Swimming*	Grazing*	Defense*	Reference
Cladocera	*Daphnia magna*	-	PS	fluorescent	sphere	100 nm, 2 μm	1 mg/L	-	-	24 h	✓	○	○	○	[51]
Cladocera	*Daphnia magna*	neonate	PS-COOH	fluorescent	particle	nanometer size	10 mg/L	-	-	2 h	✓	○	○	○	[52]
Cladocera	*Daphnia magna*	-	PS	-	particle	154.1 ± 2.9 nm	5 μg/L	-	-	until the 2th generation	○	✓	○	○	[59]
Cladocera	*Daphnia magna*	neonate (<24 h)	PS	fluorescent	sphere	1 μm	0.29 μg/ml	dimethoate<br>DM	0-5 mg /L<br>0-10 μg/L	72 h	○	✓	○	○	[68]
Cladocera	*Daphnia magna*	-	PS	red	bead	1, 10 μm	0.125, 1.25, 12.5 mg/L	-	-	7, 14, 21 d	○	✓	○	○	[71]
Cladocera	*Daphnia magna*	-	PS-COOH	red fluorescent	bead	2 μm	3.3310^{4} particles/mL	Zn	1.5, 2.5 mg/L	24 h	○	✓	○	○	[76]
Cladocera	*Daphnia magna*	juvenile (<96 h)	PS<br>aged PS	-	particle	<5 μm	~2×10^{5} particles/mL<br>~3.3×10^{5} particles/mL	-	-	24 h	✓	○	○	○	[78]
Cladocera	*Daphnia magna*	-	PS	color	bead	5 μm	6000, 12000, 24000	-	-	120 h	✓	○	○	○	[201]

Order/ subclass	Biological species	Exposure phase	MPs type	Color	Shape	Size	Concentration	Type#	Concentration#	Exposure time	Feeding*	Swimming*	Grazing*	Defense*	Reference
							particles/ mL								
Cladocera	*Daphnia magna*	neonate (<24 h)	aged PS	green fluorescent	sphere	5 μm	2.5 μg/L	ROX	0, 0.1, 10 μg/L	21 d	✓	✓	○	○	[79]
Cladocera	*Daphnia magna*	10 d	PS	-	sphere	~21.6 μm	25%, 75% in ratio of PS to food	-	-	7 d	✓	○	○	○	[86]
Cladocera	*Daphnia magna*	-	PS	-	sphere	<30 μm	16 μl/L	-	-	96 h	○	✓	○	○	[87]
Cladocera	*Daphnia magna*	7 d	PS	-	sphere	<30 μm	0-9.2 mg/L	-	-	7 d	✓	○	○	○	[202]
Cladocera	*Daphnia magna*	72 h	PP<br>PET	fluorescent	fiber	134.83 ± 88.70 μm<br>120.49 ± 82.46 μm	1000 mg/L	-	-	48 h	✓	○	○	○	[94]
Copepoda	*Acartia clausi*, *Centropages typicus*	adult female	polystyrene	red fluorescent	bead	6 μm	5000 beads/mL	-	-	5, 8 d	✓	○	○	○	[142]
Copepoda	several copepods	-	PS	yellow fluorescent	bead	7.3, 20.6, 30.6 μm	0.1% v/v	-	-	24 h	✓	○	○	○	[203]

Order/ subclass	Biological species	Exposure phase	MPs type	Color	Shape	Size	Concentration	Type#	Concentration#	Exposure time	Feeding*	Swimming*	Grazing*	Defense*	Reference
Copepoda	*Tigriopus japonicus*	adult -	PA 6<br>PE	-	particle	5-20 μm<br>10-30 μm	12.5−400 mg/L	-	-	24 h	✓	○	○	○	[149]
Copepoda	*Tigriopus japonicus*	-	PS	fluorescent	sphere	10 μm	2, 20, 200 μg/L	Cd	15.2 μg/L	24 d	✓	○	○	○	[148]
Copepoda	*Nitokra lacustris pacifca*	-	PS<br>biodegradable PHA	Nile red	bead	6.58 μm<br>7-140 μm	350, 700 beads/mL	-	-	24 h	✓	○	○	○	[154]
Copepoda	*Pseudodiaptomus annandalei*	adult	PS	nonfluorescent	sphere	0.5, 2, 10 μm	20, 200, 2000 μg/L	-	-	24 h	✓	○	○	○	[204]
Copepoda	*Acartia tonsa*	-	HDPE	-	particle	2-10 μm	0.1-100 mg/L	CPF	0-100 mg/L	24, 48 h	✓	○	○	○	[157]
Copepoda	*Temora longicornis*	adult	PE	-	aphere	10–45 μm	0-10 particles/ mL	-	-	48 h	✓	○	○	○	[205]
Copepoda	*Acartia tonsa, Temora longicornis*	-	tire wear MPs	-	particle	8-20 μm	0.01–10 particles/ mL	-	-	24 h	✓	○	○	○	[160]
Copepoda	*Calanus finmarchicus*	juvenile	nylon	-	fiber<br>granule	10×30 μm<br>10-30 μm	50 plastics/mL	-	-	3-4 d	✓	○	○	○	[162]

Order/subclass	Biological species	Exposure phase	MPs type	Color	Shape	Size	Concentration	Type#	Concentration#	Exposure time	Feeding*	Swimming*	Grazing*	Defense*	Reference
arctic Copepoda	*Calanus hyperboreus*	-	PE	clear	sphere	20.7 μm	20 particles/mL	crude oil	1 μl/L	5 d	✓	○	○	○	[206]
Copepoda	*Calanus helgolandicus*	adult female	PS	-	bead	6, 12, 26 μm	5000 beads/mL	-	-	up to 7 d	○	✓	○	○	[207]
Copepoda	*Calanus helgolandicus*	adult	PE	fluorescent	sphere	10-32 μm	100 plastics/mL	-	-	24 h	✓	○	○	○	[208]
			nylon 6,6		fragment	20 μm									
					fiber	10×40, 23×100 μm									
			PET			17×60, 23×70 μm									
Copepoda	*Calanus helgolandicus*	adult	PS	-	bead	20 μm	73 beads/mL	-	-	24 h	✓	○	○	○	[164]
Copepoda	*Calanus helgolandicus*	adult	nylon	-	fiber	10×30 μm	~80 fibers/mL	DMS	0, 1.2–1.3 nM	6 h	○	○	✓	○	[209]
Amphipoda	*Echinogammarus marinus*	-	PS	red fluorescent	sphere	8 μm	~0.9, 9, 99 plastics/g food	-	-	35 d	✓	○	○	○	[182]
Amphipoda	*Gmelinoides fasciatus,*	-	LDPE	Nile Red	particle	53–100 μm	2 μg/L, 2 mg/L	-	-	14 d	○	✓	○	○	[186]

Order/subclass	Biological species	Exposure phase	MPs type	Color	Shape	Size	Concentration	Type#	Concentration#	Exposure time	Feeding*	Swimming*	Grazing*	Defense*	Reference
	Gammarus lacustris														
Amphipoda	*Gammarus duebeni*	adult	PE<br>PS	red fluorescent<br>orange fluorescent	sphere	10–45 μm<br>1 μm	42.22 ± 8.25 plastics/L<br>175.9 ± 7.11 plastics/L	-	-	24, 96 h	✓	○	○	○	[210]
Amphipoda	*Gammarus fossarum*	-	PHB<br>PMMA		particle	32–63 μm	100000 particles/individual	-	-	28 d	✓	○	○	○	[189]
Amphipoda	*Gammarus roeseli*	-	PS<br>PLA	-	particle	30, 100, 500, 1000 nm<br>500, 2000 nm	4.31 ng/L<br>4.19 ng/L	-	-	14 d	✓	✓	○	○	[190]
Amphipoda	*Gammarus roeseli*	adult	PA	-	particle	40-63 μm	500 μg/L	Phe	0-500 μg/L	24, 48 h	✓	✓	○	○	[191]
Amphipoda	*Gammarus pulex*	juvenile, adult	PET	fluorescent	irregular fragment	10-150 μm	0.8-4000 particles/mL	-	-	48 d	✓	○	○	○	[192]

Order/subclass	Biological species	Exposure phase	MPs type	Color	Shape	Size	Concentration	Type#	Concentration#	Exposure time	Feeding*	Swimming*	Grazing*	Defense*	Reference
Amphipoda	*Gammarus pulex*	-	PMMA	transparent	sphere	40.2 μm	0-104.48 particles/cm^2	-	-	24 h	✓	○	○	○	[211]
Amphipoda	*Gammarus pulex*, *Hyalella Azteca*	-	PS	-	irregular fragment	20-500 μm	0-40% PS weight in sediment	-	-	28 d	✓	○	○	○	[193]
Decapoda	marine mysid (*Neomysis awatschensis*)	juvenile	PS	fluorescent	bead	1, 10 μm	1×10^3–5×10^5 particles/mL	-	-	96 h	✓	○	○	○	[127]
Decapoda	mysid shrimp (*Neomysis japonica*)	larva	PS, PS–COOH	fluorescent	bead	5 μm	10-6250 μg/L	-	-	96 h	✓	✓	○	○	[128]
Decapoda	shore crab (*Carcinus maenas*)	-	PE	colored	particle	40–48 μm	~65-114, ~650-1140 particles/L	-	-	8 h, 5 d	✓	○	○	○	[212]
Decapoda	crab (*Carcinus maenas*)	-	PP	blue	fiber	500 μm	0.3%, 0.6%, 1% in food	-	-	1 month	✓	○	○	○	[136]
Decapoda	hermit crab (*Pagurus bernhardus*)	-	PE	-	sphere	4 mm	25 particles/L	-	-	5 d	○	○	○	✓	[213]

Order/ subclass	Biological species	Exposure phase	MPs type	Color	Shape	Size	Concentration	Type#	Concentration#	Exposure time	Feeding*	Swimming*	Grazing*	Defense*	Reference
Decapoda	langoustine (*Nephrops norvegicus*)	-	PP	-	fiber	3-5×0.2 mm	-	-	-	8 months	✓	○	○	○	[139]
Anostraca	brine shrimp (*Artemia franciscana*)	-	PS	-	particle	40-50 nm	1, 10, 100 mg/L	HA	0, 5 mg/L	48 h	○	✓	○	○	[165]
Anostraca	brine shrimp (*Artemia franciscana*)	nauplius	$PS\text{-}NH_2$	-	sphere	50 nm	0.1, 1 mg/L	-	-	7, 14 d	✓	○	○	○	[166]
Anostraca	brine shrimp (*Artemia franciscana*)	Instar I larva	PS	blue	bead	0.1 μm	0.001-10 mg/L	-	-	24, 48 h	○	✓	○	○	[167]
Anostraca	brine shrimp (*Artemia franciscana*)	nauplius<br>juvenile	polymer	red fluorescent	sphere	1–5 μm	0.4, 1.6 mg/L	-	-	2 d<br>5 d	✓	○	○	○	[171]
Anostraca	brine shrimp (*Artemia franciscana*)	larva	PLA<br>PVDF	-<br>-	particle<br>fiber	25–350 μm<br>50–500×2.5-30 μm	1, 10, 100 mg/L	-	-	24 h	○	✓	○	○	[173]
Anostraca	brine shrimp (*Artemia	larva	PS	-	sphere	10 μm	10-10000 particles/mL	-	-	24 h	✓	○	○	○	[214]

Order/subclass	Biological species	Exposure phase	MPs type	Color	Shape	Size	Concentration	Type#	Concentration#	Exposure time	Feeding*	Swimming*	Grazing*	Defense*	Reference
	parthenogenetica)														
Isopoda	*Asellus aquaticus*	-	PS	-	irregular fragment	20-500 μm	0-40% PS weight in sediment	-	-	28 d	✓	○	○	○	[193]
Isopoda	*Idotea emarginata*	-	PMMA	fluorescent	particle	10-100 μm	40 particles/mg food	-	-	8 d	✓	○	○	○	[215]
Cirripedia	*Amphibalanus amphitrite*	nauplius larva, adult	PS	-	bead	1.7, 6.8, 10.4, 19 μm	1-1000 beads/mL	-	-	77 d	✓	○	○	○	[195]
Cirripedia	*Amphibalanus amphitrite*	naupliar	PS	-	bead	1.7, 6.8, 10.4, 19 μm	1000 beads/mL	-	-	24 h	✓	○	○	○	[196]
Cirripedia	*Amphibalanus amphitrite*	II stage nauplius	PS	blue	bead	0.1 μm	0.001-10 mg/L	-	-	24, 48 h	○	✓	○	○	[167]

MPs, microplastics; PE, polyethylene; HDPE, high-density polyethylene; LDPE, low-density polyethylene; PS, polystyrene; PS-COOH, carboxylated-PS; PS-NH_2, amino-PS; PMMA, polymethyl methacrylate; PET, polyethylene terephthalate; PA, polyamide; PHA, polyhydroxyalkanoates; PHB, polyhydroxybutyrate; Zn, zinc; Cd, cadmium; BP-3, benzophenone-3; Gly, glyphosate; DM, deltamethrin; ROX, roxithromycin; CPF, chlorpyrifos; DMS, dimethyl sulfide; Phe, phenanthrene; HA, humic acid; LC_{50}, median lethal concentrations; #, other contaminants; *, behaviors.

Table S3. Effects of microplastics on physiological functions of aquatic crustaceans. (✓ indicates involved, O indicates not involved).

Order/subclass	Biological species	Exposure phase	MPs type	Color	Shape	Size	Concentration	Type#	Concentration#	Exposure time	Oxidative damage*	Neuromodulation*	Energy regulation*	Metabolic regulation*	Respiratory regulation*	Immunomodulation*	Intestinal physiology*	Reference
Decapoda	white-leg shrimp (*Litopenaeus vannamei*)	juvenile	PE	red fluorescent	particle	5 μm	50, 500, 5000 μg/L	-	-	48 h	O	O	O	O	O	O	✓	[118]
Decapoda	*Litopenaeus vannamei*	juvenile	PE	red fluorescent	sphere	10-22 μm	0.1-1 μg/g shrimp	-	-	7 d	✓	O	O	O	O	O	O	[119]
Decapoda	whiteleg shrimp (*Litopenaeus vannamei*)	-	PS	blue	sphere	44 nm	50 mg/L	-	-	21 d	✓	O	✓	O	O	O	O	[120]
Decapoda	Pacific white shrimp (*Litopenaeus*	-	PS	-	sphere	100 nm	200, 2000 mg/kg	TiO_2	200, 2000 mg/kg	28 d	O	O	O	O	O	O	✓	[121]

Order/subclass	Biological species	Exposure phase	MPs type	Color	Shape	Size	Concentration	Type #	Concentration#	Exposure time	Oxidative damage*	Neuromodulation*	Energy regulation*	Metabolic regulation*	Respiratory regulation*	Immunomodulation*	Intestinal physiology*	Reference
	vannamei)																	
Decapoda	Pacific whiteleg shrimp (*Litopenaeus vannamei*)	-	PS	-	particle	500 nm	0.69 mg/L	BPA	0, 2 μg/L	14 d	○	○	○	✓	✓	○	○	[216]
Decapoda	Pacific white shrimp (*Litopenaeus vannamei*)	juvenile	PE<br>PS<br>PP<br>PVC<br>PTFE	white	irregular particle	6-18 μm<br>100-200 μm<br>1.77-18 μm<br>1-13 μm<br>1-8 μm	1 mg/L	-	-	14 d	○	○	○	✓	○	○	✓	[217]
Decapoda	whiteleg shrimp (*Penaeu*	-	weathered PE	-	particle	32-43 μm	1-5 μg/L	-	-	25 d	✓	○	○	○	○	○	○	[122]

Order/subclass	Biological species	Exposure phase	MPs type	Color	Shape	Size	Concentration	Type #	Concentration#	Exposure time	Oxidative damage*	Neuromodulation*	Energy regulation*	Metabolic regulation*	Respiratory regulation*	Immunomodulation*	Intestinal physiology*	Reference
	s vannamei)																	
Decapoda	shrimp (*Macrobrachium nipponense*)	juvenile	PS	-	sphere	75 nm	5-40 mg/L	-	-	28 d	✓	○	○	○	○	✓	○	[129]
Decapoda	brown shrimp (*Crangon crangon*)	-	MPs	white	particle	<60 µm	20 mg/L	-	-	48 h	✓	○	○	○	○	○	○	[218]
Decapoda	mysid (*Neomysis awatschensis*)	juvenile, adult	PS	-	beads	1 µm	10 particles/mL	As, Cd, Cu, Pb, Zn	20–100 mg/L	96 h	✓	✓	○	○	○	○	○	[126]
Decapoda	marine mysid (*Neomysis awatschensis*)	juvenile, adult	PS	fluorescent	bead	1, 10 µm	5 ×10^5 particles/mL	-	-	96 h	✓	○	○	○	○	○	○	[127]

Order/subclass	Biological species	Exposure phase	MPs type	Color	Shape	Size	Concentration	Type#	Concentration#	Exposure time	Oxidative damage*	Neuromodulation*	Energy regulation*	Metabolic regulation*	Respiratory regulation*	Immunomodulation*	Intestinal physiology*	Reference
Decapoda	oriental river prawn (*Macrobrachium nipponense*)	juvenile	PS	-	sphere	75 nm	5-40 mg/L	-	-	28 d	○	○	○	✓	○	○	○	[130]
Decapoda	prawn (*Macrobrachium nipponense*)	adult	PS	-	sphere	500 nm	0.04-40 mg/L	-	-	28 d	✓	○	○	○	○	○	○	[131]
Decapoda	oriental river prawn (*Macrobrachium nipponense*)	-	PS	red fluorescent	sphere	5 μm	2, 20 mg/L	-	-	28 d	✓	○	○	○	○	✓	○	[132]
Decapoda	giant river prawn (*Macrobrachium*	juvenile	PS<br>PE	-	sphere<br>irregular	0.5–1 μm<br>30-150 μm	1, 5, 10 mg/100 g food	-	-	60 d	✓	○	○	✓	○	○	○	[133]

Order/subclass	Biological species	Exposure phase	MPs type	Color	Shape	Size	Concentration	Type #	Concentration#	Exposure time	Oxidative damage*	Neuromodulation*	Energy regulation*	Metabolic regulation*	Respiratory regulation*	Immunomodulation*	Intestinal physiology*	Reference
	rosenbergii)				flake													
Decapoda	swamp ghost crab (*Ucides cordatus*)	adult	PE	-	particle	100-250 μm	250 mg/L	TCS	1 mg/L	7 d	✓	✓	○	○	○	○	○	[219]
Decapoda	*Ucides cordatus*	-	PE	-	particle	100-250 μm	250 mg/	-	-	7 d	✓	✓	○	○	○	○	○	[220]
Decapoda	Chinese mitten crab (*Eriocheir sinensis*)	juvenile	PS	virgin	sphere	5 μm	0.04-40 mg/L	-	-	21 d	✓	✓	○	○	○	○	○	[135]
Decapoda	Chinese mitten crab (*Eriocheir*	juvenile	PS	-	sphere	5 μm	0.04-40 mg/L	-	-	21 d	○	○	○	○	○	✓	✓	[221]

Order/subclass	Biological species	Exposure phase	MPs type	Color	Shape	Size	Concentration	Type #	Concentration#	Exposure time	Oxidative damage*	Neuromodulation*	Energy regulation*	Metabolic regulation*	Respiratory regulation*	Immunomodulation*	Intestinal physiology*	Reference
	sinensis)																	
Decapoda	Chinese mitten crab (*Eriocheir sinensis*)	juvenile	PS	fluorescent	sphere	5 μm	400 μg/L	Pb	0, 5, 50 μg/L	21 d	✓	○	○	✓	○	○	○	[222]
Decapoda	predatory marine crab (*Charybdis japonica*)	-	PS	green fluorescent	sphere	5 μm	68 μg/L	-	-	7 d	✓	✓	○	○	○	○	○	[223]
Decapoda	shore crab (*Carcinus maenas*)	-	PS	-	sphere	8 μm	10^6, 10^7 spheres/L	-	-	1, 16, 24 h	○	○	○	○	✓	○	○	[224]

Order/subclass	Biological species	Exposure phase	MPs type	Color	Shape	Size	Concentration	Type#	Concentration#	Exposure time	Oxidative damage*	Neuromodulation*	Energy regulation*	Metabolic regulation*	Respiratory regulation*	Immunomodulation*	Intestinal physiology*	Reference
Decapoda	crab (*Carcinus maenas*)	-	PP	blue	fiber	500 μm	0.3%, 0.6%, 1% in food	-	-	28 d	○	○	✓	○	○	○	○	[136]
Decapoda	langoustine (*Nephrops norvegicus*)	-	PP	-	fiber	3-5×0.2 mm	-	-	-	8 months	○	○	✓	✓	○	○	○	[139]
Decapoda	crayfish (*Procambarus clarkii*)	-	PE	Nile red	powder	3-20 μm	0.5‰, 1‰, 2‰ weight in food	-	-	21 d	✓	○	○	○	○	✓	✓	[225]
Decapoda	crayfish (*Procambarus clarkii*)	-	PS	-	bead	75 nm	5, 10, 20 mg/L	-	-	48 h	✓	○	○	○	○	✓	✓	[226]
Decapoda	red crayfish (*Cherax quadricarinatus*)	-	PS	green fluorescent	sphere	80 nm	25, 250, 2500 μg/L	-	-	14 d	✓	○	○	○	○	○	✓	[227]

Order/subclass	Biological species	Exposure phase	MPs type	Color	Shape	Size	Concentration	Type#	Concentration#	Exposure time	Oxidative damage*	Neuromodulation*	Energy regulation*	Metabolic regulation*	Respiratory regulation*	Immunomodulation*	Intestinal physiology*	Reference
Decapoda	redclaw crayfish (*Cherax quadricarinatus*)	juvenile	PS	fluorescent	sphere	200 nm	0.5, 5 mg/L	-	-	21 d	○	○	○	✓	○	○	○	[140]
Decapoda	Atlantic ditch shrimp (*Palaemon varians*)	intermolt stage	PS	fluorescent	sphere	0.1 μm<br>2.1 μm<br>9.9 μm	2000 n/mL<br>1000 n/mL<br>200 n/mL	-	-	48 h	✓	○	○	○	○	○	○	[228]
Decapoda	*Gammarus pulex*	-	PET	fluorescent	irregular fragment	10-150 μm	0.8-4000 particles/mL	-	-	48 d	○	○	✓	○	○	○	○	[192]
Decapoda	American lobster (*Homarus americanus*)	larva	PET	neon pink	fiber	459 ± 2.25 μm	25 fibers/mL	-	-	5 d	○	○	○	○	✓	○	○	[113]

Order/subclass	Biological species	Exposure phase	MPs type	Color	Shape	Size	Concentration	Type #	Concentration#	Exposure time	Oxidative damage*	Neuromodulation*	Energy regulation*	Metabolic regulation*	Respiratory regulation*	Immunomodulation*	Intestinal physiology*	Reference
Cladocera	*Ceriodaphnia dubia*	neonate (<24 h)	PS	-	bead	1 μm	0.85-8500 μg/L	-	-	24 h	✓	○	○	○	○	○	○	[8]
Cladocera	*Moina monogolica*	neonate (12-24 h)	aged PE	no	particle	2-4 μm	100, 300 μg/L	Cd	0, 5, 10 μg/L	21 d	○	○	✓	○	○	○	○	[1]
Cladocera	*Moina macrocopa*	neonate (<24 h)	PS	-	bead	1 μm	0.001-500 μg/L	-	-	1, 7 d	✓	✓	○	○	○	○	○	[4]
Cladocera	*Diaphanosoma celebensis*	4 d	PS	-	sphere	0.05, 0.5, 6 μm	1, 10 mg/L	-	-	48 h	✓	○	○	○	○	○	○	[10]
Cladocera	*Diaphanosoma celebensis*	4 d	PS	-	bead	0.05, 0.5, 6 μm	1 mg/L	Hg	0.8 μg/L	48 h	✓	○	○	○	○	○	○	[12]
Cladocera	*Daphnia pulex*	neonate (<24 h)	PS	-	sphere	71.18 nm	0.1-2 mg/L	-	-	21 d	✓	○	○	○	○	○	○	[14]

Order/subclass	Biological species	Exposure phase	MPs type	Color	Shape	Size	Concentration	Type#	Concentration#	Exposure time	Oxidative damage*	Neuromodulation*	Energy regulation*	Metabolic regulation*	Respiratory regulation*	Immunomodulation*	Intestinal physiology*	Reference
Cladocera	*Daphnia pulex*	neonate (<24 h)	PS	-	sphere	75 nm	1 μg/L	-	-	21 d	✓	○	○	○	○	○	○	[15]
Cladocera	*Daphnia pulex*	neonate (<24 h)	PS	-	sphere	75 nm	0.1-2 mg/L	-	-	21 d	✓	○	○	○	○	○	○	[229]
Cladocera	*Daphnia pulex*	neonate (<24 h)	PS	-	sphere	500 nm	0.5-4 mg/L	-	-	14 d	✓	○	○	○	○	○	○	[19]
Cladocera	*Daphnia magna*	4-5 d	PE	yellow	sphere	20, 30 μm	20 mg/L	-	-	24 h	○	○	○	✓	○	○	○	[27]
Cladocera	*Daphnia magna*	4 d	PE	-	fragment	37.24 ± 11.76 μm	0.05, 0.1, 0.5, 1 mg/L	BP-3	0, 10 % w/w	48 h	✓	○	○	○	○	○	○	[29]
Cladocera	*Daphnia magna*	juvenile (4 d)	PE	-	fragment	44.39 ± 11.16 μm<br>48.37 ± 6.26 μm	5 mg/L<br>4.5 mg/L	BP-3	-<br>0.5 mg/L	17 d	✓	○	○	○	○	○	○	[32]

Order/subclass	Biological species	Exposure phase	MPs type	Color	Shape	Size	Concentration	Type #	Concentration#	Exposure time	Oxidative damage*	Neuromodulation*	Energy regulation*	Metabolic regulation*	Respiratory regulation*	Immunomodulation*	Intestinal physiology*	Reference
Cladocera	*Daphnia magna*	juvenile (4 d)	PE	-	fragment<br>bead	17.23 ± 3.43 µm, 34.43 ± 13.09 µm<br>40-48 µm	5 mg/L	-	-	21 d	○	○	✓	○	○	○	○	[33]
Cladocera	*Daphnia magna*	neonate (<24 h)	PS	fluorescent	sphere	50 nm	0.05, 0.5 mg/L	-	-	21 d	✓	○	✓	○	○	○	○	[41]
Cladocera	*Daphnia magna*	neonate (<24 h)	PS, PS-COOH, PS-NH_2	-	sphere	50-100 nm	-	-	-	48 h	✓	○	○	○	○	○	○	[42]
Cladocera	*Daphnia magna*	8 d	PS	-	sphere	72.84 ± 6.81 nm	15.6-500 mg/L	Gly	0-200 mg/L	48 h	✓	○	○	○	○	○	○	[49]
Cladocera	*Daphnia magna*	-	PS<br>PS-COOH	-	sphere	100 nm<br>300 nm	1 mg/L	-	-	48 h	✓	✓	○	○	○	○	○	[50]

Order/subclass	Biological species	Exposure phase	MPs type	Color	Shape	Size	Concentration	Type#	Concentration#	Exposure time	Oxidative damage*	Neuromodulation*	Energy regulation*	Metabolic regulation*	Respiratory regulation*	Immunomodulation*	Intestinal physiology*	Reference
Cladocera	*Daphnia magna*	adult	negative PS-NH_2<br>positive PS-NH_2<br>PS<br>PE	-	sphere	50–100 nm<br>110 nm<br>300, 600 nm<br>0.3-9 µm	1 mg/L	Ag, DOM	1 µg/L Ag, 1, 10, 50 mg/L DOM	72 h	✓	○	○	○	○	○	○	[63]
Cladocera	*Daphnia magna*	neonate	PS-COOH	fluorescent	sphere	500 nm	1 mg/L	-	-	till produce second clutch	○	○	○	○	○	✓	○	[64]
Cladocera	*Daphnia magna, Daphnia pulex*	-	PS	Nile Red	bead	1 µm	200 µg/L	-	-	72 h	○	○	○	✓	○	○	○	[230]

Order/subclass	Biological species	Exposure phase	MPs type	Color	Shape	Size	Concentration	Type #	Concentration#	Exposure time	Oxidative damage*	Neuromodulation*	Energy regulation*	Metabolic regulation*	Respiratory regulation*	Immunomodulation*	Intestinal physiology*	Reference
Cladocera	*Daphnia magna*	7 d	PS	-	bead	1, 10 μm	0.1 mg/L	ROX	0.01 mg/L	48 h	✓	○	○	○	○	○	○	[72]
Cladocera	*Daphnia magna*	-	PS-COOH	red fluorescent	bead	2 μm	1 mg/L	Zn	1.5, 2.5 mg/L	24 h	✓	○	○	○	○	○	○	[76]
Cladocera	*Daphnia magna*	neonate (<24 h)	aged PS	green fluorescent	sphere	5 μm	2.5 μg/L	ROX	0, 0.1, 10 μg/L	21 d	✓	✓	○	○	○	○	○	[79]
Cladocera	*Daphnia magna*	-	secondary MPs (PET, PS, ABS)	-	particle	3.2-3.7 μm	1 % of diet	-	-	14 d	○	○	✓	○	○	○	○	[77]
Cladocera	*Daphnia magna*	neonate (<24 h)	PVC	-	-	2 ± 1 μm<br>50 ± 10 μm	2.05 mg/L<br>4.97 mg/L	-	-	48 h	✓	○	○	○	○	○	○	[95]
Cladocera	*Daphnia magna*	5 d	PET	-	fibers	360×14 μm	100 mg/L	Ag, $AgNO_3$	0, 0.33 μg Ag/L	48 h	○	○	✓	○	○	○	○	[97]

Order/subclass	Biological species	Exposure phase	MPs type	Color	Shape	Size	Concentration	Type#	Concentration#	Exposure time	Oxidative damage*	Neuromodulation*	Energy regulation*	Metabolic regulation*	Respiratory regulation*	Immunomodulation*	Intestinal physiology*	Reference
Anostraca	brine shrimp (*Artemia franciscana*)	nauplius，	PS-NH_2	-	sphere	50 nm	0.1, 1 mg/L	-	-	48 h, 14 d	✓	✓	○	○	○	○	○	[166]
Anostraca	brine shrimp (*Artemia franciscana*)	Instar I larva	PS	blue	bead	0.1 μm	0.001-1 mg/L	-	-	48 h	✓	✓	○	○	○	○	○	[167]
Anostraca	brine shrimp (*Artemia franciscana*)	juvenile	PS	nonfunctionalized	bead	1, 10 μm	1, 1000 particles/mL	-	-	96 h	✓	○	○	○	○	○	○	[169]
										96 h	✓	○	○	○	○	○	○	
Anostraca	brine shrimp (*Artemia franciscana*)	nauplius<br>juvenile	polymer	red fluorescent	sphere	1–5 μm	0.4, 1.6 mg/L	-	-	2 d<br>5 d	✓	✓	○	○	○	○	○	[171]

Order/subclass	Biological species	Exposure phase	MPs type	Color	Shape	Size	Concentration	Type#	Concentration#	Exposure time	Oxidative damage*	Neuromodulation*	Energy regulation*	Metabolic regulation*	Respiratory regulation*	Immunomodulation*	Intestinal physiology*	Reference
Anostraca	brine shrimp (*Artemia franciscana*)	-	PS	-	bead	4–6 µm	0, 0.2, 2.0 mg/L	-	-	14 d	✓	○	○	○	○	✓	○	[172]
Anostraca	brine shrimp (*Artemia parthenogenetica*)	-	PE	-	particle	40-220 µm	100 mg/L	-	-	45 d	○	○	○	○	○	○	✓	[176]
Anostraca	brine shrimp (*Artemia salina*)	1, 2, 7, 14 d,	PS	-	particle	5 µm	1-100 mg/L<br>1 mg/L	-	-	48 h<br>14 d	✓	○	○	○	○	○	○	[177]
Anostraca	*Artemia salina*	adult	PS	-	sphere	50-70 nm<br>100-120 nm	0.41, 4.82, 56.7 mg/L<br>0.787, 8.79, 75.6 mg/L	-	-	24 h	✓	○	○	○	○	○	○	[178]

Order/subclass	Biological species	Exposure phase	MPs type	Color	Shape	Size	Concentration	Type#	Concentration#	Exposure time	Oxidative damage*	Neuromodulation*	Energy regulation*	Metabolic regulation*	Respiratory regulation*	Immunomodulation*	Intestinal physiology*	Reference
Anostraca	*Artemia salina*	nauplius	PS, PS-COOH, PS-NH_2	-	particle	6 µm	1 mg/L	nano-TiO_2	0.1, 1, 10 mg/L	48 h	✓	○	○	○	○	○	○	[179]
Anostraca	*Artemia salina*	2, 7, 14 d	PP	-	particle	11–44 µm	1-100 mg/L	-	-	48 h	✓	✓	○	○	○	○	○	[180]
Anostraca	*Artemia salina*	4 w	PVC	-	particle	< 5 mm	0.26 mg/L	simvastatin<br>carbamazepine<br>TCS<br>CPF	5.8-12.03 mg/L<br>25.16-52.08 mg/L<br>0.0312-12 mg/L<br>0.00195-0.0312 mg/L	48 h	○	✓	○	○	○	○	○	[181]
Copepoda	*Acartia clausi*, *Centrop*	adult female	polystrene	red fluorescent	bead	6 µm	5000 beads/mL	-		5 d	○	○	○	○	✓	○	○	[142]

Order/subclass	Biological species	Exposure phase	MPs type	Color	Shape	Size	Concentration	Type #	Concentration#	Exposure time	Oxidative damage*	Neuromodulation*	Energy regulation*	Metabolic regulation*	Respiratory regulation*	Immunomodulation*	Intestinal physiology*	Reference
	ages typicus																	
Copepoda	*Tigriopus japonicus*	-	PS	fluorescent	bead	50 nm, 2 μm	0.5 μg/L, 100 mg/L	-	-	30 d	✓	○	○	○	○	○	○	[144]
Copepoda	*Tigriopus japonicus*	adult	PS	-	bead	50 nm, 10 μm	20 mg/L	-	-	24 h	✓	○	○	○	○	○	○	[145]
Copepoda	*Paracyclopina nana*	-	PS	-	bead	0.05, 0.5, 6 μm	0.1-20 mg/L	-	-	24 h	✓	○	○	○	○	○	○	[156]
Copepoda	*Calanus helgolandicus*	egg	PS	-	bead	20 μm	65 beads/mL	-	-	9 d	○	○	✓	○	✓	○	○	[164]
Copepoda	*Calanus helgolandicus*	adult female	PS	-	bead	6, 12, 26 μm	5000 beads/mL	-	-	3 d	○	○	✓	○	✓	○	○	[207]
Copepoda	*Calanus finmarchicus*	juvenile	nylon	-	fiber granule	10×30 μm 10-30 μm	50 plastics/mL	-	-	6 d	○	○	✓	○	○	○	○	[162]
		-	PS	-	particle	30, 100,	4.31 ng/L	-	-	14 d	○	○	✓	○	○	○	○	[190]

Order/subclass	Biological species	Exposure phase	MPs type	Color	Shape	Size	Concentration	Type #	Concentration#	Exposure time	Oxidative damage*	Neuromodulation*	Energy regulation*	Metabolic regulation*	Respiratory regulation*	Immunomodulation*	Intestinal physiology*	Reference
Amphipoda	*Gammarus roeseli*		PLA			500, 1000 nm 500, 2000 nm	4.19 ng/L											
Amphipoda	*Gmelinoides fasciatus, Gammarus lacustris*	-	LDPE	Nile Red	particle	53–100 μm	2 μg/L, 2 mg/L	-	-	14 d	✓	○	○	○	○	○	○	[186]
Amphipoda	*Gammarus pulex*	-	PMMA	transparent	sphere	40.2 μm	0-104.48 particles/cm^2	-	-	24 h	○	○	○	✓	✓	○	○	[211]
Cirripedia	*Amphibalanus amphitrite*	II stage nauplius	PS	blue	bead	0.1 μm	0.001-1 mg/L	-	-	48 h	✓	✓	○	○	○	○	○	[167]
Isopoda	*Idotea emarginata*	-	PMMA	fluorescent	particle	10-100 μm	40 particles/mg food	-	-	8 d	○	○	○	✓	○	○	○	[215]

MPs, microplastics; PE, polyethylene; LDPE, low-density polyethylene; PS, polystyrene; PS-COOH, carboxylated-PS; PS-NH_2, amino-PS; PVC, polyvinyl chloride; PMMA, polymethyl methacrylate; PET, polyethylene terephthalate; ABS, acrylonitrile-butadiene-styrene; PTFE, polytetrafluoroethylene; TiO_2, titanium dioxide; Zn, zinc; As, arsenic; Cd, cadmiuml; Cu, copper; Pb, lead; Ag, silver; $AgNO_3$, silver nitrate; Pb, lead; Hg, mercury; BPA, bisphenol A; TCS, triclosan; BP-3, benzophenone-3; Gly, glyphosate; DOM, dissolved organic matter; ROX, roxithromycin; CPF, chlorpyrifos; w/w, mg/mg; #, other contaminants; *, physiological functions.

Table S4. Molecular mechanism of microplastics on aquatic crustaceans. (✓ indicates involved, O indicates not involved).

Order/ subclass	Biological species	Exposure phase	MPs type	Color	Shape	Size	Concentration	Type #	Concentration#	Exposure time	Gene-level changes*	Protein-level changes*	Reference
Cladocera	*Moina macrocopa*	neonate (<24 h)	PS	-	bead	1 μm	0.001-500 μg/L	-	-	7 d	✓	O	[4]
Cladocera	*Diaphanosoma celebensis*	4 d	PS	unlabeled	sphere	0.05, 0.5, 6 μm	0.1, 1, 10 mg/L	-	-	48 h	✓	O	[10]
Cladocera	*Diaphanosoma celebensis*	-	PS	-	bead	0.05, 0.5, 6 μm	0.1, 1, 10 mg/L	-	-	48 h	✓	O	[11]
Cladocera	*Diaphanosoma celebensis*	4 d	PS	-	bead	0.05, 0.5, 6 μm	1 mg/L	Hg	0.2, 0.4, 0.8 μg/L	48 h	✓	O	[12]
Cladocera	*Daphnia pulex*	neonate (<24 h)	PS	-	sphere	71.18 nm	0.1-2 mg/L	-	-	21 d	✓	✓	[14]
Cladocera	*Daphnia pulex*	neonate (<24 h)	PS	-	sphere	71.18 ± 6.03 nm	1 mg/L	-	-	96 h	✓	O	[231]
Cladocera	*Daphnia pulex*	neonate (<24 h)	PS	-	sphere	75 nm	1 μg/L	-	-	21 d	✓	O	[15]
Cladocera	*Daphnia pulex*	1, 4, 7, 14, 21 d	PS	unlabeled	sphere	75 nm	0.1, 1 mg /L	-	-	96 h	✓	O	[17]
Cladocera	*Daphnia pulex*	neonate (<24 h)	PS	-	sphere	75 nm	1 mg/L	-	-	21 d	✓	O	[16]
Cladocera	*Daphnia pulex*	neonate (<24 h)	PS	-	sphere	75 nm	0.1-2 mg/L, 1 μg/L	-	-	21 d	✓	O	[232]

Order/ subclass	Biological species	Exposure phase	MPs type	Color	Shape	Size	Concentration	Type #	Concentration#	Exposure time	Gene-level changes*	Protein-level changes*	Reference
Cladocera	*Daphnia pulex*	neonate (<24 h)	PS	-	sphere	75 nm	0.1-2 mg/L	-	-	21 d	✓	✓	[229]
Cladocera	*Daphnia pulex*	neonate (<24 h)	PS	-	sphere	75 nm	0.1-2 mg/L	-	-	21 d	✓	○	[233]
Cladocera	*Daphnia pulex*	neonate (<24 h)	PS	-	sphere	75 nm	0.1-2 mg/L	-	-	21 d	✓	○	[18]
Cladocera	*Daphnia pulex*	neonate (<24 h)	PS	-	sphere	500 nm	1 mg/L	-	-	14 d	○	✓	[19]
Cladocera	*Daphnia magna*	neonate (<24 h)	PS	fluorescent	sphere	50 nm	0.05, 0.5 mg/L	-	-	21 d	✓	○	[41]
Cladocera	*Daphnia magna*	-	PS<br>PS-COOH<br>negative PS-NH$_2$<br>positive PS-NH$_2$	-	sphere	100 nm<br>300 nm<br>50–100 nm<br>110 nm	1 mg/L	-	-	48 h	○	✓	[50]
Cladocera	*Daphnia magna*	neonate (<24 h)	PS-NH$_2$	-	sphere	0.1-0.12 μm	200 mg/L	HA	0, 5 mg/L	96 h	✓	○	[57]
Cladocera	*Daphnia magna*	neonate	PS-NH$_2$	-	sphere	0.10-0.12 μm	50, 100 mg/L	NOM<br>FA<br>HA	10 mg/L	96 h	✓	○	[58]
Cladocera	*Daphnia magna*	neonate	PS	-	bead	1.25 μm	2, 4, 8 mg/L	-	-	10 d	✓	○	[234]
Cladocera	*Daphnia magna*	6 d	PS	unlabeled	sphere	1.25 μm	0.5, 1, 2 mg/L	-	-	24 h	✓	○	[73]

Order/subclass	Biological species	Exposure phase	MPs type	Color	Shape	Size	Concentration	Type#	Concentration#	Exposure time	Gene-level changes*	Protein-level changes*	Reference
Cladocera	*Daphnia magna*	-	PS-COOH	red fluorescent	bead	2 µm	1 mg/L	Zn	1.5, 2.5 mg/L	24 h	✓	○	[76]
Cladocera	*Daphnia magna*	neonate (<24 h)	PS	-	particle	13.03 ± 7.75 µm	101.6 mg/L	-	-	19 d	○	✓	[84]
Cladocera	*Daphnia magna*	neonate (<24 h)	PVC	-	-	2 ± 1 µm<br>50 ± 10 µm	2.05 mg/L<br>4.97 mg/L	-	-	48 h	✓	○	[95]
Cladocera	*Daphnia magna*	-	EAA	-	particle	103 nm	1.2 mg/L	-	-	21 d	✓	○	[99]
Cladocera	*Daphnia magna*	juvenile, adult	MPs mixtures	fluorescent	irregular particle	-40 µm	1 % MPs in food	-	-	48 h	✓	○	[88]
Decapoda	shrimp (*Penaeus monodon, Marsupenaeus japonicas, Litopenaeus vannamei*)	juvenile	PE	-	bead	5 µm	100 mg/L	-	-	48 h	✓	○	[117]
Decapoda	white-leg shrimp (Litopenaeus vannamei)	juvenile	PE	red fluorescent	particle	5 µm	50, 500, 5000 µg/L	-	-	48 h	✓	○	[118]

Order/ subclass	Biological species	Exposure phase	MPs type	Color	Shape	Size	Concentration	Type #	Concentration#	Exposure time	Gene-level changes*	Protein-level changes*	Reference
Decapoda	*Litopenaeus vannamei*	juvenile	PE	red fluorescent	sphere	10-22 μm	0.1-1 μg/g shrimp	-	-	7 d	✓	○	[119]
Decapoda	Pacific white shrimp (*Litopenaeus vannamei*)	juvenile	PE<br>PS<br>PP<br>PVC<br>PTFE	white	irregular particle	6-18 μm<br>100-200 μm<br>1.77-18 μm<br>1-13 μm<br>1-8 μm	1 mg/L	-	-	14 d	○	✓	[217]
Decapoda	Pacific white shrimp (*Litopenaeus vannamei*)	-	PS	-	sphere	100 nm	200, 2000 mg/kg	TiO_2	200, 2000 mg/kg	28 d	✓	○	[121]
Decapoda	Pacific whiteleg shrimp (*Litopenaeus vannamei*)	-	PS	-	sphere	500 nm	0.69 mg/L	BPA	0, 2 μg/L	14 d	✓	○	[216]
Decapoda	shrimp (*Macrobrachium nipponense*)	juvenile	PS	-	sphere	75 nm	5-40 mg/L	-	-	28 d	✓	○	[129]
Decapoda	river shrimp (*Macrobrachi*	juvenile	PS	-	sphere	75 nm	5-40 mg/L	-	-	28 d	✓	○	[235]

Order/subclass	Biological species	Exposure phase	MPs type	Color	Shape	Size	Concentration	Type#	Concentration#	Exposure time	Gene-level changes*	Protein-level changes*	Reference
	um nipponense)												
Decapoda	river prawn (*Macrobrachium nipponense*)	juvenile	PS	-	sphere	75 nm	5-40 mg/L	-	-	28 d	✓	○	[236]
Decapoda	oriental river prawn (*Macrobrachium nipponense*)	juvenile	PS	-	sphere	75 nm	5-40 mg/L	-	-	28 d	✓	○	[130]
Decapoda	oriental river prawn (*Macrobrachium nipponense*)	juvenile	PS	-	sphere	75 nm	5-40 mg/L	-	-	28 d	✓	○	[237]
Decapoda	prawn (*Macrobrachium nipponense*)	adult	PS	-	sphere	500 nm	0.04-40 mg/L	-	-	28 d	✓	○	[131]
Decapoda	oriental river prawn (*Macrobrachium nipponense*)	-	PS	red fluorescent	sphere	5 µm	2, 20 mg/L	-	-	28 d	✓	○	[132]
Decapoda	giant river prawn (*Macr*	juvenile	PS	-	sphere	0.5–1 µm		-	-	60 d	✓	○	[133]

Order/subclass	Biological species	Exposure phase	MPs type	Color	Shape	Size	Concentration	Type#	Concentration#	Exposure time	Gene-level changes*	Protein-level changes*	Reference
	obrachium rosenbergii)		PE		irregular flake	30-150 μm	10 mg/100 g food						
Decapoda	Chinese mitten crab (*Eriocheir sinensis*)	-	PS	-	sphere	200 nm, 1 μm	-	-	-	-	✓	○	[238]
Decapoda	Chinese mitten crab (*Eriocheir sinensis*)	juvenile	PS	virgin	sphere	5 μm	0.04-40 mg/L	-	-	21 d	✓	○	[135]
Decapoda	Chinese mitten crab (*Eriocheir sinensis*)	juvenile	PS	-	sphere	5 μm	0.04-40 mg/L	-	-	21 d	✓	○	[221]
Decapoda	Chinese mitten crab (*Eriocheir sinensis*)	juvenile	PS	fluorescent	sphere	5 μm	400 μg/L	Pb	0, 5, 50 μg/L	21 d	✓	○	[222]
Decapoda	shore crab (*Carcinus maenas*)	-	PS, PS-COOH, PS-NH_2	-	sphere	8 μm	10^6, 10^7 spheres/L	-	-	24 h	○	✓	[224]
Decapoda	crayfish (*Procambarus clarkii*)	-	PE	Nile red	powder	3-20 μm	0.5‰, 1‰, 2‰ weight in food	-	-	21 d	✓	○	[225]

Order/subclass	Biological species	Exposure phase	MPs type	Color	Shape	Size	Concentration	Type#	Concentration#	Exposure time	Gene-level changes*	Protein-level changes*	Reference
Decapoda	red crayfish (*Cherax quadricarinatus*)	-	PS	green fluorescent	sphere	80 nm	25, 250, 2500 µg/L	-	-	14 d	✓	○	[227]
Decapoda	redclaw crayfish (*Cherax quadricarinatus*)	juvenile	PS	fluorescent	sphere	200 nm	0.5, 5 mg/L	-	-	21 d	✓	○	[140]
Copepoda	*Tigriopus japonicus*	adult	PS	fluorescent	bead	50 nm	23, 230 µg/L	Hg	2, 10 µg/L	48 h	✓	○	[239]
Copepoda	*Tigriopus japonicus*	-	PS	fluorescent	bead	50 nm, 2 µm	0.5 µg/L, 100 mg/L	-	-	30 d	✓	○	[144]
Copepoda	*Tigriopus japonicus*	adult	PS	-	bead	50 nm, 10 µm	20 mg/L	-	-	24, 48 h	✓	○	[145]
Copepoda	*Tigriopus japonicus*	nauplius (<24 h)	PS	fluorescent	bead	6 µm	0.23 mg/L	-	-	2 generations	○	✓	[147]
Copepoda	*Parvocalanus crassirostris*	-	PET	-	particle	5–10 µm	20000 particles/mL	-	-	6 d	✓	○	[155]
Copepoda	*Paracyclopina nana*	-	PS	-	bead	0.05, 0.5, 6 µm	10 mg/L	-	-	24 h	○	✓	[156]
Anostraca	brine shrimp (*Artemia franciscana*)	nauplius	$PS\text{-}NH_2$	-	sphere	50 nm	0.1, 1 mg/L	-	-	48 h, 14 d	✓	○	[166]

Order/subclass	Biological species	Exposure phase	MPs type	Color	Shape	Size	Concentration	Type#	Concentration#	Exposure time	Gene-level changes*	Protein-level changes*	Reference
Anostraca	brine shrimp (*Artemia franciscana*)	juvenile	PS	nonfunctionalized	bead	1, 3, 6, 10 μm	1, 1000 particles/mL	-	-	96 h	✓	○	[169]
Anostraca	brine shrimp (*Artemia franciscana*)	-	PS	-	bead	4–6 μm	0.2, 2 mg/L	-	-	14 d	✓	○	[172]
Anostraca	brine shrimp (*Artemia salina*)	1, 2, 7, 14 d,	PS	-	particle	5 μm	1 mg/L	-	-	14 d	✓	○	[177]

MPs, microplastics; PS, polystyrene; PS-COOH, carboxylated-PS; PS-NH_2, amino-PS; PE, polyethylene; PVC, polyvinyl chloride; EAA, ethylene acrylic acid; PP, polypropylene; PTFE, polytetrafluoroethylene; PET, polyethylene terephthalate; Hg, mercury; Zn, zinc; TiO_2, titanium dioxide; Pb, lead; HA, humic acid; NOM, natural organic matter; FA, fulvic acid; BPA, bisphenol A; #, other contaminants; *, molecular response mechanisms.

References

1. Wang, Z.; Dong, H.; Wang, Y.; Ren, R.; Qin, X.; Wang, S. Effects of microplastics and their adsorption of cadmium as vectors on the cladoceran *Moina monogolica* Daday: Implications for plastic-ingesting organisms. *J. Hazard. Mater.* **2020**, 400, 123239, doi:10.1016/j.jhazmat.2020.123239.
2. Portugal, S.G.M.; Oses, C.A.B.; Thiago, M.G.R.; Branco, C.W.C. Uptake of Microplastics by a tropical freshwater Cladocera revealed by polyethylene terephthalate fluorescence. *Water, Air, & Soil Pollution* **2021**, 232, doi:10.1007/s11270-021-05291-0.
3. Liu, Q.; Liu, L.; Huang, J.; Gu, L.; Sun, Y.; Zhang, L.; Lyu, K.; Yang, Z. The response of life history defense of cladocerans under predation risk varies with the size and concentration of microplastics. *J. Hazard. Mater.* **2022**, 427, 127913, doi:10.1016/j.jhazmat.2021.127913.
4. Kim, J.; Rhee, J. Biochemical and physiological responses of the water flea *Moina macrocopa* to microplastics: a multigenerational study. *Mol. Cell. Toxicol.* **2021**, 17, 523-532, doi:10.1007/s13273-021-00162-5.
5. Jaikumar, G.; Baas, J.; Brun, N.R.; Vijver, M.G.; Bosker, T. Acute sensitivity of three Cladoceran species to different types of microplastics in combination with thermal stress. *Environ. Pollut.* **2018**, 239, 733-740, doi:10.1016/j.envpol.2018.04.069.
6. Jaikumar, G.; Brun, N.R.; Vijver, M.G.; Bosker, T. Reproductive toxicity of primary and secondary microplastics to three cladocerans during chronic exposure. *Environ. Pollut.* **2019**, 249, 638-646, doi:10.1016/j.envpol.2019.03.085.
7. Ziajahromi, S.; Kumar, A.; Neale, P.A.; Leusch, F.D.L. Impact of microplastic beads and fibers on waterflea (*Ceriodaphnia dubia*) survival, growth, and reproduction: Implications of single and mixture exposures. *Environ. Sci. Technol.* **2017**, 51, 13397-13406, doi:10.1021/acs.est.7b03574.
8. Nugnes, R.; Lavorgna, M.; Orlo, E.; Russo, C.; Isidori, M. Toxic impact of polystyrene microplastic particles in freshwater organisms. *Chemosphere* **2022**, 299, 134373, doi:10.1016/j.chemosphere.2022.134373.
9. Nugnes, R.; Russo, C.; Lavorgna, M.; Orlo, E.; Kundi, M.; Isidori, M. Polystyrene microplastic particles in combination with pesticides and antiviral drugs: Toxicity and genotoxicity in *Ceriodaphnia dubia*. *Environ. Pollut.* **2022**, 313, 120088, doi:10.1016/j.envpol.2022.120088.
10. Yoo, J.; Cho, H.; Jeon, M.; Jeong, C.; Jung, J.; Lee, Y. Effects of polystyrene in the brackish water flea *Diaphanosoma celebensis*: Size-dependent acute toxicity, ingestion, egestion, and antioxidant response. *Aquat. Toxicol.* **2021**, 235, 105821, doi:10.1016/j.aquatox.2021.105821.
11. Cho, H.; Jeong, C.; Lee, Y. Modulation of ecdysteroid and juvenile hormone signaling pathways by bisphenol analogues and polystyrene beads in the brackish water flea *Diaphanosoma celebensis*. *Comparative Biochemistry and Physiology Part C: Toxicology & Pharmacology* **2022**, 262, 109462, doi:10.1016/j.cbpc.2022.109462.
12. Yoo, J.; Jeon, M.; Lee, K.; Jung, J.; Jeong, C.; Lee, Y. The single and combined effects of mercury and polystyrene plastic beads on antioxidant-related systems in the brackish water flea: toxicological interaction depending on mercury species and plastic bead size. *Aquat. Toxicol.* **2022**, 252, 106325, doi:10.1016/j.aquatox.2022.106325.
13. Thi, D.D.; Miranda, A.; Trestrail, C.; De Souza, H.; Dinh, K.V.; Nugegoda, D. Antagonistic effects of copper and microplastics in single and binary mixtures on development and reproduction in the freshwater cladoceran *Daphnia carinata*. *Environ. Technol. Innov.* **2021**, 24, 102045, doi:10.1016/j.eti.2021.102045.
14. Liu, Z.; Li, Y.; Sepúlveda, M.S.; Jiang, Q.; Jiao, Y.; Chen, Q.; Huang, Y.; Tian, J.; Zhao, Y. Development of an adverse outcome pathway for nanoplastic toxicity in *Daphnia pulex* using proteomics. *Sci. Total Environ.* **2021**, 766, 144249, doi:10.1016/j.scitotenv.2020.144249.
15. Liu, Z.; Cai, M.; Wu, D.; Yu, P.; Jiao, Y.; Jiang, Q.; Zhao, Y. Effects of nanoplastics at predicted environmental concentration on *Daphnia pulex* after exposure through multiple generations. *Environ. Pollut.* **2020**, 256, 113506, doi:10.1016/j.envpol.2019.113506.
16. Zhang, W.; Liu, Z.; Tang, S.; Li, D.; Jiang, Q.; Zhang, T. Transcriptional response provides insights into the effect of chronic polystyrene nanoplastic exposure on *Daphnia pulex*. *Chemosphere* **2020**, 238, 124563, doi:10.1016/j.chemosphere.2019.124563.
17. Liu, Z.; Cai, M.; Yu, P.; Chen, M.; Wu, D.; Zhang, M.; Zhao, Y. Age-dependent survival, stress defense, and AMPK in *Daphnia pulex* after short-term exposure to a polystyrene nanoplastic. *Aquat. Toxicol.* **2018**, 204, 1-8, doi:10.1016/j.aquatox.2018.08.017.

18. Liu, Z.; Yu, P.; Cai, M.; Wu, D.; Zhang, M.; Huang, Y.; Zhao, Y. Polystyrene nanoplastic exposure induces immobilization, reproduction, and stress defense in the freshwater cladoceran *Daphnia pulex*. *Chemosphere* **2019**, 215, 74-81, doi:10.1016/j.chemosphere.2018.09.176.
19. Chenxi, Z.; Zhang, T.; Liu, X.; Gu, X.; Li, D.; Yin, J.; Jiang, Q.; Zhang, W. Changes in life-history traits, antioxidant defense, energy metabolism and molecular outcomes in the cladoceran *Daphnia pulex* after exposure to polystyrene microplastics. *Chemosphere* **2022**, 308, 136066, doi:10.1016/j.chemosphere.2022.136066.
20. Monikh, F.A.; Durão, M.; Kipriianov, P.V.; Huuskonen, H.; Kekäläinen, J.; Uusi-Heikkilä, S.; Uurasjärvi, E.; Akkanen, J.; Kortet, R. Chemical composition and particle size influence the toxicity of nanoscale plastic debris and their co-occurring benzo(α)pyrene in the model aquatic organisms *Daphnia magna* and *Danio rerio*. *NanoImpact* **2022**, 25, 100382, doi:10.1016/j.impact.2022.100382.
21. Ekvall, M.T.; Gimskog, I.; Hua, J.; Kelpsiene, E.; Lundqvist, M.; Cedervall, T. Size fractionation of high-density polyethylene breakdown nanoplastics reveals different toxic response in *Daphnia magna*. *Sci. Rep.* **2022**, 12, doi:10.1038/s41598-022-06991-1.
22. Rehse, S.; Kloas, W.; Zarfl, C. Short-term exposure with high concentrations of pristine microplastic particles leads to immobilisation of *Daphnia magna*. *Chemosphere* **2016**, 153, 91-99, doi:10.1016/j.chemosphere.2016.02.133.
23. Felten, V.; Toumi, H.; Masfaraud, J.; Billoir, E.; Camara, B.I.; Férard, J. Microplastics enhance *Daphnia magna* sensitivity to the pyrethroid insecticide deltamethrin: Effects on life history traits. *Sci. Total Environ.* **2020**, 714, 136567, doi:10.1016/j.scitotenv.2020.136567.
24. Zocchi, M.; Sommaruga, R. Microplastics modify the toxicity of glyphosate on *Daphnia magna*. *Sci. Total Environ.* **2019**, 697, 134194, doi:10.1016/j.scitotenv.2019.134194.
25. Ogonowski, M.; Schür, C.; Jarsén, Å.; Gorokhova, E. The effects of natural and anthropogenic microparticles on individual fitness in *Daphnia magna*. *PLoS One* **2016**, 11, e155063, doi:10.1371/journal.pone.0155063.
26. Song, J.; Kim, C.; Na, J.; Sivri, N.; Samanta, P.; Jung, J. Transgenerational effects of polyethylene microplastic fragments containing benzophenone-3 additive in *Daphnia magna*. *J. Hazard. Mater.* **2022**, 436, 129225, doi:10.1016/j.jhazmat.2022.129225.
27. Wang, P.; Li, Q.; Hui, J.; Xiang, Q.; Yan, H.; Chen, L. Metabolomics reveals the mechanism of polyethylene microplastic toxicity to *Daphnia magna*. *Chemosphere* **2022**, 307, 135887, doi:https://doi.org/10.1016/j.chemosphere.2022.135887.
28. Pan, Y.; Long, Y.; Hui, J.; Xiao, W.; Yin, J.; Li, Y.; Liu, D.; Tian, Q.; Chen, L. Microplastics can affect the trophic cascade strength and stability of plankton ecosystems via behavior-mediated indirect interactions. *J. Hazard. Mater.* **2022**, 430, 128415, doi:10.1016/j.jhazmat.2022.128415.
29. Na, J.; Song, J.; Achar, J.C.; Jung, J. Synergistic effect of microplastic fragments and benzophenone-3 additives on lethal and sublethal *Daphnia magna* toxicity. *J. Hazard. Mater.* **2021**, 402, 123845, doi:10.1016/j.jhazmat.2020.123845.
30. Castro, G.B.; Bernegossi, A.C.; Felipe, M.C.; Corbi, J.J. Is the development of *Daphnia magna* neonates affected by short-term exposure to polyethylene microplastics? *Journal of Environmental Science and Health, Part A* **2020**, 55, 935-946, doi:10.1080/10934529.2020.1756656.
31. Castro, G.B.; Bernegossi, A.C.; Pinheiro, F.R.; Corbi, J.J. The silent harm of polyethylene microplastics: Invertebrates growth inhibition as a warning of the microplastic pollution in continental waters. *Limnologica* **2022**, 93, 125964, doi:10.1016/j.limno.2022.125964.
32. Song, J.; Na, J.; An, D.; Jung, J. Role of benzophenone-3 additive in chronic toxicity of polyethylene microplastic fragments to *Daphnia magna*. *Sci. Total Environ.* **2021**, 800, 149638, doi:10.1016/j.scitotenv.2021.149638.
33. An, D.; Na, J.; Song, J.; Jung, J. Size-dependent chronic toxicity of fragmented polyethylene microplastics to *Daphnia magna*. *Chemosphere* **2021**, 271, 129591, doi:10.1016/j.chemosphere.2021.129591.
34. Amariei, G.; Rosal, R.; Fernández-Piñas, F.; Koelmans, A.A. Negative food dilution and positive biofilm carrier effects of microplastic ingestion by *D. magna* cause tipping points at the population level. *Environ. Pollut.* **2022**, 294, 118622, doi:10.1016/j.envpol.2021.118622.
35. Canniff, P.M.; Hoang, T.C. Microplastic ingestion by *Daphnia magna* and its enhancement on algal growth. *Sci. Total Environ.* **2018**, 633, 500-507, doi:10.1016/j.scitotenv.2018.03.176.
36. Frydkjær, C.K.; Iversen, N.; Roslev, P. Ingestion and egestion of microplastics by the Cladoceran *Daphnia magna*: Effects of regular and irregular shaped plastic and sorbed phenanthrene. *Bull. Environ. Contam. Toxicol.* **2017**, 99, 655-661, doi:10.1007/s00128-017-2186-3.

37. Renzi, M.; Grazioli, E.; Blašković, A. Effects of different microplastic types and surfactant-microplastic mixtures under fasting and feeding conditions: A case study on *Daphnia magna*. *Bull. Environ. Contam. Toxicol.* **2019**, 103, 367-373, doi:10.1007/s00128-019-02678-y.
38. Xu, E.G.; Cheong, R.S.; Liu, L.; Hernandez, L.M.; Azimzada, A.; Bayen, S.; Tufenkji, N. Primary and secondary plastic particles exhibit limited acute toxicity but chronic effects on *Daphnia magna*. *Environ. Sci. Technol.* **2020**, 54, 6859-6868, doi:10.1021/acs.est.0c00245.
39. Pikuda, O.; Xu, E.G.; Berk, D.; Tufenkji, N. Toxicity assessments of micro- and nanoplastics can be confounded by preservatives in commercial formulations. *Environ. Sci. Technol. Lett.* **2019**, 6, 21-25, doi:10.1021/acs.estlett.8b00614.
40. Heinlaan, M.; Kasemets, K.; Aruoja, V.; Blinova, I.; Bondarenko, O.; Lukjanova, A.; Khosrovyan, A.; Kurvet, I.; Pullerits, M.; Sihtmäe, M. et al. Hazard evaluation of polystyrene nanoplastic with nine bioassays did not show particle-specific acute toxicity. *Sci. Total Environ.* **2020**, 707, 136073, doi:10.1016/j.scitotenv.2019.136073.
41. De Felice, B.; Sugni, M.; Casati, L.; Parolini, M. Molecular, biochemical and behavioral responses of *Daphnia magna* under long-term exposure to polystyrene nanoplastics. *Environ. Int.* **2022**, 164, 107264, doi:10.1016/j.envint.2022.107264.
42. Zhang, F.; Wang, Z.; Wang, S.; Fang, H.; Wang, D. Aquatic behavior and toxicity of polystyrene nanoplastic particles with different functional groups: Complex roles of pH, dissolved organic carbon and divalent cations. *Chemosphere* **2019**, 228, 195-203, doi:10.1016/j.chemosphere.2019.04.115.
43. Mattsson, K.; Johnson, E.V.; Malmendal, A.; Linse, S.; Hansson, L.; Cedervall, T. Brain damage and behavioural disorders in fish induced by plastic nanoparticles delivered through the food chain. *Sci. Rep.* **2017**, 7, doi:10.1038/s41598-017-10813-0.
44. Kelpsiene, E.; Torstensson, O.; Ekvall, M.T.; Hansson, L.; Cedervall, T. Long-term exposure to nanoplastics reduces life-time in *Daphnia magna*. *Sci. Rep.* **2020**, 10, doi:10.1038/s41598-020-63028-1.
45. Ma, Y.; Huang, A.; Cao, S.; Sun, F.; Wang, L.; Guo, H.; Ji, R. Effects of nanoplastics and microplastics on toxicity, bioaccumulation, and environmental fate of phenanthrene in fresh water. *Environmental pollution* **2016**, 219, 166-173, doi:10.1016/j.envpol.2016.10.061.
46. Frankel, R.; Ekvall, M.T.; Kelpsiene, E.; Hansson, L.; Cedervall, T. Controlled protein mediated aggregation of polystyrene nanoplastics does not reduce toxicity towards *Daphnia magna*. *Environmental Science: Nano* **2020**, 7, 1518-1524, doi:10.1039/C9EN01236B.
47. Besseling, E.; Wang, B.; Lürling, M.; Koelmans, A.A. Nanoplastic sffects growth of *S. obliquus* and reproduction of *D. magna*. *Environ. Sci. Technol.* **2014**, 48, 12336-12343, doi:10.1021/es503001d.
48. Lyu, K.; Yu, B.; Li, D.; Gu, L.; Yang, Z. Increased food availability reducing the harmful effects of microplastics strongly depends on the size of microplastics. *J. Hazard. Mater.* **2022**, 437, 129375, doi:10.1016/j.jhazmat.2022.129375.
49. Nogueira, D.J.; Silva, A.C.D.O.; Da Silva, M.L.N.; Vicentini, D.S.; Matias, W.G. Individual and combined multigenerational effects induced by polystyrene nanoplastic and glyphosate in *Daphnia magna* (Strauss, 1820). *Sci. Total Environ.* **2022**, 811, 151360, doi:10.1016/j.scitotenv.2021.151360.
50. Lin, W.; Jiang, R.; Hu, S.; Xiao, X.; Wu, J.; Wei, S.; Xiong, Y.; Ouyang, G. Investigating the toxicities of different functionalized polystyrene nanoplastics on *Daphnia magna*. *Ecotox. Environ. Safe.* **2019**, 180, 509-516, doi:10.1016/j.ecoenv.2019.05.036.
51. Rist, S.; Baun, A.; Hartmann, N.B. Ingestion of micro- and nanoplastics in *Daphnia magna* – Quantification of body burdens and assessment of feeding rates and reproduction. *Environ. Pollut.* **2017**, 228, 398-407, doi:10.1016/j.envpol.2017.05.048.
52. Nasser, F.; Lynch, I. Secreted protein eco-corona mediates uptake and impacts of polystyrene nanoparticles on *Daphnia magna*. *J. Proteomics* **2016**, 137, 45-51, doi:10.1016/j.jprot.2015.09.005.
53. Tong, L.; Song, K.; Wang, Y.; Yang, J.; Ji, J.; Lu, J.; Chen, Z.; Zhang, W. Zinc oxide nanoparticles dissolution and toxicity enhancement by polystyrene microplastics under sunlight irradiation. *Chemosphere* **2022**, 299, 134421, doi:10.1016/j.chemosphere.2022.134421.
54. Tong, L.; Duan, P.; Tian, X.; Huang, J.; Ji, J.; Chen, Z.; Yang, J.; Yu, H.; Zhang, W. Polystyrene microplastics sunlight-induce oxidative dissolution, chemical transformation and toxicity enhancement of silver nanoparticles. *Sci. Total Environ.* **2022**, 827, 154180, doi:10.1016/j.scitotenv.2022.154180.
55. Reynolds, A.; Giltrap, M.; Chambers, G. Evaluation of non-invasive toxicological analysis of nano-polystyrene in relative in vivo conditions to *D. magna*. *Environmental Science: Nano* **2019**, 6, 2832-2849, doi:10.1039/C9EN00434C.
56. Chen, C.C.; Shi, Y.; Zhu, Y.; Zeng, J.; Qian, W.; Zhou, S.; Ma, J.; Pan, K.; Jiang, Y.; Tao, Y. et al. Combined toxicity of polystyrene microplastics and ammonium perfluorooctanoate to *Daphnia*

magna: Mediation of intestinal blockage. *Water Res.* **2022**, 219, 118536, doi:10.1016/j.watres.2022.118536.
57. Fadare, O.O.; Wan, B.; Guo, L.; Xin, Y.; Qin, W.; Yang, Y. Humic acid alleviates the toxicity of polystyrene nanoplastic particles to *Daphnia magna*. *Environmental science. Nano* **2019**, 6, 1466-1477, doi:10.1039/C8EN01457D.
58. Fadare, O.O.; Wan, B.; Liu, K.; Yang, Y.; Zhao, L.; Guo, L. Eco-corona vs protein corona: Effects of humic substances on corona formation and nanoplastic particle toxicity in *Daphnia magna*. *Environ. Sci. Technol.* **2020**, 54, 8001-8009, doi:10.1021/acs.est.0c00615.
59. Chang, M.; Zhang, C.; Li, M.; Dong, J.; Li, C.; Liu, J.; Verheyen, J.; Stoks, R. Warming, temperature fluctuations and thermal evolution change the effects of microplastics at an environmentally relevant concentration. *Environ. Pollut.* **2022**, 292, 118363, doi:10.1016/j.envpol.2021.118363.
60. Kim, D.; Chae, Y.; An, Y. Mixture Toxicity of nickel and microplastics with different functional groups on *Daphnia magna*. *Environ. Sci. Technol.* **2017**, 51, 12852-12858, doi:10.1021/acs.est.7b03732.
61. Saavedra, J.; Stoll, S.; Slaveykova, V.I. Influence of nanoplastic surface charge on eco-corona formation, aggregation and toxicity to freshwater zooplankton. *Environ. Pollut.* **2019**, 252, 715-722, doi:10.1016/j.envpol.2019.05.135.
62. Wu, J.; Jiang, R.; Lin, W.; Ouyang, G. Effect of salinity and humic acid on the aggregation and toxicity of polystyrene nanoplastics with different functional groups and charges. *Environ. Pollut.* **2019**, 245, 836-843, doi:10.1016/j.envpol.2018.11.055.
63. Abdolahpur Monikh, F.; Vijver, M.G.; Guo, Z.; Zhang, P.; Darbha, G.K.; Peijnenburg, W.J.G.M. Metal sorption onto nanoscale plastic debris and trojan horse effects in *Daphnia magna*: Role of dissolved organic matter. *Water Res.* **2020**, 186, 116410, doi:10.1016/j.watres.2020.116410.
64. Sadler, D.E.; Brunner, F.S.; Plaistow, S.J. Temperature and clone-dependent effects of microplastics on immunity and life history in *Daphnia magna*. *Environ. Pollut.* **2019**, 255, 113178, doi:10.1016/j.envpol.2019.113178.
65. Huang, C.; Chu, T.; Kuo, C.; Hong, M.; Chen, Y.; Chen, B. Effects of Microplastics on reproduction and growth of freshwater live feeds *Daphnia magna*. *Fishes* **2022**, 7, 181, doi:10.3390/fishes7040181.
66. Varg, J.E.; Bergvall, C.; Svanbäck, R. The Stressful effects of microplastics associated with chromium (VI) on the microbiota of *Daphnia Magna*. *Front. Environ. Sci.* **2022**, 10, doi:10.3389/fenvs.2022.875512.
67. Kim, J.; Haque, M.N.; Lee, S.; Lee, D.; Rhee, J. Exposure to environmentally relevant concentrations of polystyrene microplastics increases hexavalent chromium toxicity in aquatic animals. *Toxics* **2022**, 10, 563, doi:10.3390/toxics10100563.
68. Horton, A.A.; Vijver, M.G.; Lahive, E.; Spurgeon, D.J.; Svendsen, C.; Heutink, R.; van Bodegom, P.M.; Baas, J. Acute toxicity of organic pesticides to *Daphnia magna* is unchanged by co-exposure to polystyrene microplastics. *Ecotox. Environ. Safe.* **2018**, 166, 26-34, doi:10.1016/j.ecoenv.2018.09.052.
69. Xiang, X.; Zhou, J.; Lin, S.; Zhang, N.; Abulipizi, G.; Chen, G.; Li, Z. Dual drive acute lethal toxicity of methylene blue to *Daphnia magna* by polystyrene microplastics and light. *Sci. Total Environ.* **2022**, 840, 156681, doi:10.1016/j.scitotenv.2022.156681.
70. Bosker, T.; Olthof, G.; Vijver, M.G.; Baas, J.; Barmentlo, S.H. Significant decline of *Daphnia magna* population biomass due to microplastic exposure. *Environ. Pollut.* **2019**, 250, 669-675, doi:10.1016/j.envpol.2019.04.067.
71. De Felice, B.; Sabatini, V.; Antenucci, S.; Gattoni, G.; Santo, N.; Bacchetta, R.; Ortenzi, M.A.; Parolini, M. Polystyrene microplastics ingestion induced behavioral effects to the cladoceran *Daphnia magna*. *Chemosphere* **2019**, 231, 423-431, doi:10.1016/j.chemosphere.2019.05.115.
72. Zhang, P.; Yan, Z.; Lu, G.; Ji, Y. Single and combined effects of microplastics and roxithromycin on *Daphnia magna*. *Environ. Sci. Pollut. Res.* **2019**, 26, 17010-17020, doi:10.1007/s11356-019-05031-2.
73. Lyu, K.; Cao, C.; Li, D.; Akbar, S.; Yang, Z. The thermal regime modifies the response of aquatic keystone species *Daphnia* to microplastics: Evidence from population fitness, accumulation, histopathological analysis and candidate gene expression. *Sci. Total Environ.* **2021**, 783, 147154, doi:10.1016/j.scitotenv.2021.147154.
74. Lin, H.; Yuan, Y.; Jiang, X.; Zou, J.; Xia, X.; Luo, S. Bioavailability quantification and uptake mechanisms of pyrene associated with different-sized microplastics to *Daphnia magna*. *Sci. Total Environ.* **2021**, 797, 149201, doi:10.1016/j.scitotenv.2021.149201.
75. Aljaibachi, R.; Callaghan, A. Impact of polystyrene microplastics on *Daphnia magna* mortality and reproduction in relation to food availability. *PeerJ* **2018**, 6, e4601, doi:10.7717/peerj.4601.
76. Lee, Y.; Yoon, D.; Lee, Y.H.; Kwak, J.I.; An, Y.; Lee, J.; Park, J.C. Combined exposure to microplastics and zinc produces sex-specific responses in the water flea *Daphnia magna*. *J. Hazard. Mater.* **2021**, 420, 126652, doi:10.1016/j.jhazmat.2021.126652.

77. Hiltunen, M.; Vehniäinen, E.; Kukkonen, J.V.K. Interacting effects of simulated eutrophication, temperature increase, and microplastic exposure on *Daphnia*. *Environ. Res.* **2021**, 192, 110304, doi:10.1016/j.envres.2020.110304.
78. Liu, Z.; Zhu, Y.; Lv, S.; Shi, Y.; Dong, S.; Yan, D.; Zhu, X.; Peng, R.; Keller, A.A.; Huang, Y. Quantifying the dynamics of polystyrene microplastics UV-aging process. *Environ. Sci. Technol. Lett.* **2022**, 9, 50-56, doi:10.1021/acs.estlett.1c00888.
79. Liu, J.; Yang, H.; Meng, Q.; Feng, Q.; Yan, Z.; Liu, J.; Liu, Z.; Zhou, Z. Intergenerational and biological effects of roxithromycin and polystyrene microplastics to *Daphnia magna*. *Aquat. Toxicol.* **2022**, 248, 106192, doi:10.1016/j.aquatox.2022.106192.
80. Yin, C.; Yang, X.; Zhao, T.; Watson, P.; Yang, F.; Liu, H. Changes of the acute and chronic toxicity of three antimicrobial agents to *Daphnia magna* in the presence/absence of micro-polystyrene. *Environ. Pollut.* **2020**, 263, 114551, doi:10.1016/j.envpol.2020.114551.
81. Eltemsah, Y.S.; Bøhn, T. Acute and chronic effects of polystyrene microplastics on juvenile and adult *Daphnia magna*. *Environ. Pollut.* **2019**, 254, 112919, doi:10.1016/j.envpol.2019.07.087.
82. Schwarzer, M.; Brehm, J.; Vollmer, M.; Jasinski, J.; Xu, C.; Zainuddin, S.; Fröhlich, T.; Schott, M.; Greiner, A.; Scheibel, T. et al. Shape, size, and polymer dependent effects of microplastics on *Daphnia magna*. *J. Hazard. Mater.* **2022**, 426, 128136, doi:10.1016/j.jhazmat.2021.128136.
83. Yuan, W.; Zhou, Y.; Chen, Y.; Liu, X.; Wang, J. Toxicological effects of microplastics and heavy metals on the *Daphnia magna*. *Sci. Total Environ.* **2020**, 746, 141254, doi:10.1016/j.scitotenv.2020.141254.
84. Trotter, B.; Wilde, M.V.; Brehm, J.; Dafni, E.; Aliu, A.; Arnold, G.J.; Fröhlich, T.; Laforsch, C. Long-term exposure of *Daphnia magna* to polystyrene microplastic (PS-MP) leads to alterations of the proteome, morphology and life-history. *Sci. Total Environ.* **2021**, 795, 148822, doi:10.1016/j.scitotenv.2021.148822.
85. Aljaibachi, R.; Laird, W.B.; Stevens, F.; Callaghan, A. Impacts of polystyrene microplastics on *Daphnia magna*: A laboratory and a mesocosm study. *Sci. Total Environ.* **2020**, 705, 135800, doi:10.1016/j.scitotenv.2019.135800.
86. Serra, T.; Barcelona, A.; Pous, N.; Salvadó, V.; Colomer, J. Synergistic effects of water temperature, microplastics and ammonium as second and third order stressors on *Daphnia magna*. *Environ. Pollut.* **2020**, 267, 115439, doi:10.1016/j.envpol.2020.115439.
87. Magester, S.; Barcelona, A.; Colomer, J.; Serra, T. Vertical distribution of microplastics in water bodies causes sublethal effects and changes in *Daphnia magna* swimming behaviour. *Ecotox. Environ. Safe.* **2021**, 228, 113001, doi:10.1016/j.ecoenv.2021.113001.
88. Imhof, H.K.; Rusek, J.; Thiel, M.; Wolinska, J.; Laforsch, C. Do microplastic particles affect *Daphnia magna* at the morphological, life history and molecular level? *PLoS One* **2017**, 12, e187590, doi:10.1371/journal.pone.0187590.
89. Schür, C.; Weil, C.; Baum, M.; Wallraff, J.; Schreier, M.; Oehlmann, J.; Wagner, M. Incubation in Wastewater Reduces the Multigenerational Effects of Microplastics in *Daphnia magna*. *Environ. Sci. Technol.* **2021**, 55, 2491-2499, doi:10.1021/acs.est.0c07911.
90. Schür, C.; Zipp, S.; Thalau, T.; Wagner, M. Microplastics but not natural particles induce multigenerational effects in *Daphnia magna*. *Environ. Pollut.* **2020**, 260, 113904, doi:10.1016/j.envpol.2019.113904.
91. SÖNMEZ, V.Z.; ERCAN, N.; SİVRİ, N. Toxic effects of ester based polymers on *Daphnia magna*: A laboratory microcosm study. *Carpath. J. Earth Environ. Sci.* **2022**, 17, 35-47, doi:10.26471/cjees/2022/017/198.
92. Zimmermann, L.; Göttlich, S.; Oehlmann, J.; Wagner, M.; Völker, C. What are the drivers of microplastic toxicity? Comparing the toxicity of plastic chemicals and particles to *Daphnia magna*. *Environ. Pollut.* **2020**, 267, 115392, doi:10.1016/j.envpol.2020.115392.
93. Piazza, V.; Uheida, A.; Gambardella, C.; Garaventa, F.; Faimali, M.; Dutta, J. Ecosafety screening of photo-fenton process for the degradation of microplastics in water. *Front. Mar. Sci.* **2022**, 8, doi:10.3389/fmars.2021.791431.
94. Kim, D.; Kim, H.; An, Y. Effects of synthetic and natural microfibers on *Daphnia magna*–Are they dependent on microfiber type? *Aquat. Toxicol.* **2021**, 240, 105968, doi:10.1016/j.aquatox.2021.105968.
95. Liu, Y.; Zhang, J.; Zhao, H.; Cai, J.; Sultan, Y.; Fang, H.; Zhang, B.; Ma, J. Effects of polyvinyl chloride microplastics on reproduction, oxidative stress and reproduction and detoxification-related genes in *Daphnia magna*. *Comparative Biochemistry and Physiology Part C: Toxicology & Pharmacology* **2022**, 254, 109269, doi:10.1016/j.cbpc.2022.109269.

96. Schrank, I.; Trotter, B.; Dummert, J.; Scholz-Böttcher, B.M.; Löder, M.G.J.; Laforsch, C. Effects of microplastic particles and leaching additive on the life history and morphology of *Daphnia magna*. *Environ. Pollut.* **2019**, 255, 113233, doi:10.1016/j.envpol.2019.113233.
97. Tourinho, P.S.; Silva, A.R.R.; Santos, C.S.A.; Prodana, M.; Ferreira, V.; Habibullah, G.; Kočí, V.; Gestel, C.A.M.; Loureiro, S. Microplastic fibers increase sublethal effects of AgNP and $AgNO_3$ in *Daphnia magna* by changing cellular energy allocation. *Environ. Toxicol. Chem.* **2022**, 41, 896-904, doi:10.1002/etc.5136.
98. Jemec, A.; Horvat, P.; Kunej, U.; Bele, M.; Kržan, A. Uptake and effects of microplastic textile fibers on freshwater crustacean *Daphnia magna*. *Environ. Pollut.* **2016**, 219, 201-209, doi:10.1016/j.envpol.2016.10.037.
99. Coady, K.K.; Burgoon, L.; Doskey, C.; Davis, J.W. Assessment of transcriptomic and apical responses of *Daphnia magna* exposed to a polyethylene microplastic in a 21-d chronic study. *Environ. Toxicol. Chem.* **2020**, 39, 1578-1589, doi:10.1002/etc.4745.
100. Martins, A.; Guilhermino, L. Transgenerational effects and recovery of microplastics exposure in model populations of the freshwater cladoceran *Daphnia magna* Straus. *Sci. Total Environ.* **2018**, 631-632, 421-428, doi:10.1016/j.scitotenv.2018.03.054.
101. Guilhermino, L.; Martins, A.; Cunha, S.; Fernandes, J.O. Long-term adverse effects of microplastics on *Daphnia magna* reproduction and population growth rate at increased water temperature and light intensity: Combined effects of stressors and interactions. *Sci. Total Environ.* **2021**, 784, 147082, doi:10.1016/j.scitotenv.2021.147082.
102. Martins, A.; Da Silva, D.D.; Silva, R.; Carvalho, F.; Guilhermino, L. Long-term effects of lithium and lithium-microplastic mixtures on the model species *Daphnia magna*: Toxicological interactions and implications to 'One Health'. *Sci. Total Environ.* **2022**, 838, 155934, doi:10.1016/j.scitotenv.2022.155934.
103. Gerdes, Z.; Ogonowski, M.; Nybom, I.; Ek, C.; Adolfsson-Erici, M.; Barth, A.; Gorokhova, E. Microplastic-mediated transport of PCBs? A depuration study with *Daphnia magna*. *PLoS One* **2019**, 14, e205378, doi:10.1371/journal.pone.0205378.
104. Booth, A.M.; Hansen, B.H.; Frenzel, M.; Johnsen, H.; Altin, D. Uptake and toxicity of methylmethacrylate-based nanoplastic particles in aquatic organisms. *Environ. Toxicol. Chem.* **2016**, 35, 1641-1649, doi:10.1002/etc.3076.
105. Pacheco, A.; Martins, A.; Guilhermino, L. Toxicological interactions induced by chronic exposure to gold nanoparticles and microplastics mixtures in *Daphnia magna*. *Sci. Total Environ.* **2018**, 628-629, 474-483, doi:10.1016/j.scitotenv.2018.02.081.
106. Gökçe, D.; Şeftalicioğlu, M.D.; Erden, B.A.; Köytepe, S. Chronic and acute water-soluble microplastics uptake and effects on growth and reproduction of *Daphnia magna*. *Water, Air, & Soil Pollution* **2022**, 233, doi:10.1007/s11270-022-05907-z.
107. Khosrovyan, A.; Kahru, A. Virgin and UV-weathered polyamide microplastics posed no effect on the survival and reproduction of *Daphnia magna*. *PeerJ* **2022**, 10, e13533, doi:10.7717/peerj.13533.
108. Schell, T.; Martinez Perez, S.; Dafouz, R.; Hurley, R.; Vighi, M.; Rico, A. Effects of polyester fibers and car tire particles on freshwater invertebrates. *Environ. Toxicol. Chem.* **2022**, 41, 1555-1567, doi:10.1002/etc.5337.
109. Cunningham, B.; Harper, B.; Brander, S.; Harper, S. Toxicity of micro and nano tire particles and leachate for model freshwater organisms. *J. Hazard. Mater.* **2022**, 429, 128319, doi:https://doi.org/10.1016/j.jhazmat.2022.128319.
110. Kokalj, A.J.; Kunej, U.; Skalar, T. Screening study of four environmentally relevant microplastic pollutants: Uptake and effects on *Daphnia magna* and *Artemia franciscana*. *Chemosphere* **2018**, 208, 522-529, doi:10.1016/j.chemosphere.2018.05.172.
111. Kalčíková, G.; Skalar, T.; Marolt, G.; Jemec Kokalj, A. An environmental concentration of aged microplastics with adsorbed silver significantly affects aquatic organisms. *Water Res.* **2020**, 175, 115644, doi:10.1016/j.watres.2020.115644.
112. Vroom, R.J.E.; Koelmans, A.A.; Besseling, E.; Halsband, C. Aging of microplastics promotes their ingestion by marine zooplankton. *Environ. Pollut.* **2017**, 231, 987-996, doi:10.1016/j.envpol.2017.08.088.
113. Woods, M.N.; Hong, T.J.; Baughman, D.; Andrews, G.; Fields, D.M.; Matrai, P.A. Accumulation and effects of microplastic fibers in American lobster larvae (*Homarus americanus*). *Mar. Pollut. Bull.* **2020**, 157, 111280, doi:10.1016/j.marpolbul.2020.111280.

114. Bergami, E.; Manno, C.; Cappello, S.; Vannuccini, M.L.; Corsi, I. Nanoplastics affect moulting and faecal pellet sinking in Antarctic krill (*Euphausia superba*) juveniles. *Environ. Int.* **2020**, 143, 105999, doi:10.1016/j.envint.2020.105999.
115. Rowlands, E.; Galloway, T.; Cole, M.; Lewis, C.; Peck, V.; Thorpe, S.; Manno, C. The Effects of Combined Ocean Acidification and Nanoplastic Exposures on the embryonic development of Antarctic krill. *Front. Mar. Sci.* **2021**, 8, doi:10.3389/fmars.2021.709763.
116. Dawson, A.; Huston, W.; Kawaguchi, S.; King, C.; Cropp, R.; Wild, S.; Eisenmann, P.; Townsend, K.; Bengtson Nash, S. Uptake and Depuration Kinetics Influence Microplastic bioaccumulation and toxicity in Antarctic krill (*Euphausia superba*). *Environ. Sci. Technol.* **2018**, 52, 3195-3201, doi:10.1021/acs.est.7b05759.
117. Wang, Z.; Fan, L.; Wang, J.; Zhou, J.; Ye, Q.; Zhang, L.; Xu, G.; Zou, J. Impacts of microplastics on three different juvenile shrimps: Investigating the organism response distinction. *Environ. Res.* **2021**, 198, 110466, doi:10.1016/j.envres.2020.110466.
118. Wang, Z.; Fan, L.; Wang, J.; Xie, S.; Zhang, C.; Zhou, J.; Zhang, L.; Xu, G.; Zou, J. Insight into the immune and microbial response of the white-leg shrimp *Litopenaeus vannamei* to microplastics. *Mar. Environ. Res.* **2021**, 169, 105377, doi:10.1016/j.marenvres.2021.105377.
119. Hsieh, S.; Wu, Y.; Xu, R.; Chen, Y.; Chen, C.; Singhania, R.R.; Dong, C. Effect of polyethylene microplastics on oxidative stress and histopathology damages in *Litopenaeus vannamei*. *Environ. Pollut.* **2021**, 288, 117800, doi:10.1016/j.envpol.2021.117800.
120. Chae, Y.; Kim, D.; Choi, M.; Cho, Y.; An, Y. Impact of nano-sized plastic on the nutritional value and gut microbiota of whiteleg shrimp *Litopenaeus vannamei* via dietary exposure. *Environ. Int.* **2019**, 130, 104848, doi:10.1016/j.envint.2019.05.042.
121. Zhu, X.; Teng, J.; Xu, E.G.; Zhao, J.; Shan, E.; Sun, C.; Wang, Q. Toxicokinetics and toxicodynamics of plastic and metallic nanoparticles: A comparative study in shrimp. *Environ. Pollut.* **2022**, 312, 120069, doi:10.1016/j.envpol.2022.120069.
122. Hariharan, G.; Purvaja, R.; Anandavelu, I.; Robin, R.S.; Ramesh, R. Ingestion and toxic impacts of weathered polyethylene (wPE) microplastics and stress defensive responses in whiteleg shrimp (*Penaeus vannamei*). *Chemosphere* **2022**, 300, 134487, doi:10.1016/j.chemosphere.2022.134487.
123. Gray, A.D.; Weinstein, J.E. Size- and shape-dependent effects of microplastic particles on adult daggerblade grass shrimp (*Palaemonetes pugio*). *Environ. Toxicol. Chem.* **2017**, 36, 3074-3080, doi:10.1002/etc.3881.
124. Leads, R.R.; Burnett, K.G.; Weinstein, J.E. The Effect of Microplastic ingestion on survival of the grass shrimp *Palaemonetes pugio* (Holthuis, 1949) challenged with vibrio campbellii. *Environ. Toxicol. Chem.* **2019**, 38, 2233-2242, doi:10.1002/etc.4545.
125. Gray, A.D.; Weinstein, J.E.; Riegerix, R.C. Assessment of acute toxicity and developmental transformation impacts of polyethylene microbead exposure on larval daggerblade grass shrimp (*Palaemon pugio*). *Sci. Rep.* **2022**, 12, 6967, doi:10.1038/s41598-022-10999-y.
126. Eom, H.; Haque, M.N.; Lee, S.; Rhee, J. Exposure to metals premixed with microplastics increases toxicity through bioconcentration and impairs antioxidant defense and cholinergic response in a marine mysid. *Comparative Biochemistry and Physiology Part C: Toxicology & Pharmacology* **2021**, 249, 109142, doi:10.1016/j.cbpc.2021.109142.
127. Lee, D.; Lee, S.; Rhee, J. Consistent exposure to microplastics induces age-specific physiological and biochemical changes in a marine mysid. *Mar. Pollut. Bull.* **2021**, 162, 111850, doi:10.1016/j.marpolbul.2020.111850.
128. Wang, X.; Liu, L.; Zheng, H.; Wang, M.; Fu, Y.; Luo, X.; Li, F.; Wang, Z. Polystyrene microplastics impaired the feeding and swimming behavior of mysid shrimp *Neomysis japonica*. *Mar. Pollut. Bull.* **2020**, 150, 110660, doi:10.1016/j.marpolbul.2019.110660.
129. Li, Y.; Liu, Z.; Li, M.; Jiang, Q.; Wu, D.; Huang, Y.; Jiao, Y.; Zhang, M.; Zhao, Y. Effects of nanoplastics on antioxidant and immune enzyme activities and related gene expression in juvenile *Macrobrachium nipponense*. *J. Hazard. Mater.* **2020**, 398, 122990, doi:10.1016/j.jhazmat.2020.122990.
130. Li, Y.; Liu, Z.; Yang, Y.; Jiang, Q.; Wu, D.; Huang, Y.; Jiao, Y.; Chen, Q.; Huang, Y.; Zhao, Y. Effects of nanoplastics on energy metabolism in the oriental river prawn (*Macrobrachium nipponense*). *Environ. Pollut.* **2021**, 268, 115890, doi:10.1016/j.envpol.2020.115890.
131. Fan, W.; Yang, P.; Qiao, Y.; Su, M.; Zhang, G. Polystyrene nanoplastics decrease molting and induce oxidative stress in adult *Macrobrachium nipponense*. *Fish Shellfish Immunol.* **2022**, 122, 419-425, doi:10.1016/j.fsi.2022.02.028.

132. Sun, S.; Jin, Y.; Luo, P.; Shi, X. Polystyrene microplastics induced male reproductive toxicity and transgenerational effects in freshwater prawn. *Sci. Total Environ.* **2022**, 842, 156820, doi:10.1016/j.scitotenv.2022.156820.
133. Jaikumar, I.M.; Periyakali, S.B.; Rajendran, U.; Joen-Rong, S.; Thanasekaran, J.; Tsorng-Harn, F. Effects of microplastics, polystyrene, and polyethylene on antioxidants, metabolic enzymes, HSP-70, and myostatin expressions in the giant river prawn *Macrobrachium rosenbergii*: Impact on survival and growth. *Arch. Environ. Contam. Toxicol.* **2021**, 80, 645-658, doi:10.1007/s00244-021-00833-3.
134. Villegas, L.; Cabrera, M.; Moulatlet, G.M.; Capparelli, M. The synergistic effect of microplastic and malathion exposure on fiddler crab *Minuca ecuadoriensis* microplastic bioaccumulation and survival. *Mar. Pollut. Bull.* **2022**, 175, 113336, doi:10.1016/j.marpolbul.2022.113336.
135. Yu, P.; Liu, Z.; Wu, D.; Chen, M.; Lv, W.; Zhao, Y. Accumulation of polystyrene microplastics in juvenile *Eriocheir sinensis* and oxidative stress effects in the liver. *Aquat. Toxicol.* **2018**, 200, 28-36, doi:10.1016/j.aquatox.2018.04.015.
136. Watts, A.J.R.; Urbina, M.A.; Corr, S.; Lewis, C.; Galloway, T.S. Ingestion of plastic microfibers by the crab *Carcinus maenas* and its effect on food consumption and energy balance. *Environ. Sci. Technol.* **2015**, 49, 14597-14604, doi:10.1021/acs.est.5b04026.
137. Torn, K. Microplastics uptake and accumulation in the digestive system of the mud crab *Rhithropanopeus harrisii*. *P. Est. Acad. Sci.* **2020**, 69, 35, doi:10.3176/proc.2020.1.04.
138. Devriese, L.I.; De Witte, B.; Vethaak, A.D.; Hostens, K.; Leslie, H.A. Bioaccumulation of PCBs from microplastics in Norway lobster (*Nephrops norvegicus*): An experimental study. *Chemosphere* **2017**, 186, 10-16, doi:10.1016/j.chemosphere.2017.07.121.
139. Welden, N.A.C.; Cowie, P.R. Long-term microplastic retention causes reduced body condition in the langoustine, *Nephrops norvegicus*. *Environmental pollution* **2016**, 218, 895-900, doi:10.1016/j.envpol.2016.08.020.
140. Chen, Q.; Lv, W.; Jiao, Y.; Liu, Z.; Li, Y.; Cai, M.; Wu, D.; Zhou, W.; Zhao, Y. Effects of exposure to waterborne polystyrene microspheres on lipid metabolism in the hepatopancreas of juvenile redclaw crayfish, *Cherax quadricarinatus*. *Aquat. Toxicol.* **2020**, 224, 105497, doi:10.1016/j.aquatox.2020.105497.
141. Raju, P.; Santhanam, P.; Pandian, S.S.; Divya, M.; Arunkrishnan, A.; Devi, K.N.; Ananth, S.; Roopavathy, J.; Perumal, P. Impact of polystyrene microplastics on major marine primary (phytoplankton) and secondary producers (copepod). *Arch. Microbiol.* **2021**, 204, 84.
142. Svetlichny, L.; Isinibilir, M.; Mykitchak, T.; Eryalçın, K.M.; Türkeri, E.E.; Yuksel, E.; Kideys, A.E. Microplastic consumption and physiological response in *Acartia clausi* and *Centropages typicus*: Possible roles of feeding mechanisms. *Reg. Stud. Mar. Sci.* **2021**, 43, 101650, doi:https://doi.org/10.1016/j.rsma.2021.101650.
143. Lee, K.; Shim, W.J.; Kwon, O.Y.; Kang, J. Size-dependent effects of micro polystyrene particles in the marine copepod *Tigriopus japonicus*. *Environ. Sci. Technol.* **2013**, 47, 11278-11283, doi:10.1021/es401932b.
144. Kim, K.; Yoon, H.; Choi, J.S.; Jung, Y.; Park, J. Chronic effects of nano and microplastics on reproduction and development of marine copepod *Tigriopus japonicus*. *Ecotox. Environ. Safe.* **2022**, 243, 113962, doi:10.1016/j.ecoenv.2022.113962.
145. Choi, J.S.; Hong, S.H.; Park, J. Evaluation of microplastic toxicity in accordance with different sizes and exposure times in the marine copepod *Tigriopus japonicus*. *Mar. Environ. Res.* **2020**, 153, 104838, doi:10.1016/j.marenvres.2019.104838.
146. Li, Z.; Zhou, H.; Liu, Y.; Zhan, J.; Li, W.; Yang, K.; Yi, X. Acute and chronic combined effect of polystyrene microplastics and dibutyl phthalate on the marine copepod *Tigriopus japonicus*. *Chemosphere* **2020**, 261, 127711, doi:10.1016/j.chemosphere.2020.127711.
147. Zhang, C.; Jeong, C.; Lee, J.; Wang, D.; Wang, M. Transgenerational proteome plasticity in resilience of a marine copepod in response to environmentally relevant concentrations of microplastics. *Environ. Sci. Technol.* **2019**, 53, 8426-8436, doi:10.1021/acs.est.9b02525.
148. 148. Shi, W.; Guo, H.; Wang, J.; Han, X.; Cai, W. Adverse effects of co-exposure to Cd and microplastic in *Tigriopus japonicus*. *International Journal of Environmental Research and Public Health* **2022**, 19, 13215, doi:10.3390/ijerph192013215.
149. Yu, J.; Tian, J.; Xu, R.; Zhang, Z.; Yang, G.; Wang, X.; Lai, J.; Chen, R. Effects of microplastics exposure on ingestion, fecundity, development, and dimethylsulfide production in *Tigriopus japonicus* (Harpacticoida, copepod). *Environ. Pollut.* **2020**, 267, 115429, doi:10.1016/j.envpol.2020.115429.

150. Yang, K.; Jing, S.; Liu, Y.; Zhou, H.; Liu, Y.; Yan, M.; Yi, X.; Liu, R. Acute toxicity of tire wear particles, leachates and toxicity identification evaluation of leachates to the marine copepod, *Tigriopus japonicus*. *Chemosphere* **2022**, 297, 134099, doi:10.1016/j.chemosphere.2022.134099.
151. Sun, J.; Yang, S.; Zhou, G.; Zhang, K.; Lu, Y.; Jin, Q.; Lam, P.K.S.; Leung, K.M.Y.; He, Y. Release of microplastics from discarded surgical masks and their adverse impacts on the marine copepod *Tigriopus japonicus*. *Environ. Sci. Technol. Lett.* **2021**, 8, 1065-1070, doi:10.1021/acs.estlett.1c00748.
152. Tong, H.; Zhong, X.; Duan, Z.; Yi, X.; Cheng, F.; Xu, W.; Yang, X. Micro- and nanoplastics released from biodegradable and conventional plastics during degradation: Formation, aging factors, and toxicity. *Sci. Total Environ.* **2022**, 833, 155275, doi:10.1016/j.scitotenv.2022.155275.
153. Parlapiano, I.; Biandolino, F.; Grattagliano, A.; Ruscito, A.; Lofrano, G.; Libralato, G.; Trifuoggi, M.; Albarano, L.; Prato, E. Multi-endpoint effects of derelict tubular mussel plastic nets on *Tigriopus fulvus*. *Environ. Sci. Pollut. Res.* **2022**, 29, 83554-83566, doi:10.1007/s11356-022-21569-0.
154. Jaapar, A.N.; Md Amin, R.; Bhubalan, K.; Sohaimi, E.S. Changes in the development and reproductive output of *Nitokra lacustris pacifica* (Crustacea: Copepoda) Yeatman, 1983 under short and long term exposure to synthetic and biodegradable microbeads. *J. Polym. Environ.* **2021**, 29, 4060-4072, doi:10.1007/s10924-021-02165-8.
155. Heindler, F.M.; Alajmi, F.; Huerlimann, R.; Zeng, C.; Newman, S.J.; Vamvounis, G.; van Herwerden, L. Toxic effects of polyethylene terephthalate microparticles and Di(2-ethylhexyl)phthalate on the calanoid copepod, *Parvocalanus crassirostris*. *Ecotox. Environ. Safe.* **2017**, 141, 298-305, doi:10.1016/j.ecoenv.2017.03.029.
156. Jeong, C.; Kang, H.; Lee, M.; Kim, D.; Han, J.; Hwang, D.; Souissi, S.; Lee, S.; Shin, K.; Park, H.G. et al. Adverse effects of microplastics and oxidative stress-induced MAPK/Nrf2 pathway-mediated defense mechanisms in the marine copepod *Paracyclopina nana*. *Sci. Rep.* **2017**, 7, doi:10.1038/srep41323.
157. Bellas, J.; Gil, I. Polyethylene microplastics increase the toxicity of chlorpyrifos to the marine copepod *Acartia tonsa*. *Environ. Pollut.* **2020**, 260, 114059, doi:10.1016/j.envpol.2020.114059.
158. Syberg, K.; Nielsen, A.; Khan, F.R.; Banta, G.T.; Palmqvist, A.; Jepsen, P.M. Microplastic potentiates triclosan toxicity to the marine copepod *Acartia tonsa* (Dana). *Journal of Toxicology and Environmental Health, Part A* **2017**, 80, 1369-1371, doi:10.1080/15287394.2017.1385046.
159. Shore, E.A.; DeMayo, J.A.; Pespeni, M.H. Microplastics reduce net population growth and fecal pellet sinking rates for the marine copepod, *Acartia tonsa*. *Environ. Pollut.* **2021**, 284, 117379, doi:10.1016/j.envpol.2021.117379.
160. Koski, M.; Søndergaard, J.; Christensen, A.M.; Nielsen, T.G. Effect of environmentally relevant concentrations of potentially toxic microplastic on coastal copepods. *Aquat. Toxicol.* **2021**, 230, 105713, doi:10.1016/j.aquatox.2020.105713.
161. Sørensen, L.; Rogers, E.; Altin, D.; Salaberria, I.; Booth, A.M. Sorption of PAHs to microplastic and their bioavailability and toxicity to marine copepods under co-exposure conditions. *Environ. Pollut.* **2020**, 258, 113844, doi:10.1016/j.envpol.2019.113844.
162. Cole, M.; Coppock, R.; Lindeque, P.K.; Altin, D.; Reed, S.; Pond, D.W.; Sørensen, L.; Galloway, T.S.; Booth, A.M. Effects of nylon microplastic on feeding, lipid accumulation, and moulting in a coldwater copepod. *Environ. Sci. Technol.* **2019**, 53, 7075-7082, doi:10.1021/acs.est.9b01853.
163. Rodríguez-Torres, R.; Almeda, R.; Kristiansen, M.; Rist, S.; Winding, M.S.; Nielsen, T.G. Ingestion and impact of microplastics on arctic *Calanus* copepods. *Aquat. Toxicol.* **2020**, 228, 105631, doi:10.1016/j.aquatox.2020.105631.
164. Cole, M.; Lindeque, P.; Fileman, E.; Halsband, C.; Galloway, T.S. The Impact of polystyrene microplastics on feeding, function and fecundity in the marine copepod *Calanus helgolandicus*. *Environ. Sci. Technol.* **2015**, 49, 1130-1137, doi:10.1021/es504525u.
165. Lins, T.F.; O'Brien, A.M.; Kose, T.; Rochman, C.M.; Sinton, D. Toxicity of nanoplastics to zooplankton is influenced by temperature, salinity, and natural particulate matter. *Environmental Science: Nano* **2022**, 9, 2678-2690, doi:10.1039/D2EN00123C.
166. Varó, I.; Perini, A.; Torreblanca, A.; Garcia, Y.; Bergami, E.; Vannuccini, M.L.; Corsi, I. Time-dependent effects of polystyrene nanoparticles in brine shrimp *Artemia franciscana* at physiological, biochemical and molecular levels. *Sci. Total Environ.* **2019**, 675, 570-580, doi:10.1016/j.scitotenv.2019.04.157.
167. Gambardella, C.; Morgana, S.; Ferrando, S.; Bramini, M.; Piazza, V.; Costa, E.; Garaventa, F.; Faimali, M. Effects of polystyrene microbeads in marine planktonic crustaceans. *Ecotox. Environ. Safe.* **2017**, 145, 250-257, doi:10.1016/j.ecoenv.2017.07.036.

168. Kim, L.; Cui, R.; Il Kwak, J.; An, Y. Trophic transfer of nanoplastics through a microalgae−crustacean–small yellow croaker food chain: Inhibition of digestive enzyme activity in fish. *J. Hazard. Mater.* **2022**, 440, 129715, doi:10.1016/j.jhazmat.2022.129715.
169. Eom, H.; Nam, S.; Rhee, J. Polystyrene microplastics induce mortality through acute cell stress and inhibition of cholinergic activity in a brine shrimp. *Mol. Cell. Toxicol.* **2020**, 16, 233-243, doi:10.1007/s13273-020-00088-4.
170. Peixoto, D.; Amorim, J.; Pinheiro, C.; Oliva-Teles, L.; Varó, I.; de Medeiros Rocha, R.; Vieira, M.N. Uptake and effects of different concentrations of spherical polymer microparticles on *Artemia franciscana*. *Ecotox. Environ. Safe.* **2019**, 176, 211-218, doi:10.1016/j.ecoenv.2019.03.100.
171. Peixoto, D.; Torreblanca, A.; Pereira, S.; Vieira, M.N.; Varó, I. Effect of short-term exposure to fluorescent red polymer microspheres on *Artemia franciscana* nauplii and juveniles. *Environ. Sci. Pollut. Res.* **2022**, 29, 6080-6092, doi:10.1007/s11356-021-15992-y.
172. Han, X.; Zheng, Y.; Dai, C.; Duan, H.; Gao, M.; Ali, M.R.; Sui, L. Effect of polystyrene microplastics and temperature on growth, intestinal histology and immune responses of brine shrimp *Artemia franciscana*. *J. Oceanol. Limnol.* **2021**, 39, 979-988, doi:10.1007/s00343-020-0118-2.
173. Di Giannantonio, M.; Gambardella, C.; Miroglio, R.; Costa, E.; Sbrana, F.; Smerieri, M.; Carraro, G.; Utzeri, R.; Faimali, M.; Garaventa, F. Ecotoxicity of polyvinylidene difluoride (PVDF) and polylactic acid (PLA) microplastics in marine zooplankton. *Toxics* **2022**, 10, 479, doi:10.3390/toxics10080479.
174. Kim, L.; Kim, S.A.; Kim, T.H.; Kim, J.; An, Y. Synthetic and natural microfibers induce gut damage in the brine shrimp *Artemia franciscana*. *Aquat. Toxicol.* **2021**, 232, 105748, doi:10.1016/j.aquatox.2021.105748.
175. Wang, Y.; Zhang, D.; Zhang, M.; Mu, J.; Ding, G.; Mao, Z.; Cao, Y.; Jin, F.; Cong, Y.; Wang, L. et al. Effects of ingested polystyrene microplastics on brine shrimp, *Artemia parthenogenetica*. *Environ. Pollut.* **2019**, 244, 715-722, doi:10.1016/j.envpol.2018.10.024.
176. Li, H.; Chen, H.; Wang, J.; Li, J.; Liu, S.; Tu, J.; Chen, Y.; Zong, Y.; Zhang, P.; Wang, Z. et al. Influence of microplastics on the growth and the intestinal microbiota composition of brine shrimp. *Front. Microbiol.* **2021**, 12, doi:10.3389/fmicb.2021.717272.
177. Suman, T.Y.; Jia, P.; Li, W.; Junaid, M.; Xin, G.; Wang, Y.; Pei, D. Acute and chronic effects of polystyrene microplastics on brine shrimp: First evidence highlighting the molecular mechanism through transcriptome analysis. *J. Hazard. Mater.* **2020**, 400, 123220, doi:10.1016/j.jhazmat.2020.123220.
178. Mishra, P.; Vinayagam, S.; Duraisamy, K.; Patil, S.R.; Godbole, J.; Mohan, A.; Mukherjee, A.; Chandrasekaran, N. Distinctive impact of polystyrene nano-spherules as an emergent pollutant toward the environment. *Environ. Sci. Pollut. Res.* **2019**, 26, 1537-1547, doi:10.1007/s11356-018-3698-z.
179. Thiagarajan, V.; Alex, S.A.; Seenivasan, R.; Chandrasekaran, N.; Mukherjee, A. Toxicity evaluation of nano-TiO_2 in the presence of functionalized microplastics at two trophic levels: Algae and crustaceans. *Sci. Total Environ.* **2021**, 784, 147262, doi:10.1016/j.scitotenv.2021.147262.
180. Jeyavani, J.; Sibiya, A.; Bhavaniramya, S.; Mahboob, S.; Al-Ghanim, K.A.; Nisa, Z.; Riaz, M.N.; Nicoletti, M.; Govindarajan, M.; Vaseeharan, B. Toxicity evaluation of polypropylene microplastic on marine microcrustacean *Artemia salina*: An analysis of implications and vulnerability. *Chemosphere* **2022**, 296, 133990, doi:https://doi.org/10.1016/j.chemosphere.2022.133990.
181. Albendín, M.G.; Aranda, V.; Coello, M.D.; González-Gómez, C.; Rodríguez-Barroso, R.; Quiroga, J.M.; Arellano, J.M. Pharmaceutical products and pesticides toxicity associated with microplastics (polyvinyl chloride) in *Artemia salina*. *International Journal of Environmental Research and Public Health* **2021**, 18, 10773, doi:10.3390/ijerph182010773.
182. Bruck, S.; Ford, A.T. Chronic ingestion of polystyrene microparticles in low doses has no effect on food consumption and growth to the intertidal amphipod *Echinogammarus marinus*? *Environ. Pollut.* **2018**, 233, 1125-1130, doi:10.1016/j.envpol.2017.10.015.
183. Au, S.Y.; Bruce, T.F.; Bridges, W.C.; Klaine, S.J. Responses of *Hyalella azteca* to acute and chronic microplastic exposures. *Environ. Toxicol. Chem.* **2015**, 34, 2564-2572, doi:10.1002/etc.3093.
184. Khan, F.R.; Halle, L.L.; Palmqvist, A. Acute and long-term toxicity of micronized car tire wear particles to *Hyalella azteca*. *Aquat. Toxicol.* **2019**, 213, 105216, doi:10.1016/j.aquatox.2019.05.018.
185. Kalinkina, N.M.; Zobkov, M.B.; Zobkova, M.V.; Galakhina, N.E. Assessment of microplastic size range and ingestion intensity by *Gmelinoides fasciatus* stebbing, an invasive species of Lake Onego. *Environ. Toxicol. Chem.* **2022**, 41, 184-192, doi:10.1002/etc.5257.

186. Rani-Borges, B.; Meitern, R.; Teesalu, P.; Raudna-Kristoffersen, M.; Kreitsberg, R.; Heinlaan, M.; Tuvikene, A.; Ivask, A. Effects of environmentally relevant concentrations of microplastics on amphipods. *Chemosphere* **2022**, 309, 136599, doi:10.1016/j.chemosphere.2022.136599.
187. Mateos-Cárdenas, A.; O'Halloran, J.; van Pelt, F.N.A.M.; Jansen, M.A.K. Beyond plastic microbeads – Short-term feeding of cellulose and polyester microfibers to the freshwater amphipod *Gammarus duebeni*. *Sci. Total Environ.* **2021**, 753, 141859, doi:10.1016/j.scitotenv.2020.141859.
188. Blarer, P.; Burkhardt-Holm, P. Microplastics affect assimilation efficiency in the freshwater amphipod *Gammarus fossarum*. *Environ. Sci. Pollut. Res.* **2016**, 23, 23522-23532, doi:10.1007/s11356-016-7584-2.
189. Straub, S.; Hirsch, P.E.; Burkhardt-Holm, P. Biodegradable and petroleum-based microplastics do not differ in their ingestion and excretion but in their biological effects in a freshwater invertebrate *Gammarus fossarum*. *International Journal of Environmental Research and Public Health* **2017**, 14, 774, doi:10.3390/ijerph14070774.
190. Götz, A.; Beggel, S.; Geist, J. Dietary exposure to four sizes of spherical polystyrene, polylactide and silica nanoparticles does not affect mortality, behaviour, feeding and energy assimilation of *Gammarus roeseli*. *Ecotox. Environ. Safe.* **2022**, 238, 113581, doi:10.1016/j.ecoenv.2022.113581.
191. Bartonitz, A.; Anyanwu, I.N.; Geist, J.; Imhof, H.K.; Reichel, J.; Graßmann, J.; Drewes, J.E.; Beggel, S. Modulation of PAH toxicity on the freshwater organism *G. roeseli* by microparticles. *Environ. Pollut.* **2020**, 260, 113999, doi:10.1016/j.envpol.2020.113999.
192. Weber, A.; Scherer, C.; Brennholt, N.; Reifferscheid, G.; Wagner, M. PET microplastics do not negatively affect the survival, development, metabolism and feeding activity of the freshwater invertebrate *Gammarus pulex*. *Environ. Pollut.* **2018**, 234, 181-189, doi:10.1016/j.envpol.2017.11.014.
193. Redondo-Hasselerharm, P.E.; Falahudin, D.; Peeters, E.T.H.M.; Koelmans, A.A. Microplastic effect thresholds for freshwater benthic macroinvertebrates. *Environ. Sci. Technol.* **2018**, 52, 2278-2286, doi:10.1021/acs.est.7b05367.
194. Yip, Y.J.; Lee, S.S.C.; Neo, M.L.; Teo, S.L.; Valiyaveettil, S. A comparative investigation of toxicity of three polymer nanoparticles on acorn barnacle (*Amphibalanus amphitrite*). *Sci. Total Environ.* **2022**, 806, 150965, doi:10.1016/j.scitotenv.2021.150965.
195. Yu, S.; Chan, B.K.K. Intergenerational microplastics impact the intertidal barnacle *Amphibalanus amphitrite* during the planktonic larval and benthic adult stages. *Environ. Pollut.* **2020**, 267, 115560, doi:10.1016/j.envpol.2020.115560.
196. Yu, S.; Chan, B.K.K. Effects of polystyrene microplastics on larval development, settlement, and metamorphosis of the intertidal barnacle *Amphibalanus amphitrite*. *Ecotox. Environ. Safe.* **2020**, 194, 110362, doi:10.1016/j.ecoenv.2020.110362.
197. Nousheen, R.; Rittschof, D.; Hashmi, I. Toxic effects of pristine and aged polystyrene microplastics on selective and continuous larval culture of acorn barnacle *Amphibalanus amphitrite*. *Environ. Toxicol. Pharmacol.* **2022**, 94, 103912, doi:10.1016/j.etap.2022.103912.
198. 198. Yu, S.; Nakaoka, M.; Chan, B.K.K. The gut retention time of microplastics in barnacle naupliar larvae from different climatic zones and marine habitats. *Environ. Pollut.* **2021**, 268, 115865, doi:10.1016/j.envpol.2020.115865.
199. Bhargava, S.; Chen Lee, S.S.; Min Ying, L.S.; Neo, M.L.; Lay-Ming Teo, S.; Valiyaveettil, S. Fate of nanoplastics in marine larvae: A case study using barnacles, *Amphibalanus amphitrite*. *ACS Sustain. Chem. Eng.* **2018**, 6, 6932-6940, doi:10.1021/acssuschemeng.8b00766.
200. Hämer, J.; Gutow, L.; Köhler, A.; Saborowski, R. Fate of Microplastics in the marine isopod *Idotea emarginata*. *Environ. Sci. Technol.* **2014**, 48, 13451-13458, doi:10.1021/es501385y.
201. Chen, Q.; Li, Y.; Li, B. Is color a matter of concern during microplastic exposure to *Scenedesmus obliquus* and *Daphnia magna*? *J. Hazard. Mater.* **2020**, 383, 121224, doi:10.1016/j.jhazmat.2019.121224.
202. 202. Colomer, J.; Müller, M.F.; Barcelona, A.; Serra, T. Mediated food and hydrodynamics on the ingestion of microplastics by *Daphnia magna*. *Environ. Pollut.* **2019**, 251, 434-441, doi:10.1016/j.envpol.2019.05.034.
203. 203. Cole, M.; Lindeque, P.; Fileman, E.; Halsband, C.; Goodhead, R.; Moger, J.; Galloway, T.S. Microplastic ingestion by zooplankton. *Environ. Sci. Technol.* **2013**, 47, 6646-6655, doi:10.1021/es400663f.
204. Cheng, Y.; Wang, J.; Yi, X.; Li, L.; Liu, X.; Ru, S. Low microalgae availability increases the ingestion rates and potential effects of microplastics on marine copepod *Pseudodiaptomus annandalei*. *Mar. Pollut. Bull.* **2020**, 152, 110919, doi:10.1016/j.marpolbul.2020.110919.

205. Everaert, G.; Vlaeminck, K.; Vandegehuchte, M.B.; Janssen, C.R. Effects of microplastic on the population dynamics of a marine copepod: Insights from a laboratory experiment and a mechanistic model. *Environ. Toxicol. Chem.* **2022**, 41, 1663-1674, doi:10.1002/etc.5336.
206. Almeda, R.; Rodriguez-Torres, R.; Rist, S.; Winding, M.H.S.; Stief, P.; Hansen, B.H.; Nielsen, T.G. Microplastics do not increase bioaccumulation of petroleum hydrocarbons in Arctic zooplankton but trigger feeding suppression under co-exposure conditions. *Sci. Total Environ.* **2021**, 751, 141264, doi:10.1016/j.scitotenv.2020.141264.
207. Isinibilir, M.; Svetlichny, L.; Mykitchak, T.; Türkeri, E.E.; Eryalçın, K.M.; Doğan, O.; Can, G.; Yüksel, E.; Kideys, A.E. Microplastic consumption and its effect on respiration rate and motility of *Calanus helgolandicus* from the Marmara Sea. *Front. Mar. Sci.* **2020**, 7, doi:10.3389/fmars.2020.603321.
208. Coppock, R.L.; Galloway, T.S.; Cole, M.; Fileman, E.S.; Queirós, A.M.; Lindeque, P.K. Microplastics alter feeding selectivity and faecal density in the copepod, *Calanus helgolandicus*. *Sci. Total Environ.* **2019**, 687, 780-789, doi:10.1016/j.scitotenv.2019.06.009.
209. Procter, J.; Hopkins, F.E.; Fileman, E.S.; Lindeque, P.K. Smells good enough to eat: Dimethyl sulfide (DMS) enhances copepod ingestion of microplastics. *Mar. Pollut. Bull.* **2019**, 138, 1-6, doi:10.1016/j.marpolbul.2018.11.014.
210. Mateos-Cárdenas, A.; Moroney, A.V.D.G.; van Pelt, F.N.A.M.; O'Halloran, J.; Jansen, M.A.K. Trophic transfer of microplastics in a model freshwater microcosm; lack of a consumer avoidance response. *Food Webs* **2022**, 31, e228, doi:10.1016/j.fooweb.2022.e00228.
211. Kratina, P.; Watts, T.J.; Green, D.S.; Kordas, R.L.; O'Gorman, E.J. Interactive effects of warming and microplastics on metabolism but not feeding rates of a key freshwater detritivore. *Environ. Pollut.* **2019**, 255, 113259, doi:10.1016/j.envpol.2019.113259.
212. Cunningham, E.M.; Cuthbert, R.N.; Coughlan, N.E.; Kregting, L.; Cairnduff, V.; Dick, J.T.A. Microplastics do not affect the feeding rates of a marine predator. *Sci. Total Environ.* **2021**, 779, 146487, doi:10.1016/j.scitotenv.2021.146487.
213. Cunningham, E.M.; Mundye, A.; Kregting, L.; Dick, J.T.A.; Crump, A.; Riddell, G.; Arnott, G. Animal contests and microplastics: evidence of disrupted behaviour in hermit crabs *Pagurus bernhardus*. *R. Soc. Open Sci.* **2021**, 8, doi:10.1098/rsos.211089.
214. Wang, Y.; Mao, Z.; Zhang, M.; Ding, G.; Sun, J.; Du, M.; Liu, Q.; Cong, Y.; Jin, F.; Zhang, W. et al. The uptake and elimination of polystyrene microplastics by the brine shrimp, *Artemia parthenogenetica*, and its impact on its feeding behavior and intestinal histology. *Chemosphere* **2019**, 234, 123-131, doi:10.1016/j.chemosphere.2019.05.267.
215. Korez, `.; Gutow, L.; Saborowski, R. Feeding and digestion of the marine isopod *Idotea emarginata* challenged by poor food quality and microplastics. *Comparative Biochemistry and Physiology Part C: Toxicology & Pharmacology* **2019**, 226, 108586, doi:10.1016/j.cbpc.2019.108586.
216. Han, Y.; Shi, W.; Tang, Y.; Zhou, W.; Sun, H.; Zhang, J.; Yan, M.; Hu, L.; Liu, G. Microplastics and bisphenol A hamper gonadal development of whiteleg shrimp (*Litopenaeus vannamei*) by interfering with metabolism and disrupting hormone regulation. *Sci. Total Environ.* **2022**, 810, 152354, doi:10.1016/j.scitotenv.2021.152354.
217. Duan, Y.; Xiong, D.; Wang, Y.; Zhang, Z.; Li, H.; Dong, H.; Zhang, J. Toxicological effects of microplastics in *Litopenaeus vannamei* as indicated by an integrated microbiome, proteomic and metabolomic approach. *The Science of the total environment* **2021**, 761, 143311, doi:10.1016/j.scitotenv.2020.143311.
218. Korez, `.; Gutow, L.; Saborowski, R. Fishing in troubled waters: Limited stress response to natural and synthetic microparticles in brown shrimp (*Crangon crangon*). *Environ. Pollut.* **2022**, 302, 119023, doi:https://doi.org/10.1016/j.envpol.2022.119023.
219. Nobre, C.R.; Moreno, B.B.; Alves, A.V.; de Lima, R.J.; Fontes, M.K.; Campos, B.G.; Silva, L.; Almeida, D.L.; Abessa, D.; Choueri, R.B. et al. Combined effects of polyethylene spiked with the antimicrobial triclosan on the swamp ghost crab (*Ucides cordatus*; Linnaeus, 1763). *Chemosphere* **2022**, 304, 135169, doi:10.1016/j.chemosphere.2022.135169.
220. Da Silva, L.F.; Nobre, C.R.; Moreno, B.B.; Pereira, C.D.S.; de Souza Abessa, D.M.; Choueri, R.B.; Gusso-Choueri, P.K.; Cesar, A. Non-destructive biomarkers can reveal effects of the association of microplastics and pharmaceuticals or personal care products. *Mar. Pollut. Bull.* **2022**, 177, 113469, doi:10.1016/j.marpolbul.2022.113469.
221. Liu, Z.; Yu, P.; Cai, M.; Wu, D.; Zhang, M.; Chen, M.; Zhao, Y. Effects of microplastics on the innate immunity and intestinal microflora of juvenile *Eriocheir sinensis*. *Sci. Total Environ.* **2019**, 685, 836-846, doi:10.1016/j.scitotenv.2019.06.265.

222. Yang, Z.; Zhu, L.; Liu, J.; Cheng, Y.; Waiho, K.; Chen, A.; Wang, Y. Polystyrene microplastics increase Pb bioaccumulation and health damage in the Chinese mitten crab *Eriocheir sinensis*. *The Science of the total environment* **2022**, 829, 154586, doi:10.1016/j.scitotenv.2022.154586.
223. Wang, T.; Hu, M.; Xu, G.; Shi, H.; Leung, J.Y.S.; Wang, Y. Microplastic accumulation via trophic transfer: Can a predatory crab counter the adverse effects of microplastics by body defence? *Sci. Total Environ.* **2021**, 754, 142099, doi:10.1016/j.scitotenv.2020.142099.
224. Watts, A.J.R.; Urbina, M.A.; Goodhead, R.; Moger, J.; Lewis, C.; Galloway, T.S. Effect of Microplastic on the Gills of the Shore Crab *Carcinus maenas*. *Environ. Sci. Technol.* **2016**, 50, 5364-5369, doi:10.1021/acs.est.6b01187.
225. Zhang, X.; Jin, Z.; Shen, M.; Chang, Z.; Yu, G.; Wang, L.; Xia, X. Accumulation of polyethylene microplastics induces oxidative stress, microbiome dysbiosis and immunoregulation in crayfish. *Fish Shellfish Immunol.* **2022**, 125, 276-284, doi:10.1016/j.fsi.2022.05.005.
226. Han, M.; Gao, T.; Liu, G.; Zhu, C.; Zhang, T.; Sun, M.; Li, J.; Ji, F.; Si, Q.; Jiang, Q. The effect of a polystyrene nanoplastic on the intestinal microbes and oxidative stress defense of the freshwater crayfish, *Procambarus clarkii*. *The Science of the total environment* **2022**, 833, 155722, doi:10.1016/j.scitotenv.2022.155722.
227. Cheng, H.; Dai, Y.; Ruan, X.; Duan, X.; Zhang, C.; Li, L.; Huang, F.; Shan, J.; Liang, K.; Jia, X. et al. Effects of nanoplastic exposure on the immunity and metabolism of red crayfish (*Cherax quadricarinatus*) based on high-throughput sequencing. *Ecotox. Environ. Safe.* **2022**, 245, 114114, doi:10.1016/j.ecoenv.2022.114114.
228. Saborowski, R.; Korez, `.; Riesbeck, S.; Weidung, M.; Bickmeyer, U.; Gutow, L. Shrimp and microplastics: A case study with the Atlantic ditch shrimp *Palaemon varians*. *Ecotox. Environ. Safe.* **2022**, 234, 113394, doi:10.1016/j.ecoenv.2022.113394.
229. Liu, Z.; Huang, Y.; Jiao, Y.; Chen, Q.; Wu, D.; Yu, P.; Li, Y.; Cai, M.; Zhao, Y. Polystyrene nanoplastic induces ROS production and affects the MAPK-HIF-1/NFkB-mediated antioxidant system in *Daphnia pulex*. *Aquat. Toxicol.* **2020**, 220, 105420, doi:10.1016/j.aquatox.2020.105420.
230. Hoffschröer, N.; Grassl, N.; Steinmetz, A.; Sziegoleit, L.; Koch, M.; Zeis, B. Microplastic burden in *Daphnia* is aggravated by elevated temperatures. *Zoology* **2021**, 144, 125881, doi:10.1016/j.zool.2020.125881.
231. Liu, Z.; Li, Y.; Pérez, E.; Jiang, Q.; Chen, Q.; Jiao, Y.; Huang, Y.; Yang, Y.; Zhao, Y. Polystyrene nanoplastic induces oxidative stress, immune defense, and glycometabolism change in *Daphnia pulex*: Application of transcriptome profiling in risk assessment of nanoplastics. *J. Hazard. Mater.* **2021**, 402, 123778, doi:10.1016/j.jhazmat.2020.123778.
232. Liu, Z.; Jiao, Y.; Chen, Q.; Li, Y.; Tian, J.; Huang, Y.; Cai, M.; Wu, D.; Zhao, Y. Two sigma and two mu class genes of glutathione S-transferase in the waterflea *Daphnia pulex*: Molecular characterization and transcriptional response to nanoplastic exposure. *Chemosphere* **2020**, 248, 126065, doi:10.1016/j.chemosphere.2020.126065.
233. Wu, D.; Liu, Z.; Cai, M.; Jiao, Y.; Li, Y.; Chen, Q.; Zhao, Y. Molecular characterisation of cytochrome P450 enzymes in waterflea (*Daphnia pulex*) and their expression regulation by polystyrene nanoplastics. *Aquat. Toxicol.* **2019**, 217, 105350, doi:10.1016/j.aquatox.2019.105350.
234. Tang, J.; Wang, X.; Yin, J.; Han, Y.; Yang, J.; Lu, X.; Xie, T.; Akbar, S.; Lyu, K.; Yang, Z. Molecular characterization of thioredoxin reductase in waterflea *Daphnia magna* and its expression regulation by polystyrene microplastics. *Aquat. Toxicol.* **2019**, 208, 90-97, doi:10.1016/j.aquatox.2019.01.001.
235. Li, Y.; Liu, Z.; Jiang, Q.; Ye, Y.; Zhao, Y. Effects of nanoplastic on cell apoptosis and ion regulation in the gills of *Macrobrachium nipponense*. *Environ. Pollut.* **2022**, 300, 118989, doi:10.1016/j.envpol.2022.118989.
236. Li, Y.; Du, X.; Liu, Z.; Zhang, M.; Huang, Y.; Tian, J.; Jiang, Q.; Zhao, Y. Two genes related to reproductive development in the juvenile prawn, *Macrobrachium nipponense*: Molecular characterization and transcriptional response to nanoplastic exposure. *Chemosphere* **2021**, 281, 130827, doi:10.1016/j.chemosphere.2021.130827.
237. Li, Y.; Du, X.; Jiang, Q.; Huang, Y.; Zhao, Y. Effects of nanoplastic exposure on the growth performance and molecular characterization of growth-associated genes in juvenile *Macrobrachium nipponense*. *Comparative Biochemistry and Physiology Part C: Toxicology & Pharmacology* **2022**, 254, 109278, doi:10.1016/j.cbpc.2022.109278.
238. Nan, X.; Jin, X.; Song, Y.; Zhou, K.; Qin, Y.; Wang, Q.; Li, W. Effect of polystyrene nanoplastics on cell apoptosis, glucose metabolism, and antibacterial immunity of *Eriocheir sinensis*. *Environ. Pollut.* **2022**, 311, 119960, doi:10.1016/j.envpol.2022.119960.

239. Xie, D.; Wei, H.; Lee, J.; Wang, M. Mercury can be transported into marine copepod by polystyrene nanoplastics but is not bioaccumulated: An increased risk? *Environ. Pollut.* **2022**, 303, 119170, doi:10.1016/j.envpol.2022.119170.
